# Copper-Catalyzed Asymmetric Dearomative [3+2] Cycloaddition of Nitroheteroarenes with Azomethines

**DOI:** 10.3390/molecules28062765

**Published:** 2023-03-19

**Authors:** Yan Chen, Jian-Qiang Zhao, Yan-Ping Zhang, Ming-Qiang Zhou, Xiao-Mei Zhang, Wei-Cheng Yuan

**Affiliations:** 1 National Engineering Research Center of Chiral Drugs, Chengdu Institute of Organic Chemistry, Chinese Academy of Sciences, Chengdu 610041, China; 2 Innovation Research Center of Chiral Drugs, Institute for Advanced Study, Chengdu University, Chengdu 610106, China; 3 University of Chinese Academy of Sciences, Beijing 100049, China; 4 Department of Chemistry, Xihua University, Chengdu 610039, China

**Keywords:** dearomatization, asymmetric catalysis, 3-nitroindoles, 2-nitrobenzofurans, azomethine ylides

## Abstract

Catalytic asymmetric dearomative [3+2] cycloaddition of *α*-imino *γ*-lactones with either 3-nitroindoles or 2-nitrobenzofurans by using a chiral copper complex as the catalyst was developed. A wide range of structurally diverse polyheterocyclic compounds containing spirocyclic-fused butyrolactone-pyrrolidine-indoline and butyrolactone–pyrrolidine–dihydrobenzofuran skeletons could be smoothly obtained with excellent results (>99:1 dr and 98% ee). The potential synthetic applications of this methodology were also demonstrated by the scale-up experiment and by the diverse transformations of one product. This method is characterized by high asymmetric induction, wide functional group tolerance and scalability, and attractive product diversification.

## 1. Introduction

**Figure 1:**
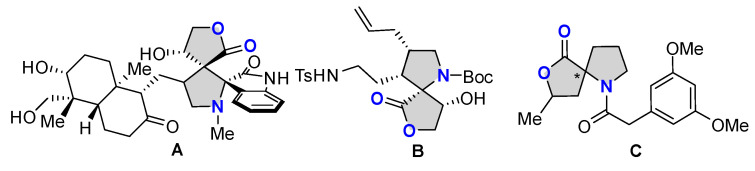
Selected bioactive compound and the key intermediate of nature products containing a chiral spirocyclic butyrolactone-pyrrolidine moiety as the structural core.

The privileged skeletons with multiple contiguous stereogenic centers are widely encountered in natural alkaloids and synthetic compounds with important biological profiles [1,2,3,4,5,6,7]. As shown in Figure 1, the dispiro–butyrolactone–pyrrolidine-fused oxindole derivative **A** exhibits significant anticancer activity [8]. Compound **B** has been used as one of the key building blocks in the total synthesis of the complex marine alkaloid (-)-Sarain A, which was reported by Overman and coworkers [9]. The spiro-heterocyclic compound **C** was used to prepare the alkaloid Cephalotaxine [10]. These bioactive molecules or key intermediates of natural products contain a chiral spirocyclic butyrolactone–pyrrolidine moiety as their structural core. In this context, the exploration of facile and efficient enantioselective approaches for the construction of optically pure spirocyclic butyrolactone–pyrrolidine compounds with innovative structures, which may be potentially applied as pharmaceutical agents, would bring some unique benefits to drug discovery and is thus particularly appealing.

**Scheme 1:**
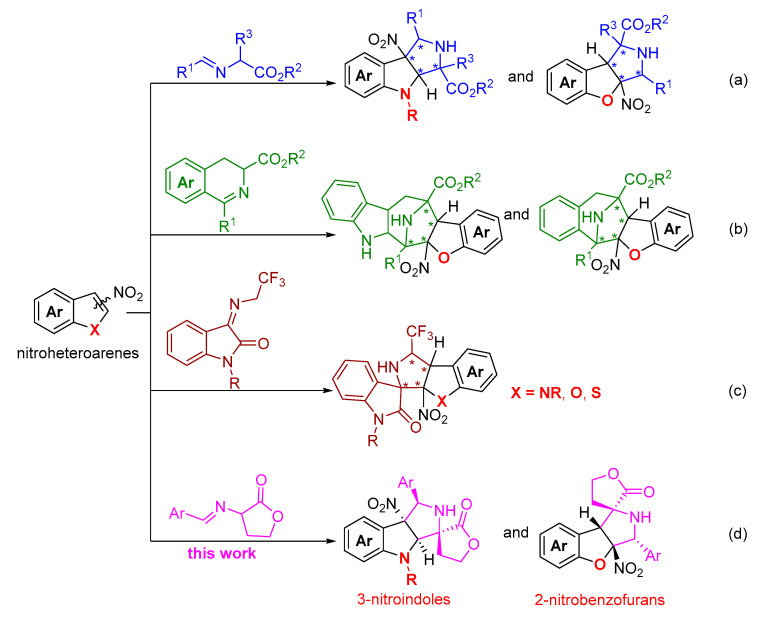
Profile of catalytic asymmetric dearomative [3+2] cycloaddition reaction of electron-deficient nitroheteroarenes and diverse azomethine ylides.

Catalytic asymmetric dearomatization reaction has emerged as one of the powerful strategies to access enantioenriched three-dimensional complex frameworks from readily available planar aromatic molecules [11,12,13,14,15,16,17]. In this aspect, considerable efforts have been devoted to exploring various catalytic asymmetric dearomatization reactions, particularly with the electron-rich heteroaromatic compounds as reactants relying on their intrinsic nucleophilic character [18,19,20]. In sharp contrast, the research with respect to electron-deficient heteroaromatic compounds remains relatively underdeveloped. Because the first palladium-catalyzed asymmetric dearomative [3+2] cycloaddition reaction of simple nitroarenes and trimethylenemethane was reported in 2014 by Trost and coworkers [21], the research on the catalytic asymmetric dearomatization reaction of electron-deficient nitroheteroarenes, including nitroindoles, nitrobenzofurans, and nitrobenzothiophenes, had attracted great interest from the synthetic organic chemistry community [22,23,24,25,26]. In this research area, the use of electron-deficient nitroheteroarenes as dipolarophiles for the asymmetric dearomative [3+2] cycloaddition reaction with various azomethine ylides has been sporadically reported. On the basis of a comprehensive and systematic literature research, it was found that only three types of azomethine ylide precursors have been explored for the asymmetric dearomative cycloaddition reactions with different electron-deficient nitroheteroarenes [27,28,29,30,31,32,33,34,35,36]. With glycine- or alanine-derived imino esters as 1,3-dipoles, the asymmetric dearomative [3+2] cycloaddition reactions of 3-nitroindoles and 2-nitrobenzofurans were realized by using different chiral copper complexes as catalysts from the groups of Arai, Stanley, and Guo, respectively (Scheme 1a) [27,28,29]. In addition, using cyclic azomethine ylides as 1,3-dipoles and 2-nitrobenzofurans as dipolarophiles, the corresponding asymmetric dearomative [3+2] cycloaddition reactions were reported by Guo and Wang, respectively (Scheme 1b) [30,31]. Beyond the two above-mentioned types of azomethine ylides, employing *N*-2,2,2-trifluoroethylisatin ketimines as a new type of highly active azomehthine ylide precursors for the catalytic enantioselective dearomative 1,3-dipolar cycloaddition reaction with diverse nitroheteroarenes was also implemented by several research groups (Scheme 1c) [32,33,34,35,36]. Despite these successful precedents, considering the great application potential and the promising prospect of polyheterocyclic compounds with structural diversity in drug discovery programs, exploiting new types of azomethine ylides which enable the enantioselective dearomative cycloaddition reaction of diverse electron-deficient nitroheteroarenes for accessing elaborated heterocycles with creative frameworks is still in high demand.

*α*-Imino *γ*-lactones as azomethine ylide precursors have proven to be a type of efficient 1,3-dipole that could be used in catalytic enantioselective [3+2] cycloadditon reactions with various dipolarophiles for the construction of spirocyclic butyrolactone–pyrrolidine skeletons [37,38,39,40]. Enlightened by the previous works on the asymmetric cycloaddition reaction with *α*-imino *γ*-lactones as 1,3-dipoles, we speculated that the asymmetric dearomative cycloaddition reaction of *α*-imino *γ*-lactones and electron-deficient nitroheteroarenes would take place in the presence of a suitable chiral copper complex as the catalyst. As a continuation of our research interest in developing catalytic asymmetric dearomatization reactions of electron-deficient nitroheteroarenes [41,42,43,44,45,46,47], we recently developed an efficient enantioselective dearomative [3+2] cycloaddition of *α*-imino *γ*-lactones and 3-nitroindoles with a chiral copper complex as the catalyst, providing a facile access to spirocyclic butyrolactone–pyrrolidine–indoline derivatives in up to 67% yield for the major diastereomer with 80:20:0:0 dr and 98% ee (Scheme 1d). Moreover, the similar catalytic system was also extended to the dearomative [3+2] cycloaddition reaction of 2-nitrobenzofurans for the construction of spirocyclic butyrolactone–pyrrolidine–dihydrobenzofuran compounds in up to 86% yield for the major diastereomer with >99:1 dr and 98% ee (Scheme 1d). Herein, we wish to report the details of our study on this subject.

## 2. Results and Discussion

### 2.1. Optimization Studies

Initially, we investigated the catalytic asymmetric dearomative [3+2] cycloaddition reaction of 3-nitroindole **1a** and *α*-imino *γ*-lactone **2a** with Cs_2_CO_3_ as the base and methyl *tert*-butyl ether (MTBE) as the solvent for screening the optimal reaction conditions. As shown in Table 1, with the complex of Cu(CH_3_CN)_4_PF_6_ and (*S*)-BINAP-**L1** as the catalyst, the reaction of **1a** and **2a** smoothly proceeded, giving the desired product **3a** in 59% yield for the major diastereomer with moderate diastereoselectivity and 60% ee value (Table 1, entry 1). When the Pybox-**L2** or (*S*)-*^i^*Pr-PHOX-**L3** as the chiral ligand for the reaction was examined, product **3a** was obtained with only 42% ee and 35% ee, respectively (Table 1, entries 2 and 3). We also screened other BINAP derivatives as ligands, but no improved results were observed [48]. In the presence of (*S*)-BINAP-**L1**, replacing the metal salt Cu(CH_3_CN)_4_PF_6_ with CuBr, Cu(OAc)_2_, or Cu(OTf)_2_, we found that the reaction with Cu(OTf)_2_ was able to give product **3a** with the best results (52% yield for the major diastereomer, 57:12:31:0 dr, and 81% ee) (Table 1, entry 6) under the identical conditions (Table 1, entries 4–6). Afterward, a survey of solvents including THF, CH_2_Cl_2_, toluene, PhCl, and xylene was implemented, and it indicated PhCl was the best candidate to generate product **3a** in 45% yield for the major diastereomer, with 55:16:29:0 dr and 85% ee (Table 1, entry 10 vs. entries 7–9 and 11). Then, with anhydrous MgSO_4_ as an additive, a slightly improved enantioselectivity to 89% ee could be observed (Table 1, entry 12). Running the reaction at 0 °C, **3a** could be obtained in 49% yield for the major diastereomer with 91% ee value but a prolonged reaction time to 120 h was needed (Table 1, entry 13). Ultimately, we were delighted to find that performing the reaction in PhCl with anhydrous MgSO_4_ as an additive at −30 °C for 120 h, the reaction proceeded well to provide **3a** in 61% yield for the major diastereomer with good diastereoselectivity and up to 98% ee (Table 1, entry 14).

### 2.2. Substrate Scope Studies

**Scheme 2:**
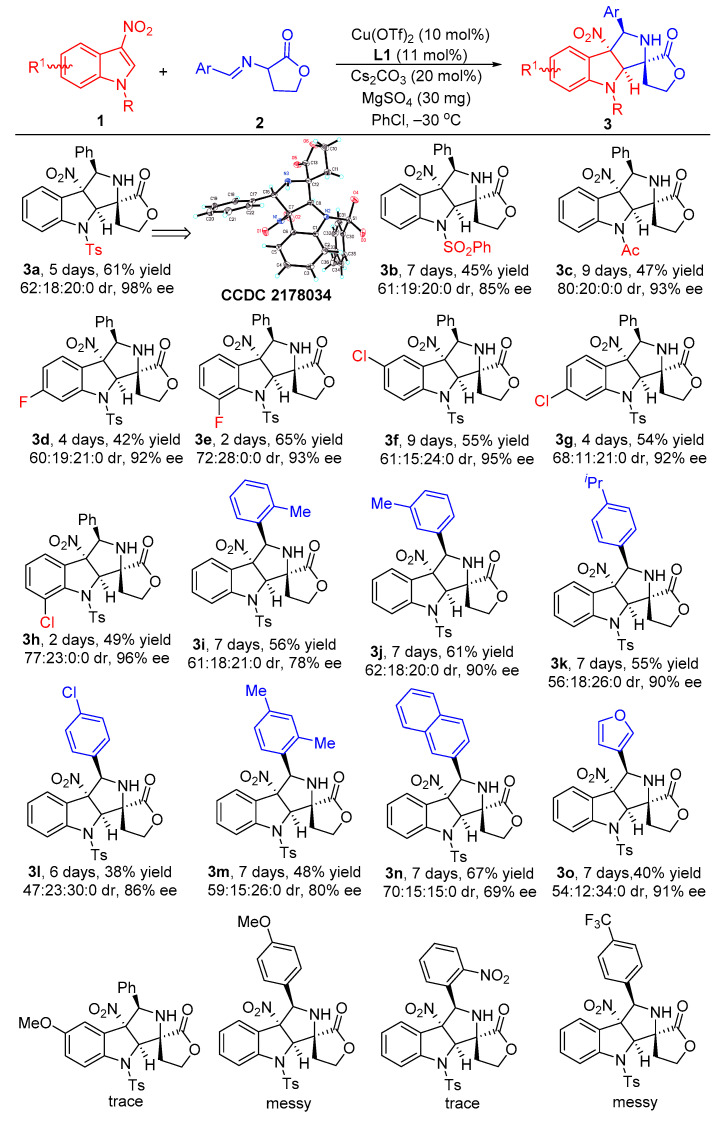
Substrate scope investigation of the catalytic asymmetric dearomtive [3+2] cycloaddition reaction of 3-nitroindoles and *α*-imino *γ*-lactones. Reaction conditions: the reactions were carried out with 3-nitroindoles **1** (0.2 mmol, 1.0 equiv), *α*-imino *γ*-lactones **2** (0.3 mmol, 1.5 equiv), Cu(OTf)_2_ (10 mol%), **L1** (11 mol%), Cs_2_CO_3_ (20 mol%), and MgSO_4_ (30 mg) in 2.0 mL of PhCl as the solvent at −30 °C under argon atmosphere. The yields refer to the isolated yield of the major diastereomers. The dr values were determined by ^1^H NMR spectroscopy of the crude mixture. The ee values were determined by chiral high-performance liquid chromatography (HPLC) analysis after purification of the diastereomers.

With the optimized reaction conditions in hand, we set out to investigate the substrate scope and generality of the copper-catalyzed asymmetric dearomative [3+2] cycloaddition reaction of different 3-nitroindoles **1** and diverse *α*-imino *γ*-lactones **2**. As shown in Scheme 2, the 3-nitroindoles bearing different *N*-protecting groups, such as Ts-, Bs- and Ac-, were suitable substrates in reaction with *α*-imino *γ*-lactone **2a** for generating the corresponding products **3a–c** in 45–61% yields with 85–98% ee for their major diastereomers. In addition, installing different electron-withdrawing groups into the aromatic ring of *N*-Ts-3-nitroindoles, regardless of their positions on the aromatic ring, was not detrimental to reactivities and stereoselectivities of the dearomative [3+2] cycloaddition, affording major diastereomers products **3d**–**h** in 42–65% yields with acceptable diastereoselectivities and excellent ee values (92–96% ee). However, for the reaction between 5-OMe substituted 3-nitroindole with α-imino γ-lactone **2a**, only a trace amount of product could be observed. On the other hand, the investigation of the *α*-imino *γ*-lactones was also conducted by using the standard reaction conditions. Both electron-donating and electron-withdrawing substituents at different positions on the phenyl ring of *α*-imino *γ*-lactones were well tolerated in reaction with 3-nitroindole **1a**, providing the desired cycloaddition products **3i**–**l** in 38–61% yields with good diastereoselectivities and 78–90% ee for their major diastereomers. Moreover, the disubstituted substrate also could smoothly react with 3-nitroindole **1a** to give the corresponding product **3m** in 48% yield for the major diastereomer with 59:15:26:0 dr and 80% ee. As for bulker naphthyl-substituted *α*-imino *γ*-lactone, the developed catalytic system also showed good compatibility, furnishing the product **3n** in 67% yield with good stereoselectivity. Additionally, the heteroaromatic substrate, such as 3-fury-substituted *α*-imino *γ*-lactone, also efficiently worked in this asymmetric dearomative [3+2] cycloaddition reaction, providing the desired product **3o** in 40% yield for the major diastereomer with 54:12:34:0 dr and 91% ee. To our disappointment, the *α*-imino *γ*-lactones **2** with strong EDG (-OMe) or EWG groups (–NO_2_ and –CF_3_) in the phenyl ring by reacting with 3-nitroindole **1** became messy or showed very poor reactivity, and no expected products could be obtained. The absolute and relative configuration of product **3a** was unambiguously determined to be C7*R*, C8*R*, C12*S*, and C16*R* by single crystal X-ray analysis, the configurations of all other products in Scheme 2 were assigned by analogy due to their formation via a common reaction pathway [49].

**Scheme 3:**
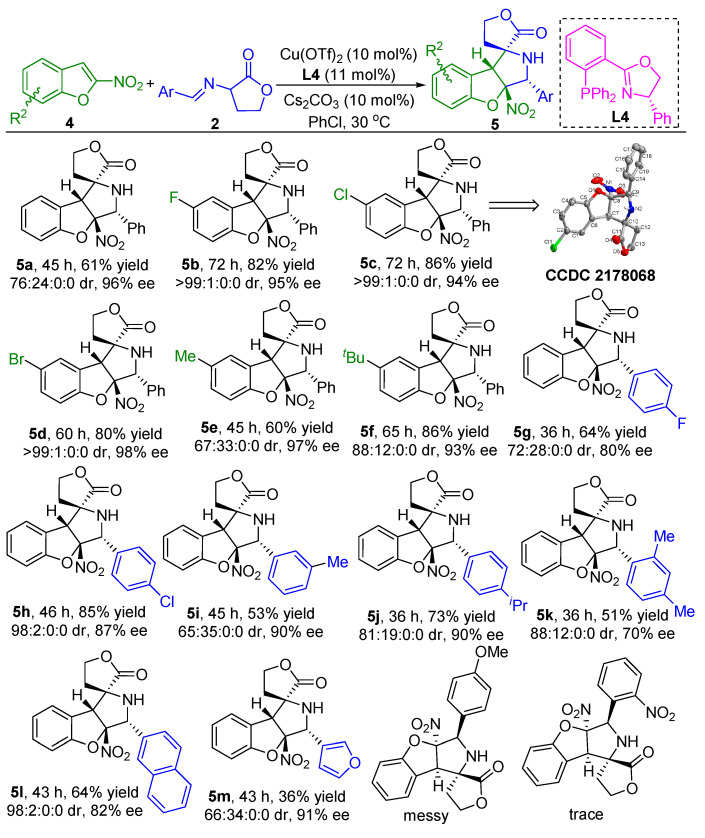
Substrate scope investigation of the catalytic asymmetric dearomtive [3+2] cycloaddition reaction of 2-nitrobenzofurans and *α*-imino *γ*-lactones. Reaction conditions: the reactions were carried out with 2-nitrobenzofurans **4** (0.2 mmol, 1.0 equiv), *α*-imino *γ*-lactones **2** (0.3 mmol, 1.5 equiv), Cu(OTf)_2_ (10 mol%), **L4** (11 mol%), and Cs_2_CO_3_ (10 mol%) in 2.0 mL of PhCl as the solvent at 30 °C under argon atmosphere. The yields refer to the isolated yield of the major diastereomers. The dr values and ee values were determined by chiral high-performance liquid chromatography (HPLC) analysis.

Having established a general scope to the catalytic asymmetric dearomative [3+2] cycloaddition reaction between 3-nitroindoles **1** and *α*-imino *γ*-lactones **2**, we next attempted to apply the similar catalytic system to the asymmetric dearomative [3+2] cycloaddition reaction between *α*-imino *γ*-lactones **2** and 2-nitrobenzofuran substrates **4** (for details of the optimization of conditions, see the Supporting Information). As illustrated in Scheme 3, with the complex of Cu(OTf)_2_ and (*S*)-Ph-PHOX ligand **L4** as the catalyst, Cs_2_CO_3_ as the base, the asymmetric dearomative [3+2] cycloaddition reaction of 2-nitrobenzofuran **4a** and *α*-imino *γ*-lactone **2a** smoothly proceeded in PhCl at 30 °C, furnishing the desired product **5a** in 61% yield for the major diastereomer with 76:24:0:0 dr and 96% ee. As for the 2-nitrobenzofurans bearing various electron-withdrawing groups such as F-, Cl-, and Br- on the aryl ring, these substrates could work well in reaction with *α*-imino *γ*-lactone **2a** under the standard conditions to give products **5b**–**d** in 80–86% yields with excellent stereoselectivities (>99:1:0:0 dr and 94–98% ee). Likewise, 2-nitrobenzofurans bearing an electron-donating group were also viable for the developed protocol, as demonstrated in the synthesis of products **5e** and **5f** in good yields with 97% ee and 93% ee, respectively. On the other hand, the asymmetric dearomative [3+2] cycloaddition reactions of diverse *α*-imino *γ*-lactones containing either electron-withdrawing or electron-donating groups on the aryl ring with 2-nitrobenzofuran **4a** could smoothly occur under the standard conditions, delivering the corresponding dearomative cycloaddition products **5g**–**j** in 53–85% yields with good stereoselectivities for their major diastereomers (up to 98:2:0:0 dr and 90% ee). In addition, the *α*-imino *γ*-lactone with a double substitution pattern could also smoothly react with 2-nitrobenzofuran **4a** under the standard conditions to generate product **5k** in 51% yield for the major diastereomer with 88:12:0:0 and 70% ee. The bulky naphthyl group was also tolerated well with the catalytic system, resulting in the formation of product **5l** in moderate yield with excellent diastereoselectivity and good enantioselectivity. Moreover, the 3-fury substituted substrate could smoothly react with 2-nitrobenzofuran **4a**, delivering the corresponding product **5m** in moderate yield with 66:34:0:0 dr and 91% ee. However, when *para*-OMe- or *ortho*-NO_2_-substituted *α*-imino *γ*-lactone was tested, no expected products could be obtained. The absolute and relative configuration of product **5c** was determined to be C5*R*, C6*R*, C7*R*, and C8*R* by single crystal X-ray analysis, the configurations of all other products in Scheme 3 were assigned by analogy due to their formation via a common reaction pathway [49].

### 2.3. Scale-Up Experiment and the Versatile Transformations of Product ***3a***

**Scheme 4:**
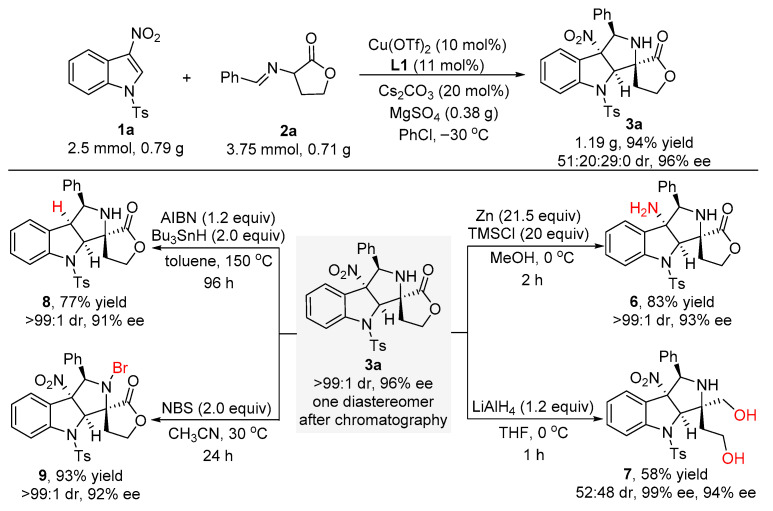
Scale-up experiment and further transformations of compound **3a**.

In order to demonstrate the potential synthetic application of the developed catalytic asymmetric dearomative [3+2] cycloaddition reaction, a scale-up experiment between 3-nitroindole **1a** and *α*-imino *γ*-lactone **2a** was conducted under the standard conditions. As shown in Scheme 4, the gram scale reaction was able to proceed to completion at −30 °C after 120 h, smoothly affording the cycloaddition product **3a** in 94% yield for the sum of the diastereomers with 51:20:29:0 dr and 96% ee. To our delight, the major diastereomer of product **3a** could be easily isolated by flash column chromatography on silica gel (0.6 g, 48% yield) without any loss of the enantioselectivity (>99:1 dr and 96% ee). Moreover, different synthetic transformations of **3a** to other heterocyclic compounds were also performed (Scheme 4). Treating **3a** with newly activated zinc powder and TMSCl in MeOH solvent for two hours, the nitro group of **3a** could be converted into an amine group to give compound **6** in 83% yield with >99:1 dr and 93% ee. Reduction of **3a** with LiAlH_4_ in THF for one hour gave rise to the formation of compound **7** in 58% yield with 52:48 dr and excellent enantioselectivities (99% ee and 94% ee for the diastereomers, respectively). In addition, **3a** was subjected to tributyltin hydrogen and AIBN in toluene at 150 °C for 96 h in a sealed tube, thus affording compound **8** in 77% yield with >99:1 dr and 91% ee via a radical denitration process. Ultimately, the bromination reaction of **3a** with *N*-bromosuccinimide (NBS) in CH_3_CN solvent could smoothly occur at 30 °C to produce compound **9** in 93% yield with >99:1 dr and 92% ee. 

### 2.4. Proposed Mechanism for the Catalytic Asymmetric Dearomative [3+2] Cycloaddition

**Scheme 5:**
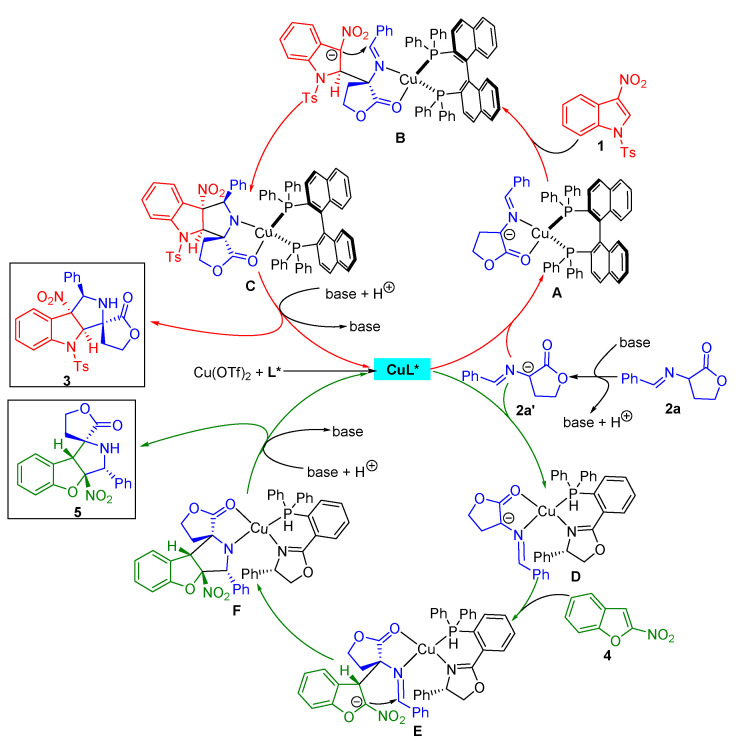
Proposed mechanism for the catalytic asymmetric dearomative [3+2] cycloaddition of *α*-imino *γ*-lactones with 3-nitroindoles and 2-nitrobenzofurans.

Based on our experimental results and previous related reports [27,28,29,30,31,37,38,39,40], a plausible catalytic mechanism involving a stepwise reaction process was assumed to explain the stereoselectivity of the asymmetric dearomative [3+2] cycloaddition of *α*-imino *γ*-lactones with electron-deficient nitroheteroarenes (Scheme 5). For the reaction with 3-nitroindoles as partners, the in situ-formed azomethine ylide **2a’** is coordinated to the chiral CuL* complex, leading to the active species **A**. Under the chiral environmental control, the *α*-carbon anion of **2a’** approaches the *Re* face of the C2-position of 3-nitroindoles **1,** resulting in the intermediate **B**. Subsequently, the C3-positon carbon anion of **1** attacks the *Si* face of the C=N of azomethine ylide to deliver the species **C**, which is protonated, giving rise to the formation of products **3** with the specific stereochemical outcome and regenerating the chiral catalyst for the next cycle. Likewise, for the reaction with 2-nitrobenzofurans as partners, the active species **D** is formed from the azomethine ylide **2a’** and the chiral CuL* complex. The *α*-carbon anion of **2a’** attacks the *Si* face of the C3-position of 2-nitrobenzofurans to form intermediate **E**, which undergoes an intramolecular cyclization from the C2-positon carbon anion of **4** to the *Si* face of the C=N of azomethine ylide to generate species **F**. Then, a protonation of **F** leads to the formation of products **5** and the release of a chiral catalyst. 

## 3. Materials and Methods

### 3.1. General Information

Reagents were purchased from commercial sources and were used as received unless mentioned otherwise. Reactions were monitored by thin-layer chromatography (TLC). ^1^H NMR and ^13^C NMR spectra were recorded in CDCl_3_ and DMSO-*d*_6_. ^1^H NMR chemical shifts are reported in ppm relative to tetramethylsilane (TMS) with the solvent resonance employed as the internal standard (CDCl_3_ at 7.26 ppm and DMSO-*d*_6_ at 2.50 ppm). Data are reported as follows: chemical shift, multiplicity (s = singlet, br s = broad singlet, d = doublet, t = triplet, q = quartet, m = multiplet), coupling constants (Hz), and integration. ^13^C NMR chemical shifts are reported in ppm from tetramethylsilane (TMS) with the solvent resonance as the internal standard (CDCl_3_ at 77.20 ppm and DMSO-*d*_6_ at 39.52 ppm). The enantiomeric excesses were determined by chiral high-performance liquid chromatography (HPLC) analysis. High-performance liquid chromatography analysis (HPLC) was performed on Shimadzu SCL-10AVP HPLC systems consisting of a LC-10AD pump and an SPD-10A detector, measured at 254 nm. High-resolution mass spectrometer (HRMS) was recorded on Bruker Q TOF. Optical rotations were measured with a Perkin-Elmer-341 polarimeter. Melting points were recorded on a Büchi Melting Point B-545.

### 3.2. General Experimental Procedure for the Catalytic Asymmetric Dearomative [3+2] Cycloaddtion Reaction of α-imino γ-lactones with 3-nitroindoles for the Synthesis of Products ***3*** (Scheme 2)

MgSO_4_ (30 mg), ligand **L1** (11 mol%, 0.11 equiv), and Cu(OTf)_2_ (10 mol%, 0.1 equiv) were added to a Schlenk tube containing a magnetic stir bar under an argon atmosphere. chlorobenzene (1.0 mL) was added to the tube and the mixture was stirred for 1.0 h at room temperature. Then, in the resulting solution, a mixture including *α*-imino *γ*-lactones (0.3 mmol, 1.5 equiv), Cs_2_CO_3_ (20 mol%, 0.20 equiv), 3-nitroindoles (0.2 mmol, 1.0 equiv), and chlorobenzene (1.0 mL) was subsequently added under an argon atmosphere at −30 °C. After being stirred for the appropriate reaction time, the reaction mixture was quenched with water. The organic layer was extracted with dichloromethane three times. The collected organic layer was dried over anhydrous Na_2_SO_4_. After the removal of the solvent under reduced pressure, the diastereomeric ratio was determined by ^1^H NMR analysis. The resulting crude mixture was purified by silica gel column chromatography to afford cycloadducts **3**. The enantiomeric excesses of product **3** were determined by chiral stationary phase HPLC using a Chiralpak AD-H, AS-H, IA, IB, and Chiralpak IC column. 

### 3.3. General Experimental Procedure for the Catalytic Asymmetric Dearomative [3+2] Cycloaddtion Reaction of α-imino γ-lactones with 2-nitrobenzofurans for the Synthesis of Products ***5*** (Scheme 3)

Ligand **L4** (11 mol%, 0.11 equiv) and Cu(OTf)_2_ (10 mol%, 0.10 equiv) were added to a Schlenk tube containing a magnetic stir bar under an argon atmosphere. Chlorobenzene (1.0 mL) was added to the tube and the mixture was stirred for 1.0 h at room temperature. Then, in the resulting solution, a mixture including *α*-imino *γ*-lactones (0.3 mmol, 1.5 equiv), Cs_2_CO_3_ (10 mol%, 0.10 equiv), 2-nitrobenzofurans (0.2 mmol, 1.0 equiv), and chlorobenzene (1.0 mL) were subsequently added under an argon atmosphere at 30 °C. After being stirred for the appropriate reaction time, the reaction mixture was quenched with water. The organic layer was extracted three times with dichloromethane. The collected organic layer was dried over anhydrous Na_2_SO_4_. After removal of the solvent under reduced pressure, the resulting crude mixture was purified by silica gel column chromatography to afford cycloadducts **5**. The diastereomeric ratio and the enantiomeric excess values of product **5** were determined by chiral stationary phase HPLC using a Chiralpak AD-H, AS-H, IA, IB, and Chiralpak IC column.

### 3.4. Procedure for the Catalytic Asymmetric Synthesis of Product 3a on a 2.5 mmol Scale

MgSO_4_ (375 mg), ligand **L1** (11 mol%, 171 mg), and Cu(OTf)_2_ (10 mol%. 90.5 mg) were added to a Schlenk tube containing a magnetic stir bar under an argon atmosphere. chlorobenzene (12.5 mL) was added to the tube and the mixture was stirred for 1.0 h at room temperature. In the resulting solution, the mixture including *α*-imino *γ*-lactone **2a** (3.75 mmol, 0.71 g), Cs_2_CO_3_ (20 mol%, 163 mg), 3-nitroindole **1a** (2.5 mmol, 0.79 g), and chlorobenzene (12.5 mL) were subsequently added under an argon atmosphere at −30 °C. After being stirred for 120 h, the reaction mixture was quenched with water. The organic layer was extracted three times with dichloromethane. The collected organic layer was dried over anhydrous Na_2_SO_4_. After the removal of the solvent under reduced pressure, the diastereomeric ratio was determined as 51:20:29:0 dr by ^1^H NMR analysis. The resulting crude mixture was purified by silica gel column chromatography using petroleum ether/ethyl acetate (8:1) as the eluent to afford cycloadduct **3a** for the sum of diastereomers (1.19 g, 94% yield, 51:20:29:0 dr, and 96% ee).

### 3.5. Procedure for the Synthesis of Compound ***6***

Newly activated zinc powder (140.6 mg, 2.15 mmol, 21.5 equiv) was slowly added into a solution of **3a** (>99:1 dr, 96% ee) (50.6 mg, 0.10 mmol, 1.0 equiv) and trimethylsilyl chloride (0.25 mL, 2.00 mmol, 20.0 equiv) in 1.0 mL of methanol at 0 °C. After the mixture was stirred at 0 °C for 2 h, the suspension was filtered and washed with methanol two times (2 × 5.0 mL) and dichloromethane two times (2 × 5.0 mL), respectively. After the filtrate was washed with saturated NaHCO_3_ aqueous two times (2 × 15 mL), the organic phase was separated while the aqueous phase was extracted with dichloromethane three times (3 × 15 mL). The combined organic phase was washed with brine three times (3 × 15 mL) and then dried with anhydrous Na_2_SO_4_. Subsequently, the organic phase was concentrated under reduced pressure. The residue was subjected to column chromatography over silica gel using petroleum ether/ethyl acetate (4:1) as the eluent to give compound **6** as a white solid (83% yield, >99:1 dr, and 93% ee).

### 3.6. Procedure for the Synthesis of Compound ***7***

LiAlH_4_ (4.5 mg, 0.12 mmol, 1.2 equiv) was cautiously added into the solution of compound **3a** (>99:1 dr, 96% ee) (50.6 mg, 0.1 mmol, 1.0 equiv) in the 1.0 mL of THF at 0 °C. After the mixture was stirred at 0 °C for 1.0 h, the resulting mixture was quenched with 10 mL of 0.1 mol/L NaOH solution and extracted with dichloromethane three times (3 × 15 mL). The organic phase was isolated and washed with brine three times (3 × 15 mL), and then dried with anhydrous Na_2_SO_4_. Subsequently, the organic phase was concentrated under reduced pressure. The residue was subjected to column chromatography over silica gel using petroleum ether/ethyl acetate (2:1) (about 200 mL) as eluent, and then using MeOH as an eluent to give compound **7** as a white solid (58% yield, 52:48 dr, and 99% ee/94% ee).

### 3.7. Procedure for the Synthesis of Compound ***8***

Tributyltin hydride (54 µL, 58.2 mg, 0.2 mmol, 2.0 equiv) was added to a solution of compound **3a** (>99:1 dr, 96% ee) (50.6 mg, 0.1mol, 1.0 equiv) and AIBN (azobisisobutyronitrile) (19.7 mg, 0.12 µmol, 1.2 equiv) in toluene (1.0 mL). Then, the reaction mixture in a sealed tube was stirred at 150 °C for 96 h. Subsequently, the reaction mixture was cooled to room temperature, the solution was concentrated under reduced pressure. The residue was subjected to column chromatography over silica gel using petroleum ether/ethyl acetate (7:1) as an eluent to give compound **8** as a white solid (77% yield, >99:1 dr, and 91% ee).

### 3.8. Procedure for the Synthesis of Compound ***9***

NBS (*N*-Bromosuccinimide) (35.6 mg, 0.2 mmol, 2.0 equiv) was added into the solution of compound **3a** (>99:1 dr, 96% ee) (50.6 mg, 0.1 mmol, 1.0 equiv) in the 1.0 mL of MeCN, and the reaction mixture was stirred at 30 °C for 24 h. Subsequently, the resulting mixture was quenched with 10 mL of saturated NH_4_Cl solution and extracted with dichloromethane three times (3 × 15 mL). The combined organic phase was washed with brine three times (3 × 15 mL) and then dried with anhydrous Na_2_SO_4_. Subsequently, the organic phase was concentrated under reduced pressure. The residue was subjected to column chromatography over silica gel using petroleum ether/ethyl acetate (6:1) as an eluent to give compound **9** as a white solid (93% yield, >99:1 dr, and 92% ee).

## 4. Conclusions

In summary, we have successfully developed a copper-catalyzed asymmetric dearomative [3+2] cycloaddition reaction of *α*-imino *γ*-lactones with nitroheteroarenes including 3-nitroindoles and 2-nitrobenzofurans. A wide range of structurally diverse polyheterocyclic compounds containing spirocyclic-fused butyrolactone–pyrrolidine–indoline and butyrolactone–pyrrolidine–dihydrobenzofuran skeletons bearing four contiguous chiral stereocenters could be smoothly obtained with excellent results (>99:1 dr and 98% ee) under mild reaction conditions. Importantly, these innovative polyheterocyclic compounds would be potentially promising for high-throughout drug screening due to the intriguing fusion of three privileged butyrolactone, pyrrolidine, and indoline/dihydrobenzofuran subunits into one molecular architecture. The potential synthetic applications of the dearomative [3+2] cycloaddition reaction were also demonstrated by the scale-up experiment and the diverse transformations of one product to diverse heterocyclic compounds. More importantly, this developed protocol also shows distinct features including high asymmetric induction, broad functional group tolerance and scalability, and attractive product diversification. The application of the asymmetric dearomative cycloaddition reaction of electron-deficient nitroheteroarenes toward library synthesis and subsequent biological evaluation of its members is underway.

## Figures and Tables

**Table 1:**
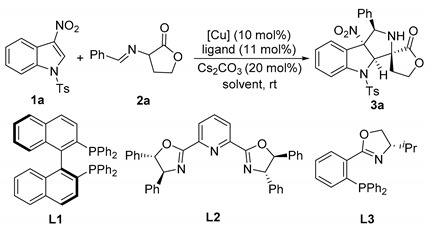
Optimization of reaction conditions [a].

Entry	[Cu]	Ligand	Solvent	Time (h)	Yield (%) [b]	dr [c]	ee (%) [d]
1	Cu(CH_3_CN)_4_PF_6_	**L1**	MTBE	72	94 (59)	63:15:22:0	60
2	Cu(CH_3_CN)_4_PF_6_	**L2**	MTBE	72	96 (64)	67:20:13:0	42
3	Cu(CH_3_CN)_4_PF_6_	**L3**	MTBE	66	93 (47)	50:15:35:0	35
4	CuBr	**L1**	MTBE	70	85 (54)	63:11:26:0	21
5	Cu(OAc)_2_	**L1**	MTBE	70	72 (47)	65:10:24:0	31
6	Cu(OTf)_2_	**L1**	MTBE	70	91 (52)	57:12:31:0	81
7	Cu(OTf)_2_	**L1**	THF	68	87 (60)	69:9:22:0	17
8	Cu(OTf)_2_	**L1**	CH_2_Cl_2_	68	86 (52)	61:11:28:0	46
9	Cu(OTf)_2_	**L1**	toluene	68	92 (46)	50:15:35:0	78
10	Cu(OTf)_2_	**L1**	PhCl	68	82 (45)	55:16:29:0	85
11 [e]	Cu(OTf)_2_	**L1**	PhCl	90	99 (51)	52:16:32:0	89
12 [e,f]	Cu(OTf)_2_	**L1**	PhCl	120	99 (49)	49:18:33:0	91
13 [e,g]	Cu(OTf)_2_	**L1**	PhCl	120	99 (61)	62:18:20:0	98

[a] Unless otherwise noted, the reaction was carried out with **1a** (0.2 mmol, 1.0 equiv), **2a** (0.3 mmol, 1.5 equiv), Cu salt (10 mol%), ligand (11 mol%), and Cs_2_CO_3_ (20 mol%) in 2.0 mL of solvent at room temperature under argon atmosphere. [b] Isolated yield of the sum of the diastereomers. The yield in the bracket refers to the isolated yield of the major diastereomer. [c] The dr values were determined by ^1^H NMR. [d] The ee values were determined by chiral high-performance liquid chromatography (HPLC) analysis after purification of the diastereomers. [e] 30 mg MgSO_4_ was added to the reaction mixture as an additive. [f] The reaction was run at 0 °C. [g] The reaction was run at −30 °C.

## Data Availability

All the required data are reported in the manuscript and Supplementary Materials.

## Supplementary Materials

The following supporting information can be downloaded at: https://www.mdpi.com/article/10.3390/molecules28062765/s1, characterization data for obtained products; X-ray data for products **3a** and **5c**; copies of ^1^H, ^13^C NMR, and HPLC spectra.

## Sample Availability

Samples of the compounds are available from the authors.
