# Supplementary material for: Copper-Catalyzed Asymmetric Dearomative [3+2] Cycloaddition of Nitroheteroarenes with Azomethines
Source: Molecules. 2023 Mar 19;28(6):2765. doi: 10.3390/molecules28062765 (PMC10057014; doi:10.3390/molecules28062765)
Supplement: Supplementary file 1 [file molecules-28-02765-s001.zip › molecules-2230719-supplementary.pdf]

Copper-Catalyzed Asymmetric Dearomative [3+2] Cycloaddition of Nitroheteroarenes with Azomethines

Yan Chen[1,2,3], Jian-Qiang Zhao[2], Yan-Ping Zhang[2], Ming-Qiang Zhou[1,2,3], Xiao-Mei Zhang[1,4,*] and Wei-Cheng Yuan[1,2,*]

1 National Engineering Research Center of Chiral Drugs, Chengdu Institute of Organic Chemistry, Chinese Academy of Sciences, Chengdu 610041

2 Innovation Research Center of Chiral Drugs, Institute for Advanced Study, Chengdu University, Chengdu 610106, China

3 University of Chinese Academy of Sciences, Beijing, 100049, China

4 Department of Chemistry, Xihua University, Chengdu, 610039, China

E-mail: xmzhang@cioc.ac.cn; yuanwc@cioc.ac.cn

Supporting Information

Table of Contents

1. Characterization data for compounds **3**…………………..S1

2. Optimization of conditions for asymmetric synthesis of **5**...S8

3. Characterization data for compounds **5**…………………..S9

4. Characterization data for compounds **6-9**..……...........................…………….................S14

5. X-ray crystal structure of compound **3a** and **5c**…………………………………..…...…....…S16

6. Unsuccessful examples.……...........................……………….................………..…...…...…S18

6. ^{1}H, ^{13}C NMR and HPLC spectra for compounds **3,5** and **6-9**...S20

1. Characterization data for compounds 3

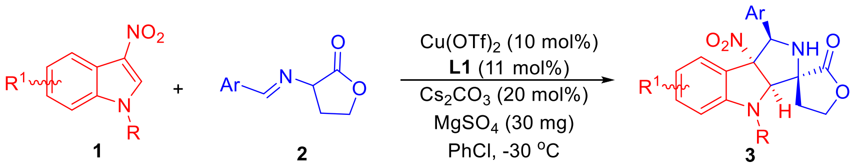

(1'*R*,3*S*,3a'*R*,8b'*R*)-8b'-nitro-1'-phenyl-4'-tosyl-1',3a',4,4',5,8b'-hexahydro-2*H*,2'*H*-spiro[furan-3,3'-pyrrolo[3,4-*b*]indol]-2-one (3a)

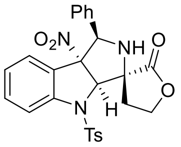

100 mg, 99% yield for the sum of the diastereomers, 61% yield for the major isomer. white soild, 62:18:20:0 dr, 98% ee, mp 182.5-183.1 ℃; **The ee was determined by HPLC** (Chiralpak AD-H, EtOH/hexane = 20/80, flow rate 1.0 mL/min, λ = 254 nm, major diastereomer: t_{minor} = 11.6 min, t_{major} = 24.4 min),; $[\alpha]_D^{20}$ = +104.2 (*c* 1.00, CH_2Cl_2) for major diastereomer;

^{1}H NMR (400 MHz, DMSO-*d*$_6$) δ (major diastereomer) 7.69 (d, *J* = 8.2 Hz, 1H), 7.52 – 7.44 (m, 3H), 7.40 – 7.33 (m, 1H), 7.28 (dd, *J* = 13.4, 7.9 Hz, 4H), 7.06 (d, *J* = 7.5 Hz, 2H), 6.86 – 6.80 (m, 1H), 5.78 (d, *J* = 8.1 Hz, 1H), 5.51 (s, 1H), 5.33 (d, *J* = 5.8 Hz, 1H), 4.65 (d, *J* = 5.8 Hz, 1H), 4.59 (td, *J* = 8.5, 3.9 Hz, 1H), 4.45 (m, *J* = 8.3 Hz, 1H), 3.04 (dt, *J* = 13.6, 8.2 Hz, 1H), 2.36 – 2.29 (m, 1H), 2.27 (s, 3H).

^{13}C NMR (101 MHz, DMSO-*d*$_6$) δ (major diastereomer) 177.1, 145.4, 143.5, 134.7, 132.1, 131.8, 130.0, 129.0, 128.6, 127.8, 127.6, 127.5, 124.34, 124.29, 116.2, 101.1, 73.9, 66.9, 66.6, 66.5, 31.6, 21.0.

HRMS (ESI-TOF) *m/z*: $[M + H]^+$ calcd for $C_{26}H_{24}N_3O_6S$, 506.1380; found, 506.1383.

(1'*R*,3*S*,3a'*R*,8b'*R*)-8b'-nitro-1'-phenyl-4'-(phenylsulfonyl)-1',3a',4,4',5,8b'-hexahydro-2*H*,2'*H*-spiro[furan-3,3'-pyrrolo[3,4-*b*]indol]-2-one (3b)

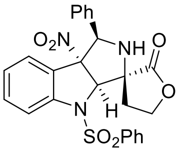

71.5 mg, 73% yield for the sum of the diastereomers, 45% yield for the major isomer, white soild, 61:19:20:0 dr;85% ee, mp 103.9-104.6 ℃; **The ee was determined by HPLC** (Chiralpak AS-H, EtOH/hexane = 10/90, flow rate 1.0 mL/min, λ = 220 nm, major diastereomer: t_{minor} = 14.1 min, t_{major} = 28.9 min); $[\alpha]_D^{20}$ = +36.3 (*c* 1.00, CH_2Cl_2) for major diastereomer;

^{1}H NMR (400 MHz, DMSO-*d*$_6$) δ (major diastereomer) δ 7.72 (d, *J* = 8.2 Hz, 1H), 7.65 – 7.60 (m, 3H), 7.50 – 7.44 (m, 3H), 7.37 (d, *J* = 7.2 Hz, 1H), 7.32 – 7.27 (m, 2H), 7.06 (d, *J* = 7.5 Hz, 2H), 6.88 – 6.83 (m, 1H), 5.79 (d, *J* = 6.6 Hz, 1H), 5.50 (s, 1H), 5.32 (d, *J* = 5.4 Hz, 1H), 4.67 (d, *J* = 6.2 Hz, 1H), 4.60 (td, *J* = 8.5, 3.9 Hz, 1H), 4.46 (td, *J* = 8.5, 6.8 Hz, 1H), 3.05 (dt, *J* = 13.6, 8.3 Hz, 1H), 2.32 (ddd, *J* = 13.6, 6.8, 3.8 Hz, 1H).

^{13}C NMR (101 MHz, DMSO-*d*$_6$) δ (major diastereomer) 177.0, 143.4, 134.7, 134.61, 134.57, 132.2, 129.6, 129.1, 128.6, 127.8, 127.7, 127.4, 124.5, 124.3, 116.3, 101.0, 73.8, 66.9, 66.6, 66.5, 31.7. **HRMS** (ESI-TOF) *m/z*: $[M + H]^+$ calcd for $C_{25}H_{22}N_3O_6S$, 492.1224; found, 492.1226.

(1'*R*,3*S*,3a'*R*,8b'*R*)-4'-acetyl-8b'-nitro-1'-phenyl-1',3a',4,4',5,8b'-hexahydro-2*H*,2'*H*-spiro[furan-3,3'-pyrrolo[3,4-*b*]indol]-2-one (3c)

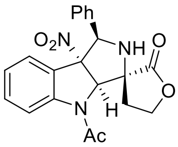

46.4 mg, 59% yield for the sum of the diastereomers, 47% yield for the major isomer, white soild, 80:20:0:0 dr, 93% ee, m.p.:98.6-98.9 ℃; **The ee was determined by HPLC** (Chiralpak AD-H, EtOH/hexane = 20/80, flow rate 1.0 mL/min, λ = 254 nm, major diastereomer: t_{minor} = 20.6 min, t_{major} = 27.6 min); $[\alpha]_D^{20}$ = +23.9 (*c* 1.00, CH_2Cl_2) for major diastereomer;

^{1}H NMR (400 MHz, DMSO-*d*$_6$) δ (major diastereomer) 7.41 (dd, *J* = 6.6, 3.4 Hz, 2H), 7.38 – 7.32 (m, 3H), 7.10 (d, *J* = 7.3 Hz, 2H), 6.78 – 6.71 (m, 1H), 5.93 (d, *J* = 7.8 Hz, 1H), 5.59 (s, 1H), 5.03 (d, *J* = 5.1 Hz, 1H), 4.65 – 4.54 (m, 1H), 4.51 – 4.43 (m, 1H), 3.75 (d, *J* = 5.0 Hz, 1H), 3.05 (ddd, *J* = 12.8, 6.1, 2.1 Hz, 1H), 2.41 – 2.28 (m, 4H).

^{13}C NMR (101 MHz, DMSO-*d*$_6$) δ 175.8, 174.3, 169.9, 168.9, 143.8, 142.4, 136.0, 135.3, 131.4, 130.9, 129.2, 129.0, 128.80, 128.77, 128.52, 128.45, 127.9, 127.7, 127.5, 127.2, 123.8, 122.8, 121.6, 113.8, 102.0, 99.1, 74.4, 73.9, 71.2, 69.5, 68.8, 66.7, 65.9, 64.7, 36.4, 33.8, 24.5, 24.2.

HRMS (ESI-TOF) *m/z*: $[M + H]^+$ calcd for $C_{21}H_{20}N_3O_5$, 394.1397; found, 394.1409.

(1'*R*,3*S*,3a'*R*,8b'*R*)-6'-fluoro-8b'-nitro-1'-phenyl-4'-tosyl-1',3a',4,4',5,8b'-hexahydro-2*H*,2'*H*-spiro[furan-3,3'-pyrrolo[3,4-*b*]indol]-2-one (3d)

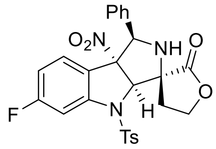

73.0 mg, 70% yield for the sum of the diastereomers, 42% yield for the major isomer, white soild, 60:19:21:0 dr, 92% ee, m.p. 87.6-89.2 ℃; **The ee was determined by HPLC** (Chiralpak AD-H, EtOH/hexane = 20/80, flow rate 1.0 mL/min, λ = 254 nm, major diastereomer: t_{minor} = 10.3 min, t_{major} = 23.9 min); $[\alpha]_D^{20}$ = +45.6 (*c* 1.00, CH_2Cl_2) for major diastereomer;

^{1}H NMR (400 MHz, DMSO-*d*$_6$) δ (major diastereomer) 7.55 (d, *J* = 8.2 Hz, 2H), 7.43 (dd, *J* = 9.6, 2.5 Hz, 1H), 7.37 (d, *J* = 7.3 Hz, 1H), 7.31 (dd, *J* = 8.0, 3.8 Hz, 4H), 7.07 (d, *J* = 7.5 Hz, 2H), 6.75 (m, *J* = 8.8, 2.4 Hz, 1H), 5.76 (dd, *J* = 8.7, 5.5 Hz, 1H), 5.53 (s, 1H), 5.30 (d, *J* = 6.0 Hz, 1H), 4.69 (d, *J* = 6.0 Hz, 1H), 4.58 (td, *J* = 8.5, 3.8 Hz, 1H), 4.45 (m, *J* = 8.2 Hz, 1H), 3.01 (dt, *J* = 13.6, 8.3 Hz, 1H), 2.38 – 2.31 (m, 1H), 2.30 (s, 3H).

^{13}C NMR (101 MHz, DMSO-*d*$_6$) δ (major diastereomer) 176.9, 165.4, 163.0, 145.8, 145.2 (d, *J* = 12.3 Hz), 134.4, 131.5, 129.5 (d, *J* = 10.7 Hz), 129.2, 128.9 (d, *J* = 262.8 Hz), 128.5, 128.0, 120.4 (d, *J* = 2.4 Hz), 111.9 (d, *J* = 23.4 Hz), 103.9 (d, *J* = 28.0 Hz), 100.4, 74.7, 66.9, 66.6, 66.5, 31.6, 21.0.

HRMS (ESI-TOF) *m/z*: $[M + H]^+$ calcd for $C_{26}H_{23}FN_3O_6S$, 524.1286; found, 524.1288.

(1'*R*,3*S*,3a'*R*,8b'*R*)-5'-fluoro-8b'-nitro-1'-phenyl-4'-tosyl-1',3a',4,4',5,8b'-hexahydro-2*H*,2'*H*-spiro[furan-3,3'-pyrrolo[3,4-*b*]indol]-2-one (3e)

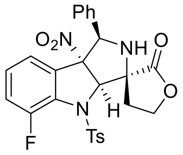

94.2 mg, 90% yield for the sum of the diastereomers, 65% yield for the major isomer, white soild, 72:28:0:0 dr, 93% ee, m.p. 88.4-89.3 ℃; **The ee was determined by HPLC** (Chiralpak AD-H, EtOH/hexane = 20/80, flow rate 1.0 mL/min, λ = 220 nm, major diastereomer: t_{minor} = 14.6 min, t_{major} = 31.5 min); $[\alpha]_D^{20}$ = +22.9 (*c* 1.00, CH_2Cl_2) for major diastereomer;

^{1}H NMR (400 MHz, DMSO-d_6) δ (major diastereomer) 7.41 (dd, *J* = 8.5, 2.1 Hz, 3H), 7.38 – 7.35 (m, 2H), 7.31 – 7.27 (m, 4H), 6.99 – 6.89 (m, 3H), 5.60 (d, *J* = 7.8 Hz, 1H), 5.58 (s, 1H), 5.32 (d, *J* = 6.5 Hz, 1H), 4.62 (d, *J* = 6.5 Hz, 1H), 4.53 (dd, *J* = 8.4, 4.5 Hz, 1H), 4.46 – 4.40 (m, 1H), 2.85 (dt, *J* = 13.6, 7.9 Hz, 1H), 2.39 – 2.32 (m, 1H), 2.31 (s, 3H).

^{13}C NMR (101 MHz, DMSO-d_6) δ (major diastereomer) 176.9, 152.7, 150.2, 145.7, 132.7 (d, *J* = 264.1 Hz), 130.2, 129.2, 128.4 (d, *J* = 2.0 Hz), 128.3, 128.1, 127.8 (d, *J* = 44.9 Hz), 127.5 (d, *J* = 52.4 Hz), 126.7 (d, *J* = 6.9 Hz), 124.2, 119.8 (d, *J* = 19.7 Hz), 100.7, 75.3, 67.9, 66.9, 66.4, 31.8, 21.1.

HRMS (ESI-TOF) *m/z*: $[M + H]^+$ calcd for $C_{26}H_{23}FN_3O_6S$, 524.1286; found, 524.1291.

(1'*R*,3*S*,3a'*R*,8b'*R*)-7'-chloro-8b'-nitro-1'-phenyl-4'-tosyl-1',3a',4,4',5,8b'-hexahydro-2*H*,2'*H*-spiro[furan-3,3'-pyrrolo[3,4-*b*]indol]-2-one (3f)

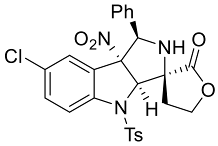

97.2 mg, 90% yield for the sum of the diastereomers, 55% yield for the major isomer, white soild, 61:15:24:0 dr, 95% ee, m.p.: 93.8-94.2 ℃; **The ee was determined by HPLC** (Chiralpak AD-H, EtOH/hexane = 20/80, flow rate 1.0 mL/min, λ = 254 nm, major diastereomer: t_{minor} = 11.0 min, t_{major} = 19.0 min); $[\alpha]_D^{20}$ = +83.4 (*c* 1.00, CH_2Cl_2) for major diastereomer;

^{1}H NMR (400 MHz, DMSO-d_6) δ (major diastereomer) 7.70 (d, *J* = 8.8 Hz, 1H), 7.55 (dd, *J* = 8.8, 2.3 Hz, 1H), 7.49 (d, *J* = 8.2 Hz, 2H), 7.41 (d, *J* = 7.3 Hz, 1H), 7.34 (d, *J* = 7.6 Hz, 2H), 7.29 (d, *J* = 8.2 Hz, 2H), 7.06 (d, *J* = 7.6 Hz, 2H), 5.57 (d, *J* = 2.2 Hz, 1H), 5.49 (s, 1H), 5.33 (d, *J* = 5.9 Hz, 1H), 4.71 (d, *J* = 5.9 Hz, 1H), 4.59 (td, *J* = 8.5, 4.0 Hz, 1H), 4.47 – 4.43 (m, 1H), 2.99 (dt, *J* = 13.6, 8.2 Hz, 1H), 2.35 – 2.30 (m, 1H), 2.29 (s, 3H).

^{13}C NMR (101 MHz, DMSO-d_6) δ (major diastereomer) 176.9, 145.7, 142.5, 134.3, 132.2, 131.5, 130.2, 129.3, 128.38, 128.36, 128.0, 127.6, 127.5, 125.9, 117.9, 100.6, 74.3, 67.1, 66.8, 66.6, 31.6, 21.0.

HRMS (ESI-TOF) *m/z*: $[M + H]^+$ calcd for $C_{26}H_{23}{}^{35}ClN_3O_6S$, 540.0991; found, 540.0991. $[M + H]^+$ calcd for $C_{26}H_{23}{}^{37}ClN_3O_6S$, 542.0972; found, 542.0972.

(1'*R*,3*S*,3a'*R*,8b'*R*)-6'-chloro-8b'-nitro-1'-phenyl-4'-tosyl-1',3a',4,4',5,8b'-hexahydro-2*H*,2'*H*-spiro[furan-3,3'-pyrrolo[3,4-*b*]indol]-2-one (3g)

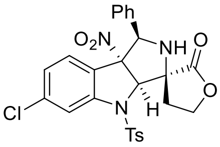

85.4 mg, 79% yield for the sum of the diastereomers, 54% yield for the major isomer, white soild, 68:11:21:0 dr, 92% ee, m.p. 83.6-84.5 ℃; **The ee was determined by HPLC** (Chiralpak AD-H, EtOH/hexane = 20/80, flow rate 1.0 mL/min, λ = 254 nm, major diastereomer: t_{minor} = 9.2 min, t_{major} = 24.5 min); $[\alpha]_D^{20}$ = +96.7 (*c* 1.00, CH_2Cl_2) for major diastereomer;

^{1}H NMR (400 MHz, DMSO-*d*$_6$) δ (major diastereomer) 7.66 (d, *J* = 2.0 Hz, 1H), 7.52 (d, *J* = 8.3 Hz, 2H), 7.38 (d, *J* = 7.4 Hz, 1H), 7.31 (m, *J* = 7.2, 3.3 Hz, 4H), 7.07 (d, *J* = 7.5 Hz, 2H), 6.97 (dd, *J* = 8.4, 2.0 Hz, 1H), 5.74 (d, *J* = 8.5 Hz, 1H), 5.51 (s, 1H), 5.32 (d, *J* = 6.2 Hz, 1H), 4.69 (d, *J* = 6.2 Hz, 1H), 4.58 (td, *J* = 8.5, 3.9 Hz, 1H), 4.45 (td, *J* = 8.5, 6.8 Hz, 1H), 3.00 (dt, *J* = 13.6, 8.3 Hz, 1H), 2.36 – 2.31 (m, 1H), 2.30 (s, 3H).

^{13}C NMR (101 MHz, DMSO-*d*$_6$) δ (major diastereomer) 176.8, 145.8, 144.7, 136.8, 134.2, 131.5, 130.2, 129.24, 129.17, 128.4, 128.0, 127.5, 124.7, 123.2, 116.1, 100.5, 74.4, 67.0, 66.7, 66.6, 31.6, 21.0.

HRMS (ESI-TOF) *m/z*: $[M + H]^+$ calcd for $C_{26}H_{23}{}^{35}ClN_3O_6S$, 540.0991; found, 540.0993. $[M + H]^+$ calcd for $C_{26}H_{23}{}^{37}ClN_3O_6S$, 542.0972; found, 542.0973.

(1'*R*,3*S*,3a'*R*,8b'*R*)-5'-chloro-8b'-nitro-1'-phenyl-4'-tosyl-1',3a',4,4',5,8b'-hexahydro-2*H*,2'*H*-spiro[furan-3,3'-pyrrolo[3,4-*b*]indol]-2-one (3h)

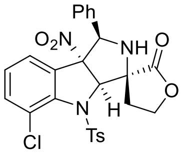

68.1 mg, 63% yield for the sum of the diastereomers, 49% yield for the major isomer, white soild, 77:23:0:0 dr, 96% ee, m.p.:90.5-91.5 ℃; **The ee was determined by HPLC** (Chiralpak AD-H, EtOH/hexane = 20/80, flow rate 1.0 mL/min, λ = 254 nm, major diastereomer: t_{minor} = 11.9 min, t_{major} = 30.3 min); $[\alpha]_D^{20}$ = +1.6 (*c* 0.10, CH_2Cl_2) for major diastereomer;

^{1}H NMR (400 MHz, DMSO-*d*$_6$) δ (major diastereomer) 7.65 (dd, *J* = 8.0, 1.2 Hz, 1H), 7.43 – 7.40 (m, 1H), 7.35 – 7.28 (m, 7H), 7.10 – 7.01 (m, 1H), 6.86 (d, *J* = 7.2 Hz, 2H), 5.73 (d, *J* = 6.7 Hz, 1H), 5.43 (s, 1H), 5.30 (d, *J* = 6.6 Hz, 1H), 4.52 (d, *J* = 6.6 Hz, 1H), 4.38 – 4.32 (m, 2H), 2.60 (dt, *J* = 14.1, 7.3 Hz, 1H), 2.35 (s, 3H), 2.22 (dt, *J* = 13.0, 5.9 Hz, 1H).

^{13}C NMR (101 MHz, DMSO-*d*$_6$) δ (major diastereomer) 177.0, 145.9, 140.4, 133.6, 133.5, 131.4, 130.3, 130.2, 129.4, 128.7, 128.2, 127.7, 127.6, 127.4, 125.1, 100.5, 74.5, 69.0, 66.5, 66.0, 31.9, 21.1.

HRMS (ESI-TOF) *m/z*: $[M + H]^+$ calcd for $C_{26}H_{23}{}^{35}ClN_3O_6S$, 540.0991; found, 540.0994. $[M + H]^+$ calcd for $C_{26}H_{23}{}^{37}ClN_3O_6S$, 542.0972; found, 540.0975.

(1'*R*,3*S*,3a'*R*,8b'*R*)-8b'-nitro-1'-(o-tolyl)-4'-tosyl-1',3a',4,4',5,8b'-hexahydro-2*H*,2'*H*-spiro[furan-3,3'-pyrrolo[3,4-*b*]indol]-2-one (3i)

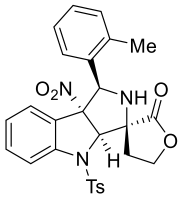

95.5 mg, 92% yield for the sum of the diastereomers, 56% yield for the major isomer, white sild, 61:18:21:0 dr, 78% ee, m.p. :116.8-117.4 ℃; **The ee was determined by HPLC** (Chiralpak AD,

EtOH/hexane = 20/80, flow rate 1.0 mL/min, λ = 254 nm, major diastereomer: t_{minor} = 9.0 min, t_{major} = 21.1 min); $[\alpha]_D^{20}$ = +29.1 (*c* 1.00, CH_2Cl_2) for major diastereomer;

^{1}H NMR (400 MHz, DMSO-*d*$_6$) δ (major diastereomer) 7.71 (d, *J* = 8.2 Hz, 1H), 7.55 – 7.50 (m, 1H), 7.42 (d, *J* = 8.3 Hz, 2H), 7.24 (d, *J* = 8.8 Hz, 4H), 7.02 – 6.92 (m, 2H), 6.72 (d, *J* = 8.5 Hz, 1H), 6.04 (s, 1H), 5.62 (d, *J* = 5.6 Hz, 1H), 5.55 (s, 1H), 4.58 (td, *J* = 8.4, 4.2 Hz, 1H), 4.53 (d, *J* = 5.6 Hz, 1H), 4.45 – 4.40 (m, 1H), 2.98 (dt, *J* = 13.5, 8.1 Hz, 1H), 2.31 – 2.28 (m, 1H), 2.26 (s, 3H), 2.12 (s, 3H).

^{13}C NMR (101 MHz, DMSO-*d*$_6$) δ (major diastereomer) 181.2, 145.4, 143.5, 137.0, 132.3, 132.2, 131.6, 130.5, 130.0, 129.4, 129.3, 128.6, 127.3, 124.8, 124.3, 123.7, 116.3, 100.5, 73.7, 66.6, 66.4, 62.9, 31.8, 21.0, 18.6.

HRMS (ESI-TOF) *m/z*: $[M + H]^+$ calcd for $C_{27}H_{26}N_3O_6S$, 520.1537; found, 520.1541.

(1'*R*,3*S*,3a'*R*,8b'*R*)-8b'-nitro-1'-(m-tolyl)-4'-tosyl-1',3a',4,4',5,8b'-hexahydro-2*H*,2'*H*-spiro[furan-3,3'-pyrrolo[3,4-*b*]indol]-2-one (3j)

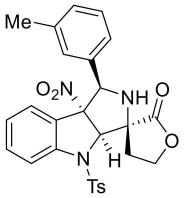

101.8 mg, 98% yield for the sum of the diastereomers, 61% yield for the major isomer, white soild, 62:18:20:0 dr, 90% ee, m.p.:190.2-190.8 ℃; **The ee was determined by HPLC** (Chiralpak AD, EtOH/hexane = 20/80, flow rate 1.0 mL/min, λ = 254 nm, major diastereomer: t_{minor} = 12.1 min, t_{major} = 30.9 min); $[\alpha]_D^{20}$ = +30.7 (*c* 1.00, CH_2Cl_2)

^{1}H NMR (400 MHz, DMSO-*d*$_6$) δ (major diastereomer) 7.69 (d, *J* = 8.2 Hz, 1H), 7.48 (m, *J* = 8.4 Hz, 3H), 7.26 (d, *J* = 8.2 Hz, 2H), 7.18 (d, *J* = 4.6 Hz, 2H), 6.89 – 6.83 (m, 2H), 6.82 – 6.78 (m, 1H), 5.80 (d, *J* = 6.6 Hz, 1H), 5.48 (s, 1H), 5.28 (d, *J* = 5.6 Hz, 1H), 4.58 (td, *J* = 8.3, 3.9 Hz, 2H), 4.48 – 4.41 (m, 1H), 3.01 (dt, *J* = 13.6, 8.2 Hz, 1H), 2.34 – 2.29 (m, 1H), 2.28 (s, 3H), 2.19 (s, 3H).

^{13}C NMR (101 MHz, DMSO-*d*$_6$) δ (major diastereomer) 177.1, 145.4, 143.5, 136.9, 134.5, 132.1, 131.8, 130.0, 129.6, 129.1, 127.9, 127.7, 127.5, 125.8, 124.3, 124.2, 116.3, 101.1, 73.9, 67.0, 66.7, 66.6, 31.6, 21.0, 20.9.

HRMS (ESI-TOF) *m/z*: $[M + H]^+$ calcd for $C_{27}H_{26}N_3O_6S$, 520.1537; found, 520.1538.

(1'*R*,3*S*,3a'*R*,8b'*R*)-1'-(4-isopropylphenyl)-8b'-nitro-4'-tosyl-1',3a',4,4',5,8b'-hexahydro-2*H*,2'*H*-spiro[furan-3,3'-pyrrolo[3,4-*b*]indol]-2-one (3k)

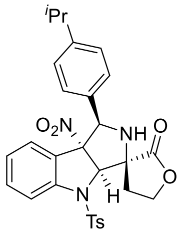

108.4 mg, 99% yield for the sum of the diastereomers, 55% yield for the major isomer, white soild, 56:18:16:0 dr, 90% ee, m.p.:109.8-110.3 ℃; **The ee was determined by HPLC** (Chiralpak IC,

EtOH/hexane = 10/90, flow rate 1.0 mL/min, λ = 254 nm, major diastereomer: t_{minor} = 12.4 min, t_{major} = 14.0 min); $[\alpha]_D^{20}$ = +18.4 (*c* 1.00, CH_2Cl_2) for major diastereomer;

^{1}H NMR (400 MHz, DMSO-*d*$_6$) δ (major diastereomer) 7.68 (d, *J* = 8.2 Hz, 1H), 7.51 – 7.44 (m, 3H), 7.26 (d, *J* = 8.3 Hz, 2H), 7.15 (d, *J* = 7.9 Hz, 2H), 6.96 (d, *J* = 8.0 Hz, 2H), 6.86 – 6.78 (m, 1H), 5.80 (d, *J* = 7.9 Hz, 1H), 5.50 (s, 1H), 5.28 (d, *J* = 5.6 Hz, 1H), 4.61 – 4.56 (m, 2H), 4.47 – 4.40 (m, 1H), 3.03 (dt, *J* = 13.6, 8.2 Hz, 1H), 2.92 – 2.84 (m, 1H), 2.33 – 2.29 (m, 1H), 2.27 (s, 3H), 1.18 (dd, *J* = 6.9, 3.1 Hz, 6H).

^{13}C NMR (101 MHz, DMSO-*d*$_6$) δ (major diastereomer) 177.1, 149.4, 145.3, 143.5, 132.10, 132.05, 131.8, 129.9, 128.6, 127.7, 127.5, 125.6, 124.32, 124.27, 116.1, 101.1, 73.8, 66.9, 66.6, 66.4, 33.1, 31.6, 23.9, 23.8, 21.0.

HRMS (ESI-TOF) *m/z*: $[M + H]^+$ calcd for $C_{29}H_{30}N_3O_6S$, 548.1850; found, 548.1852.

(1'*R*,3*S*,3a'*R*,8b'*R*)-1'-(4-chlorophenyl)-8b'-nitro-4'-tosyl-1',3a',4,4',5,8b'-hexahydro-2*H*,2'*H*-spiro[furan-3,3'-pyrrolo[3,4-*b*]indol]-2-one (3l)

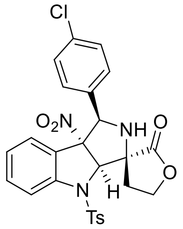

88.6 mg, 82% yield for the sum of the diastereomers, 38% yield for the major isomer, white soild, 47:23:30:0 dr, 88% ee m.p.:109.6-110.2 ℃; **The ee was determined by HPLC** (Chiralpak AD, EtOH/hexane = 20/80, flow rate 1.0 mL/min, λ = 254 nm, major diastereomer: t_{minor} = 14.6 min, t_{major} = 47.9 min); $[\alpha]_D^{20}$ = +20.9 (*c* 1.00, CH_2Cl_2) for major diastereomer.

^{1}H NMR (400 MHz, DMSO-*d*$_6$) δ (major diastereomer) 7.70 (d, *J* = 8.2 Hz, 1H), 7.52 (d, *J* = 8.4 Hz, 2H), 7.50 – 7.46 (m, 1H), 7.36 (d, *J* = 8.7 Hz, 2H), 7.27 (d, *J* = 8.1 Hz, 2H), 7.09 (d, *J* = 8.1 Hz, 2H), 6.92 – 6.86 (m, 1H), 5.88 (d, *J* = 9.1 Hz, 1H), 5.50 (s, 1H), 5.32 (d, *J* = 5.5 Hz, 1H), 4.70 (d, *J* = 4.3 Hz, 1H), 4.59 (td, *J* = 8.5, 3.9 Hz, 1H), 4.49 – 4.42 (m, 1H), 3.07 (dt, *J* = 13.6, 8.3 Hz, 1H), 2.30 (dd, *J* = 6.8, 3.9 Hz, 1H), 2.28 (s, 3H).

^{13}C NMR (101 MHz, DMSO-*d*$_6$) δ (major diastereomer) 177.0, 145.4, 143.5, 134.0, 133.5, 132.3, 131.7, 130.7, 130.0, 127.8, 127.6, 127.2, 124.5, 124.2, 116.3, 100.8, 73.6, 66.8, 66.7, 65.5, 31.6, 21.0.

HRMS (ESI-TOF) *m/z*: $[M + H]^+$ calcd for $C_{26}H_{23}{}^{35}ClN_3O_6S$, 540.0991; found, 540.0993. $[M + H]^+$ calcd for $C_{26}H_{23}{}^{37}ClN_3O_6S$, 542.0972; found, 542.0975.

(1'*R*,3*S*,3a'*R*,8b'*R*)-1'-(2,4-dimethylphenyl)-8b'-nitro-4'-tosyl-1',3a',4,4',5,8b'-hexahydro-2*H*, 2'*H*-spiro[furan-3,3'-pyrrolo[3,4-*b*]indol]-2-one (3m)

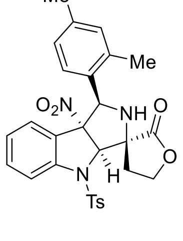

87.5 mg, 82% yield for the sum of the diastereomers, 48% yield for the major isomer, white soild, 59:15:26:0 dr, 80% ee, m.p.:84.6-85.1 ℃; **The ee was determined by HPLC** (Chiralpak IC, EtOH/hexane = 15/85, flow rate 1.0 mL/min, λ = 254 nm, major diastereomer: t_{minor} = 9.9 min, t_{major} = 12.7 min); $[\alpha]_D^{20}$ = +28.3 (*c* 1.00, CH_2Cl_2) for major diastereomer;
^{1}H NMR (400 MHz, DMSO-*d*$_6$) δ (major diastereomer) 7.69 (d, *J* = 8.2 Hz, 1H), 7.48 (dd, *J* = 7.9, 3.1 Hz, 3H), 7.26 (d, *J* = 8.1 Hz, 2H), 7.05 (d, *J* = 7.8 Hz, 1H), 6.90 – 6.84 (m, 1H), 6.79 – 6.72 (m, 2H), 5.86 (d, *J* = 7.1 Hz, 1H), 5.48 (s, 1H), 5.24 (d, *J* = 6.2 Hz, 1H), 4.58 (dd, *J* = 8.5, 4.0 Hz, 1H), 4.53 (d, *J* = 6.2 Hz, 1H), 4.43 (m, *J* = 8.0 Hz, 1H), 3.00 (dt, *J* = 13.6, 8.2 Hz, 1H), 2.34 – 2.29 (m, 1H), 2.27 (s, 3H), 2.21 (s, 3H), 2.11 (s, 3H).
^{13}C NMR (101 MHz, DMSO-*d*$_6$) δ (major diastereomer) 177.1, 145.4, 143.5, 136.9, 135.6, 132.1, 131.84, 131.76, 130.2, 130.0, 129.6, 128.9, 128.1, 127.4, 126.0, 124.3, 116.2, 101.1, 73.9, 66.9, 66.7, 66.6, 31.6, 21.0, 19.3, 19.2.
HRMS (ESI-TOF) *m/z*: $[M + H]^+$ calcd for $C_{28}H_{28}N_3O_6S$, 534.1693; found, 534.1696.

(1'*R*,3*S*,3a'*R*,8b'*R*)-1'-(naphthalen-2-yl)-8b'-nitro-4'-tosyl-1',3a',4,4',5,8b'-hexahydro-2*H*,2'*H* -spiro[furan-3,3'-pyrrolo[3,4-*b*]indol]-2-one (3n)

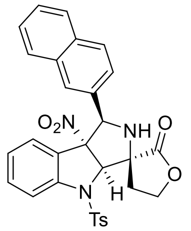

106.6 mg, 96% yield for the sum of the diastereomers, 67% yield for the major isomer, white soild, 70:15:15:0 dr, 69% ee, m.p.:117.4-117.9 ℃; **The ee was determined by HPLC** (Chiralpak AS, EtOH/hexane = 20/80, flow rate 1.0 mL/min, λ = 254 nm, major diastereomer: t_{minor} = 16.9 min, t_{major} = 23.2 min); $[\alpha]_D^{20}$ = +23.5 (*c* 1.00, CH_2Cl_2) for major diastereomer;
^{1}H NMR (400 MHz, DMSO-*d*$_6$) δ (major diastereomer) 7.91 (d, *J* = 7.7 Hz, 1H), 7.81 (d, *J* = 8.3 Hz, 2H), 7.71 (d, *J* = 8.3 Hz, 1H), 7.64 (d, *J* = 4.4 Hz, 1H), 7.51 (d, *J* = 7.6 Hz, 4H), 7.44 (d, *J* = 8.2 Hz, 1H), 7.27 (d, *J* = 8.2 Hz, 2H), 7.13 (d, *J* = 8.5 Hz, 1H), 6.74 – 6.67 (m, 1H), 5.73 (d, *J* = 8.1 Hz, 1H), 5.54 (s, 1H), 5.51 (d, *J* = 5.5 Hz, 1H), 4.77 (d, *J* = 5.7 Hz, 1H), 4.64 – 4.58 (m, 1H), 4.48 (q, *J* = 7.6 Hz, 1H), 3.08 (dt, *J* = 12.6, 8.0 Hz, 1H), 2.38 – 2.34 (m, 1H), 2.27 (s, 3H).
^{13}C NMR (101 MHz, DMSO-*d*$_6$) δ (major diastereomer) 177.2, 145.4, 143.6, 133.1, 132.3, 132.24, 132.19, 131.8, 130.0, 128.1, 127.60, 127.56, 127.2, 127.1, 126.8, 126.6, 126.4, 126.2, 124.38, 124.35, 116.3, 101.0, 73.9, 67.0, 66.6, 31.7, 26.4, 21.0.
HRMS (ESI-TOF) *m/z*: $[M + H]^+$ calcd for $C_{30}H_{26}N_3O_6S$, 556.1537; found, 556.1540.

(1'*R*,3*S*,3a'*R*,8b'*R*)-1'-(furan-3-yl)-8b'-nitro-4'-tosyl-1',3a',4,4',5,8b'-hexahydro-2*H*,2'*H*-spiro [furan-3,3'-pyrrolo[3,4-*b*]indol]-2-one (3o)

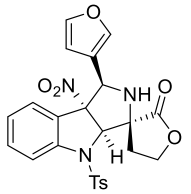

74.3 mg, 75% yield for the sum of the diastereomers, 40% yield for the major isomer, white soild 54:12:34:0 dr, 91% ee, m.p.:121.2-121.6 ℃; **The ee was determined by HPLC** (Chiralpak AD, EtOH/hexane = 20/80, flow rate 1.0 mL/min, λ = 220 nm, major diastereomer: t_{minor} = 16.1 min, t_{major} = 34.7 min); $[\alpha]_D^{20}$ = +12.5 (*c* 1.00, CH_2Cl_2) for major diastereomer.

^{1}H NMR (400 MHz, DMSO-*d*$_6$) δ (major diasteremre) 7.70 (d, *J* = 8.1 Hz, 1H), 7.64 – 7.63 (m, 1H), 7.55 – 7.49 (m, 4H), 7.30 – 7.26 (m, 2H), 7.03 – 6.97 (m, 1H), 6.42 (dd, *J* = 7.8, 1.3 Hz, 1H), 6.22 (d, *J* = 1.2 Hz, 1H), 5.50 (s, 1H), 5.22 (d, *J* = 7.9 Hz, 1H), 4.56 (dd, *J* = 8.5, 3.4 Hz, 1H), 4.44 (dd, *J* = 15.0, 7.2 Hz, 2H), 3.01 (dt, *J* = 13.6, 8.5 Hz, 1H), 2.33 – 2.30 (m, 1H), 2.29 (s, 3H).

^{13}C NMR (101 MHz, DMSO-*d*$_6$) δ 176.8, 175.5, 145.5, 145.4, 143.9, 143.6, 143.2, 142.2, 141.9, 141.5, 132.3, 132.2, 131.9, 131.8, 130.2, 129.9, 128.1, 127.6, 127.3, 126.7, 126.14, 126.11, 124.6, 124.4, 119.8, 119.5, 116.3, 116.1, 110.5, 109.2, 102.6, 101.0, 74.7, 74.1, 68.2, 67.4, 66.8, 66.4, 65.4, 60.1, 31.5, 31.2, 28.1, 21.0.

HRMS (ESI-TOF) *m/z*: $[M + H]^+$ calcd for $C_{24}H_{22}N_3O_7S$, 496.1173; found, 496.1174.

2. Optimization of conditions for asymmetric synthesis of 5

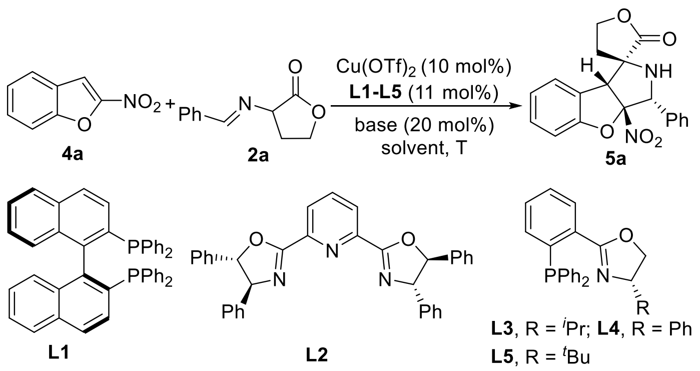

entry	L1-L5	base	solvent	T (°C)	T (h)	yield (%) [a]	dr [b]	ee (%) [c]
1	**L1**	Cs_2CO_3	THF	25	36	95	50:50:nd	24/70
2	**L2**	Cs_2CO_3	THF	25	23	95	80:20:nd	2/31
3	**L3**	Cs_2CO_3	THF	25	23	73	39:61:nd	9/22
4	**L4**	Cs_2CO_3	THF	25	36	86	27:73:nd	3/86
5	**L5**	Cs_2CO_3	THF	25	36	98	10:90:nd	7/76
6	**L4**	Cs_2CO_3	1,4-dioxane	25	60	94	55:45:nd	0/79
7	**L4**	Cs_2CO_3	DCM	25	60	94	40:60:nd	4/81
8	**L4**	Cs_2CO_3	MTBE	25	60	54	28:72:nd	3/69
9	**L4**	Cs_2CO_3	DCE	25	60	99	17:83:nd	11/83
10	**L4**	Cs_2CO_3	MeCN	25	60	95	48:52:nd	3/69
11	**L4**	Cs_2CO_3	toluene	25	60	50	12:88:nd	25/78
12	**L4**	Cs_2CO_3	EA	25	36	57	35:65:nd	0/76
13	**L4**	Cs_2CO_3	MeOH	25	36	74	24:76:nd	3/85
14	**L4**	Cs_2CO_3	PhF	25	36	51	7:93:nd	31/84
15	**L4**	Cs_2CO_3	PhCl	25	36	82	14:86:nd	28/85
16	**L4**	Cs_2CO_3	2-Me-THF	25	36	98	41:59:nd	5/77
17	**L4**	K_2CO_3	PhCl	25	59	67	20:80:nd	0/84
18	**L4**	DBU	PhCl	25	59	75	57:43:nd	6/66
19	**L4**	DIPEA	PhCl	25	82	17	27:73:nd	16/52

20	**L4**	Et_3N	PhCl	25	82	13	22:78:nd	20/40
21	**L4**	DABCO	PhCl	25	82	22	26:74:nd	7/55
22	**L4**	TMG	PhCl	25	82	61	94:6:nd	3/46
23	**L4**	Cs_2CO_3	PhCl	50	72	70	17:83:nd	20/80
24	**L4**	Cs_2CO_3	PhCl	30	72	68	18:82:nd	12,/84
25	**L4**	Cs_2CO_3	PhCl	0	72	53	11:89:nd	11/89
26	**L4**	Cs_2CO_3	PhCl	-30	72	41	12:88:nd	12/88
**27 [d]**	**L4**	**Cs_2CO_3**	**PhCl**	**30**	**45**	**81**	**24:76**:nd	**11/96**

[a] Isolated yield of the mixture of diastereomers. [b] Determined by ^{1}H NMR. [c] Determined by chiral HPLC analysis after purification of the diastereomers. [d] 10 mol% Cs_2CO_3 was added.

3. Characterization data for compounds 5

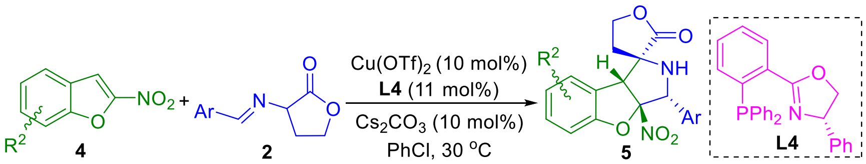

(1*R*,3*R*,3a*R*,8b*R*)-3a-nitro-3-phenyl-2,3,3a,4',5',8b-hexahydro-2'*H*-spiro[benzofuro[2,3-*c*]pyrrole-1,3'-furan]-2'-one(5a)

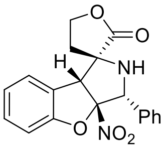

57.1 mg, 81% yield for the sum of the diastereomers, 61% yield for the major isomer, white soild, 74:26 dr, 96% ee, m.p.:160.9-161.4 ℃; **The ee was determined by HPLC** (Chiralpak IB, *i*-PrOH/hexane = 20/80, flow rate 1.0 mL/min, λ = 220 nm, major diastereomer: t_{minor} = 12.7 min, t_{major} = 9.9 min); $[\alpha]_D^{20}$ = -91.2 (*c* 1.00, CH_2Cl_2) for major diastereomer;

^{1}H NMR (400 MHz, DMSO-*d*$_6$) δ (major diastereomer) 7.43 – 7.38 (m, 3H), 7.34 (dd, *J* = 7.2, 2.9 Hz, 2H), 7.29 – 7.24 (m, 1H), 7.07 (d, *J* = 7.2 Hz, 1H), 7.02 (dd, *J* = 12.7, 7.6 Hz, 2H), 5.23 (d, *J* = 6.6 Hz, 1H), 4.85 (s, 1H), 4.67 – 4.57 (m, 2H), 3.83 (d, *J* = 7.0 Hz, 1H), 2.95 (dd, *J* = 12.3, 4.9 Hz, 1H), 2.45 (dd, *J* = 11.7, 8.5 Hz, 1H).

^{13}C NMR (101 MHz, DMSO-*d*$_6$) δ (major diastereomer) 173.6, 157.9, 133.9, 129.7, 128.8, 128.3, 128.0, 125.5, 123.8, 122.8, 122.7, 109.6, 68.4, 68.1, 64.6, 60.5, 36.2.

HRMS (ESI-TOF) *m/z*: $[M + H]^+$ calcd for $C_{19}H_{17}N_2O_5$, 353.1132; found, 353.1136.

(1*R*,3*R*,3a*S*,8b*R*)-7-fluoro-3a-nitro-3-phenyl-2,3,3a,4',5',8b-hexahydro-2'*H*-spiro[benzofuro[2,3-*c*]pyrrole-1,3'-furan]-2'-one(5b)

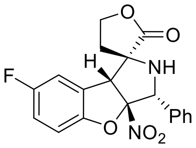

60.7 mg, 82% yield for the major isomer, white soild, >99:1 dr, 95% ee, m.p.: 70.2-70.8 ℃; **The ee was determined by HPLC** (Chiralpak AD, *i*-PrOH/hexane = 20/80, flow rate 1.0 mL/min, λ = 254 nm, major diastereomer: t_{minor} = 10.2 min, t_{major} = 11.8 min); $[\alpha]_D^{20}$ = -96.2 (*c* 1.00, CH_2Cl_2) for major diastereomer;

^{1}H NMR (400 MHz, CDCl$_3$) δ (major diastereomer) 7.44 – 7.38 (m, 3H), 7.29 – 7.26 (m, 2H), 7.02 – 6.95 (m, 1H), 6.93 (dd, *J* = 8.8, 4.1 Hz, 1H), 6.78 – 6.73 (m, 1H), 5.15 (d, *J* = 13.0 Hz, 1H), 4.75 – 4.68 (m, 1H), 4.48 (td, *J* = 10.8, 5.2 Hz, 1H), 4.41 (s, 1H), 3.29 (d, *J* = 13.4 Hz, 1H), 3.05 (dd, *J* = 12.7, 5.3 Hz, 1H), 2.64 (td, *J* = 12.1, 8.6 Hz, 1H).

^{13}C NMR (101 MHz, DMSO-*d*$_6$) δ (major diastereomer) 173.6, 157.8 (d, *J* = 237.6 Hz), 154.1, 133.8, 128.8, 128.2 (d, *J* = 24.3 Hz), 125.7 (d, *J* = 9.5 Hz), 123.2, 116.2 (d, *J* = 24.5 Hz), 112.7 (d, *J* = 25.9 Hz), 110.5 (d, *J* = 8.8 Hz), 68.4, 68.1, 64.7, 60.51, 60.49, 36.1.

HRMS (ESI-TOF) *m/z*: [M + H]$^+$ calcd for $C_{19}H_{16}FN_2O_5$, 371.1038; found, 371.0147.

(1*R*,3*R*,3a*S*,8b*R*)-7-chloro-3a-nitro-3-phenyl-2,3,3a,4',5',8b-hexahydro-2'*H*-spiro[benzofuro[2,3-*c*]pyrrole-1,3'-furan]-2'-one(5c)

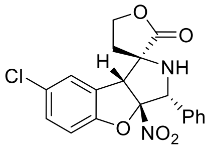

66.5 mg, 86% yield for the major isomer, white soild, >99:1 dr, 94% ee, m.p.: 90.6-90.9 ℃; **The ee was determined by HPLC** (Chiralpak IB, EtOH/hexane = 5/95, flow rate 1.0 mL/min, λ = 220 nm, major diastereomer: t_{minor} = 22.9 min, t_{major} = 19.6 min); $[\alpha]_D^{20}$ = -83.7 (*c* 1.00, CH_2Cl_2) for major diastereomer;

^{1}H NMR (400 MHz, CDCl$_3$) δ (major diastereomer) 7.43 – 7.37 (m, 3H), 7.37 – 7.30 (m, 3H), 7.12 (s, 1H), 7.06 (d, *J* = 8.6 Hz, 1H), 5.20 (d, *J* = 6.4 Hz, 1H), 4.84 (s, 1H), 4.63 (dd, *J* = 10.7, 5.3 Hz, 1H), 4.61 – 4.53 (m, 1H), 3.93 (d, *J* = 6.4 Hz, 1H), 2.94 (dd, *J* = 12.6, 5.1 Hz, 1H), 2.49 – 2.43 (m, 1H).

^{13}C NMR (101 MHz, DMSO-*d*$_6$) δ (major diastereomer) 173.6, 156.8, 133.8, 129.6, 128.9, 128.3, 128.1, 126.4, 126.3, 125.5, 122.9, 111.1, 68.4, 68.2, 64.8, 60.2, 36.1.

HRMS (ESI-TOF) *m/z*: [M + H]$^+$ calcd for $C_{19}H_{16}{}^{35}ClN_2O_5$, 387.0742; found, 387.0754 . [M + H]$^+$ calcd for $C_{19}H_{16}{}^{37}ClN_2O_5$, 389.0721; found, 387.0731 .

(1*R*,3*R*,3a*S*,8b*R*)-7-bromo-3a-nitro-3-phenyl-2,3,3a,4',5',8b-hexahydro-2'*H*-spiro[benzofuro[2,3-*c*]pyrrole-1,3'-furan]-2'-one(5d)

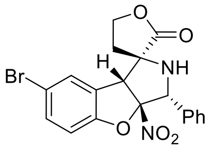

69.0 mg. 80% yield for the major isomer, white soild, >99:1 dr, 98% ee, m.p.: 68.4-68.8 ℃; **The ee was determined by HPLC** (Chiralpak IA, *i*-PrOH/hexane = 10/90, flow rate 1.0 mL/min, λ = 254 nm, major diastereomer: t_{minor} = 18.4 min, t_{major} = 24.8 min),ee 98%; $[\alpha]_D^{20}$ = -84.6 (*c* 1.00, CH_2Cl_2) for major diastereomer;

^{1}H NMR (400 MHz, DMSO-*d*$_6$) δ (major diastereomer) 7.45 (dd, *J* = 8.6, 2.0 Hz, 1H), 7.42 – 7.37 (m, 3H), 7.37 – 7.31 (m, 2H), 7.24 (d, *J* = 2.4 Hz, 1H), 7.02 (s, 1H), 5.20 (d, *J* = 6.4 Hz, 1H), 4.84 (s, 1H), 4.64 (td, *J* = 10.6, 10.2, 5.4 Hz, 1H), 4.60 – 4.53 (m, 1H), 3.93 (d, *J* = 6.4 Hz, 1H), 2.94 (dd, *J* = 12.5, 5.1 Hz, 1H), 2.49 – 2.42 (m, 1H).

^{13}C NMR (101 MHz, DMSO-*d*$_6$) δ (major diastereomer) 173.6, 157.3, 133.8, 132.5, 128.9, 128.3, 128.1, 126.7, 122.8, 114.0, 111.7, 68.3, 68.1, 64.7, 60.1, 36.0.

HRMS (ESI-TOF) *m/z*: [M + H]$^+$ calcd for $C_{19}H_{16}{}^{79}BrN_2O_5$, 431.0237; found, 431.0242. [M + H]$^+$ calcd for $C_{19}H_{16}{}^{81}BrN_2O_5$, 433.0219; found, 433.0224.

(1*R*,3*R*,3a*S*,8b*R*)-7-methyl-3a-nitro-3-phenyl-2,3,3a,4',5',8b-hexahydro-2'*H*-spiro[benzofuro[2,3-*c*]pyrrole-1,3'-furan]-2'-one(5e)

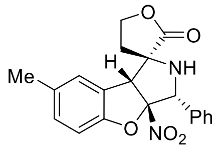

65.9 mg, 90% yield for the sum of the diastereomers, 60% yield for the major isomer, white soild, 67:33 dr, 97% ee, m.p.: 105.5-106.0 ℃; **The ee was determined by HPLC** (Chiralpak AD, *i*-PrOH/hexane = 10/90, flow rate 1.0 mL/min, λ = 220 nm, major diastereomer: t_{minor} = 13.3 min, t_{major} = 20.8 min); $[\alpha]_D^{20}$ = -89.3 (*c* 1.00, CH_2Cl_2) for major diastereomer;
^{1}H NMR (400 MHz, DMSO-d_6) δ (major diastereomer) 7.42 – 7.32 (m, 5H), 7.06 (d, *J* = 8.2 Hz, 1H), 6.91 – 6.83 (m, 2H), 5.21 (s, 1H), 4.79 (s, 1H), 4.61 (dd, *J* = 14.2, 7.0 Hz, 2H), 3.82 (s, 1H), 2.94 (dd, *J* = 12.4, 4.7 Hz, 1H), 2.48 – 2.40 (m, 1H), 2.23 (s, 3H).
^{13}C NMR (101 MHz, DMSO-d_6) δ (major diastereomer) 173.7, 156.1, 134.0, 131.8, 130.1, 128.8, 128.3, 128.0, 125.7, 123.9, 123.0, 109.2, 68.4, 68.1, 64.7, 60.7, 36.3, 20.4.
HRMS (ESI-TOF) *m/z*: [M + H]$^+$ calcd for $C_{20}H_{19}N_2O_5$, 367.1288; found, 367.1303.

(1*R*,3*R*,3aS,8b*R*)-7-(tert-butyl)-3a-nitro-3-phenyl-2,3,3a,4',5',8b-hexahydro-2'*H*-spiro[benzofuro[2,3-*c*]pyrrole-1,3'-furan]-2'-one(5f)

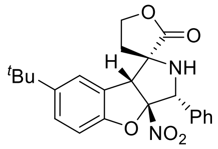

80.1 mg, 98% yield for the sum of the diastereomers, 86% yield for the major isomer, white soild, 88:12 dr, 93% ee, m.p.: 96.8-97.2 ℃; **The ee was determined by HPLC** (Chiralpak IA, *i*-PrOH/hexane = 15/85, flow rate 1.0 mL/min, λ = 254 nm, major diastereomer: t_{minor} = 7.7 min, t_{major} = 10.2 min); $[\alpha]_D^{20}$ = -218.8 (*c* 1.00, CH_2Cl_2) for major diastereomer;
^{1}H NMR (400 MHz, DMSO-d_6) δ (major diastereomer) 7.42 – 7.37 (m, 3H), 7.37 – 7.33 (m, 2H), 7.28 (d, *J* = 10.0 Hz, 1H), 7.07 (s, 1H), 6.90 (d, *J* = 8.5 Hz, 1H), 5.21 (d, *J* = 7.1 Hz, 1H), 4.80 (s, 1H), 4.68 (td, *J* = 10.3, 5.2 Hz, 1H), 4.58 (t, *J* = 8.8 Hz, 1H), 3.82 (d, *J* = 7.1 Hz, 1H), 2.96 (dd, *J* = 12.4, 5.1 Hz, 1H), 2.46 (dd, *J* = 11.6, 8.4 Hz, 1H), 1.23 (s, 9H).
^{13}C NMR (101 MHz, DMSO-d_6) δ (major diastereomer) 173.6, 155.8, 145.2, 134.0, 128.7, 128.3, 128.0, 126.4, 123.4, 123.0, 122.4, 108.7, 68.4, 68.3, 64.6, 60.9, 36.2, 34.1, 31.2.
HRMS (ESI-TOF) *m/z*: [M + H]$^+$ calcd for $C_{23}H_{25}N_2O_5$, 409.1758; found, 409.1767.

(1*R*,3*R*,3a*R*,8b*R*)-3-(4-fluorophenyl)-3a-nitro-2,3,3a,4',5',8b-hexahydro-2'*H*-spiro[benzofuro[2,3-*c*]pyrrole-1,3'-furan]-2'-one(5g)

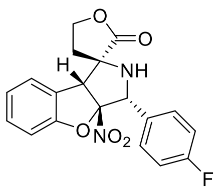

65.9 mg, 89% yield for the sum of the diastereomers, 64% yield for the major isomer, white soild, 72:28 dr, 80% ee, m.p.:147.6-148.2 ℃; **The ee was determined by HPLC** (Chiralpak AD, *i*-PrOH/hexane = 20/80, flow rate 1.0 mL/min, λ = 220 nm, major diastereomer: t_{minor} = 11.7 min, t_{major} = 9.7 min); $[\alpha]_D^{20}$ = -110.5 (*c* 1.00, CH_2Cl_2) for major diastereomer;

^{1}H NMR (400 MHz, DMSO-d_6) δ (major diastereomer) 7.45 – 7.40 (m, 2H), 7.25 (q, *J* = 9.3, 8.5 Hz, 3H), 7.07 (d, *J* = 7.2 Hz, 1H), 7.02 (dd, *J* = 11.5, 7.7 Hz, 2H), 5.24 (s, 1H), 4.86 (s, 1H), 4.66 – 4.54 (m, 2H), 3.82 (s, 1H), 2.95 (dd, *J* = 12.3, 4.9 Hz, 1H), 2.49 – 2.41 (m, 1H).

^{13}C NMR (101 MHz, DMSO-d_6) δ (major diastereomer) 173.5, 162.4 (d, *J* = 244.9 Hz), 157.9, 130.3, 130.0 (d, *J* = 49.8 Hz), 128.1 (d, *J* = 3.3 Hz), 125.5, 123.9, 122.9, 122.5, 115.1 (d, *J* = 21.5 Hz), 109.6, 68.3, 67.4, 64.6, 60.2, 36.1.

HRMS (ESI-TOF) *m/z*: $[M + Na]^+$ calcd for $C_{19}H_{15}FN_2O_5Na$, 393.0857; found, 393.0876.

(1*R*,3*R*,3a*R*,8b*R*)-3-(4-chlorophenyl)-3a-nitro-2,3,3a,4',5',8b-hexahydro-2'*H*-spiro[benzofuro[2,3-*c*]pyrrole-1,3'-furan]-2'-one(5h)

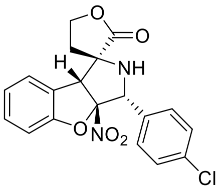

67.3 mg, 87% yield for the sum of the diastereomers, 85% yield for the major isomer, white soild, 98:2 dr, 87% ee, m.p.:200.2-200.8 ℃; **The ee was determined by HPLC** (Chiralpak IA, *i*-PrOH/hexane = 20/80, flow rate 1.0 mL/min, λ = 254 nm, major diastereomer: t_{minor} = 16.8 min, t_{major} = 11.6 min); $[\alpha]_D^{20}$ = -32.6 (*c* 1.00, CH_2Cl_2) for major diastereomer;

^{1}H NMR (400 MHz, DMSO-d_6) δ (major diastereomer) 7.48 (d, *J* = 8.7 Hz, 2H), 7.39 (d, *J* = 8.2 Hz, 2H), 7.29 – 7.23 (m, 1H), 7.09 – 6.97 (m, 3H), 5.24 (d, *J* = 6.3 Hz, 1H), 4.86 (s, 1H), 4.66 – 4.54 (m, 2H), 3.93 (d, *J* = 6.3 Hz, 1H), 2.94 (dd, *J* = 12.3, 4.8 Hz, 1H), 2.49 – 2.40 (m, 1H).

^{13}C NMR (101 MHz, DMSO-d_6) δ (major diastereomer) 173.5, 157.9, 133.5, 133.3, 130.0, 129.8, 128.3, 125.6, 123.8, 122.9, 122.4, 109.6, 68.3, 67.3, 64.6, 60.2, 36.1.

HRMS (ESI-TOF) *m/z*: $[M + H]^+$ calcd for $C_{19}H_{16}{}^{35}ClN_2O_5$, 387.0742; found, 387.0754. $[M + H]^+$ calcd for $C_{19}H_{16}{}^{37}ClN_2O_5$, 389.0721; found, 389.0728.

(1*R*,3*R*,3a*R*,8b*R*)-3a-nitro-3-(m-tolyl)-2,3,3a,4',5',8b-hexahydro-2'*H*-spiro[benzofuro[2,3-*c*]pyrrole-1,3'-furan]-2'-one(5i)

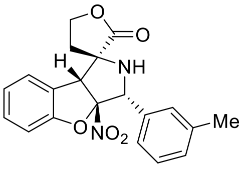

60.1 mg, 82% yield for the sum of the diastereomers, 53% yield for the major isomer, white soild, 65:35 dr, 90% ee, m.p.:227.6-228.1 ℃; **The ee was determined by HPLC** (Chiralpak AD, *i*-PrOH/hexane = 20/80, flow rate 1.0 mL/min, λ = 220 nm, major diastereomer: t_{minor} = 15.9 min, t_{major} = 7.8 min); $[\alpha]_D^{20}$ = -42.3 (*c* 1.00, CH_2Cl_2) for major diastereomer;
^{1}H NMR (400 MHz, DMSO-d_6) δ (major diastereomer) 7.27 (q, *J* = 8.2, 7.8 Hz, 2H), 7.20 (d, *J* = 7.7 Hz, 1H), 7.16 (s, 1H), 7.11 (d, *J* = 7.7 Hz, 1H), 7.06 (d, *J* = 7.3 Hz, 1H), 7.05 – 6.99 (m, 2H), 5.18 (d, *J* = 7.1 Hz, 1H), 4.84 (s, 1H), 4.68 – 4.55 (m, 2H), 3.79 (d, *J* = 7.2 Hz, 1H), 2.95 (dd, *J* = 12.4, 4.9 Hz, 1H), 2.44 (dd, *J* = 11.9, 8.7 Hz, 1H), 2.32 (s, 3H).
^{13}C NMR (101 MHz, DMSO-d_6) δ (major diastereomer) 173.6, 157.9, 137.5, 133.8, 129.7, 129.4, 128.4, 128.2, 125.5, 125.1, 123.9, 122.84, 122.75, 109.6, 68.4, 68.1, 64.7, 60.6, 36.2, 21.0.
HRMS (ESI-TOF) *m/z*: $[M + H]^+$ calcd for $C_{20}H_{19}N_2O_5$, 367.1288; found, 367.1298.

(1*R*,3*R*,3a*R*,8b*R*)-3-(4-isopropylphenyl)-3a-nitro-2,3,3a,4',5',8b-hexahydro-2'*H*-spiro[benzofuro[2,3-*c*]pyrrole-1,3'-furan]-2'-one(5j)

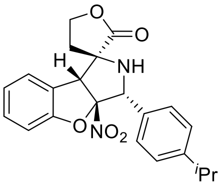

71.0 mg, 90% yield for the sum of the diastereomers, 73% yield for the major isomer, white soild, 81:19 dr, 93% ee, m.p.:144.9-145.3 ℃; **The ee was determined by HPLC** (Chiralpak IA, *i*-PrOH/hexane = 20/80, flow rate 1.0 mL/min, λ = 254 nm, major diastereomer: t_{minor} = 16.7 min, t_{major} = 9.0 min); $[\alpha]_D^{20}$ = -88.8 (*c* 1.00, CH_2Cl_2) for major diastereomer;
^{1}H NMR (400 MHz, DMSO-d_6) δ (major diastereomer) 7.27 (s, 5H), 7.07 (d, *J* = 7.5 Hz, 1H), 7.03 (dd, *J* = 7.9, 5.4 Hz, 2H), 5.18 (s, 1H), 4.85 (s, 1H), 4.68 – 4.54 (m, 2H), 3.76 (s, 1H), 2.99 – 2.87 (m, 2H), 2.48 – 2.40 (m, 1H), 1.22 (d, *J* = 6.9 Hz, 6H).
^{13}C NMR (101 MHz, DMSO-d_6) δ (major diastereomer) 173.6, 157.9, 149.0, 131.2, 129.7, 128.1, 126.2, 125.5, 123.9, 122.84, 122.79, 109.6, 68.4, 68.1, 64.7, 60.6, 36.2, 33.2, 23.8, 23.8.
HRMS (ESI-TOF) *m/z*: $[M + H]^+$ calcd for $C_{22}H_{23}N_2O_5$, 395.1601; found, 395.1601.

(1*R*,3*R*,3a*R*,8b*R*)-3-(2,4-dimethylphenyl)-3a-nitro-2,3,3a,4',5',8b-hexahydro-2'*H*-spiro[benzofuro[2,3-*c*]pyrrole-1,3'-furan]-2'-one(5k)

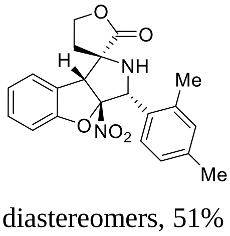

44.1 mg, 58% yield for the sum of the diastereomers, 51% yield for the major isomer, white soild, 88:12 dr, 70% ee, m.p.:229.8-230.2 ℃; **The ee was determined by HPLC** (Chiralpak AD, *i*-PrOH/hexane = 20/80, flow rate 1.0 mL/min, λ = 220 nm, major diastereomer: t_{minor} = 27.2 min, t_{major} = 8.5 min); $[\alpha]_D^{20}$ = -41.9 (*c* 1.00, CH_2Cl_2) for major diastereomer;
^{1}H NMR (400 MHz, DMSO-d_6) δ (major diastereomer) 7.26 (d, *J* = 7.2 Hz, 1H), 7.15 (d, *J* = 7.8 Hz, 1H), 7.11 – 7.08 (m, 1H), 7.08 – 6.98 (m, 4H), 5.14 (d, *J* = 7.4 Hz, 1H), 4.83 (s, 1H), 4.68 –

4.54 (m, 2H), 3.72 (d, *J* = 7.5 Hz, 1H), 2.98 – 2.90 (m, 1H), 2.48 – 2.40 (m, 1H), 2.23 (d, *J* = 3.4 Hz, 6H).
^{13}C NMR (101 MHz, DMSO-*d*$_6$) δ (major diastereomer) 173.7, 157.9, 136.8, 136.1, 131.1, 129.7, 129.4, 128.8, 125.5, 125.3, 123.8, 122.9, 122.8, 109.6, 68.4, 68.1, 64.7, 60.7, 36.3, 19.4, 19.1.
HRMS (ESI-TOF) *m/z*: $[M + H]^+$ calcd for $C_{21}H_{21}N_2O_5$, 381.1445; found, 381.1446.

(1*R*,3*S*,3a*S*,8b*S*)-3-(naphthalen-2-yl)-3a-nitro-2,3,3a,4',5',8b-hexahydro-2'*H*-spiro[benzofuro [2,3-*c*]pyrrole-1,3'-furan]-2'-one(5l)

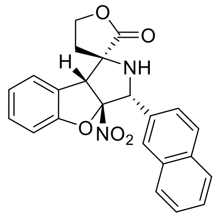

52.3 mg, 65% yield for the sum of the diastereomers, 64% yield for the major isomer, white soild, 92:8 dr, 82% ee, m.p.:210.4-210.8 ℃; **The ee was determined by HPLC** (Chiralpak IA, *i*-PrOH/hexane = 20/80, flow rate 1.0 mL/min, λ = 220 nm, major diastereomer: t_{minor} = 32.7 min, t_{major} = 13.5 min); $[\alpha]_D^{20}$ = -48.7 (*c* 1.00, CH_2Cl_2) for major diastereomer;
^{1}H NMR (400 MHz, DMSO-*d*$_6$) δ 7.96 (dd, *J* = 9.3, 4.4 Hz, 4H), 7.59 – 7.52 (m, 2H), 7.47 (d, *J* = 8.7 Hz, 1H), 7.30 – 7.23 (m, 1H), 7.10 (d, *J* = 7.4 Hz, 1H), 7.07 – 7.01 (m, 1H), 6.99 (d, *J* = 8.1 Hz, 1H), 5.43 (d, *J* = 6.7 Hz, 1H), 4.91 (s, 1H), 4.71 – 4.58 (m, 2H), 4.01 (d, *J* = 6.9 Hz, 1H), 3.02 (dd, *J* = 12.3, 4.9 Hz, 1H), 2.54 (dd, *J* = 11.6, 8.6 Hz, 1H).
^{13}C NMR (101 MHz, DMSO-*d*$_6$) δ 173.6, 158.0, 133.1, 132.6, 131.7, 129.8, 128.0, 127.8, 127.5, 127.2, 126.5, 126.4, 125.61, 125.57, 123.8, 122.9, 122.8, 109.6, 68.5, 68.3, 64.7, 60.6, 36.2.
HRMS (ESI-TOF) *m/z*: $[M + H]^+$ calcd for $C_{23}H_{19}N_2O_5$, 403.1288; found, 403.1294.

(1*R*,3*R*,3a*R*,8b*R*)-3-(furan-3-yl)-3a-nitro-2,3,3a,4',5',8b-hexahydro-2'*H*-spiro[benzofuro[2,3-c]pyrrole-1,3'-furan]-2'-one(5m)

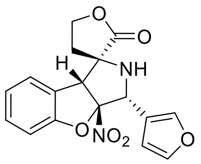

37.7 mg, 55% yield for the sum of the diastereomers, 36% yield for the major isomer, white soild, 66:34 dr, 91% ee, m.p.:199.8-200.3 ℃; **The ee was determined by HPLC** (Chiralpak AD-H, EtOH/hexane = 20/80 , flow rate 1.0 mL/min, λ = 254 nm, major diastereomer: t_{minor} = 12.0 min, t_{major} = 18.1 min); $[\alpha]_D^{20}$ = -56.9 (*c* 1.00, CH_2Cl_2) for major diastereomer;
^{1}H NMR (400 MHz, DMSO-*d*$_6$) δ (major diastereomer) 7.81 (s, 1H), 7.75 – 7.67 (m, 1H), 7.34 – 7.24 (m, 1H), 7.11 – 7.00 (m, 3H), 6.54 (d, *J* = 1.9 Hz, 1H), 5.12 (d, *J* = 9.8 Hz, 1H), 4.86 (s, 1H), 4.67 – 4.56 (m, 2H), 3.64 (d, *J* = 9.8 Hz, 1H), 2.94 – 2.86 (m, 1H), 2.43 (td, *J* = 11.6, 8.7 Hz, 1H).
^{13}C NMR (101 MHz, DMSO-*d*$_6$) δ (major diastereomer) 173.5, 157.9, 143.7, 141.9, 129.8, 125.5, 123.8, 123.2, 123.0, 118.2, 110.3, 109.8, 69.0, 65.0, 61.7, 61.1, 36.5.
HRMS (ESI-TOF) *m/z*: $[M + H]^+$ calcd for $C_{17}H_{15}N_2O_6$, 343.0925; found, 343.0931.

4. Characterization data for compounds 6-9

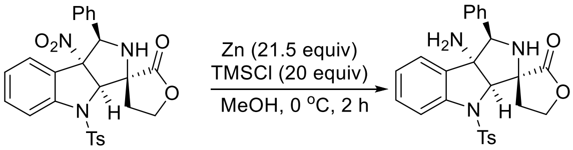

3a, >20:1 dr
96% ee

6, 83% yield
>20:1 dr, 93% ee

(1'*R*,3*S*,3a'*S*,8b'*R*)-8b'-amino-1'-phenyl-4'-tosyl-1',3a',4,4',5,8b'-hexahydro-2*H*,2'*H*-spiro[furan-3,3'-pyrrolo[3,4-*b*]indol]-2-one (6).

39.5 mg, 83% yield, white soild, >20:1 dr, 93% ee, m.p.:120.7-121.1 ℃; **The ee was determined by HPLC** (Chiralpak IA, *i*-PrOH/hexane = 20/80, flow rate 1.0 mL/min, λ = 254 nm, major diastereomer: t_{minor} = 25.1 min, t_{major} = 28.4 min); $[\alpha]_D^{20}$ = +25.6 (*c* 1.00, CH_2Cl_2) for major diastereomer;

^{1}H NMR (400 MHz, DMSO-d_6) δ 7.57 (d, *J* = 7.0 Hz, 3H), 7.42 (d, *J* = 8.1 Hz, 1H), 7.23 (dd, *J* = 8.1, 3.5 Hz, 5H), 7.19 – 7.13 (m, 3H), 6.60 – 6.53 (m, 1H), 5.43 (d, *J* = 7.6 Hz, 1H), 5.00 (s, 1H), 4.95 (d, *J* = 4.7 Hz, 1H), 4.55 (q, *J* = 7.2 Hz, 1H), 4.41 (q, *J* = 7.6 Hz, 1H), 4.33 (s, 1H), 3.95 (d, *J* = 4.9 Hz, 1H), 2.93 (dt, *J* = 13.8, 7.2 Hz, 1H), 2.27 (s, 3H), 2.22 (dt, *J* = 13.3, 6.5 Hz, 1H).

^{13}C NMR (101 MHz, DMSO-d_6) δ 178.4, 144.4, 143.0, 138.4, 133.2, 130.5, 129.5, 129.2, 127.9, 127.6, 127.4, 127.3, 126.6, 123.2, 114.8, 80.2, 71.0, 66.1, 65.9, 61.5, 32.6, 21.0.

HRMS (ESI-TOF) *m/z*: $[M + H]^+$ calcd for $C_{26}H_{26}N_3O_4S$, 476.1639; found, 476.1633.

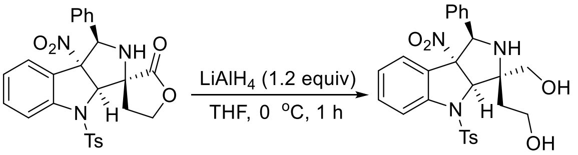

3a, >20:1 dr
96% ee

7, 58% yield
52:48 dr, 99% ee, 94% ee

2-(3-(hydroxymethyl)-8b-nitro-1-phenyl-4-tosyl-1,2,3,3a,4,8b-hexahydropyrrolo[3,4-*b*]indol-3-yl)ethan-1-ol(7)

29.6 mg, 58% yield, white soild, 52:48 dr, 99%, 94% ee, m.p.:130.1-130.5 ℃; **The ee was determined by HPLC** (Chiralpak AD-H, EtOH/hexane = 20/80, flow rate 1.0 mL/min, λ = 254 nm, major diastereomer: t_{minor} = 16.7 min, t_{major} = 9.0 min); $[\alpha]_D^{20}$ = +21.3 (*c* 1.00, CH_2Cl_2) for major diastereomer;

^{1}H NMR (400 MHz, DMSO-d_6) δ 7.73 (d, *J* = 7.9 Hz, 1H), 7.61 (dd, *J* = 20.5, 7.8 Hz, 3H), 7.45 (d, *J* = 8.1 Hz, 2H), 7.41 – 7.38 (m, 2H), 7.38 – 7.32 (m, 8H), 7.30 – 7.18 (m, 7H), 7.08 – 7.00 (m, 2H), 6.80 – 6.69 (m, 1H), 5.69 (s, 1H), 5.64 (d, *J* = 7.6 Hz, 1H), 5.43 (t, 1H), 5.35 (s, 1H), 5.31 (t, 1H), 5.23 (d, *J* = 6.1 Hz, 1H), 4.57 (d, *J* = 12.4 Hz, 1H), 4.48 (t, *J* = 5.0 Hz, 1H), 4.36 (t, *J* = 5.1 Hz, 1H), 3.87 (d, *J* = 6.0 Hz, 2H), 3.73 (d, *J* = 5.5 Hz, 2H), 3.64 – 3.55 (m, 2H), 3.53 (d, *J* = 13.1 Hz, 1H), 3.47 – 3.41 (m, 2H), 2.27 (s, 3H), 2.23 (s, 3H), 2.16 – 2.07 (m, 1H), 1.87 – 1.76 (m, 1H), 1.61 – 1.50 (m, 1H), 1.29 – 1.25 (m, 1H).

^{13}C NMR (101 MHz, DMSO-d_6) δ 144.8, 144.7, 143.9, 142.7, 136.6, 134.9, 132.8, 132.3, 131.6, 129.9, 129.8, 128.9, 128.8, 128.6, 128.43, 128.35, 128.3, 128.2, 127.8, 127.7, 127.3, 127.2, 126.8, 125.9, 124.9, 123.7, 117.1, 115.9, 105.7, 102.1, 75.5, 73.2, 71.6, 68.5, 67.8, 66.0, 65.4, 61.4, 57.2, 56.7, 37.5, 31.4, 21.0, 20.9.

HRMS (ESI-TOF) *m/z*: $[M + H]^+$ calcd for $C_{26}H_{25}N_2O_4S$, 510.1693; found, 510.1688.

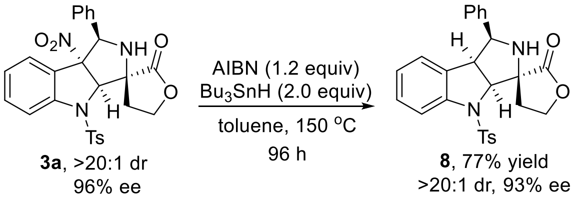

(1'*S*,3*S*,3a'*S*,8b'*S*)-1'-phenyl-4'-tosyl-1',3a',4,4',5,8b'-hexahydro-2*H*,2'*H*-spiro[furan-3,3'-pyrrolo[3,4-*b*]indol]-2-one (8)

35.5 mg, 77% yield, white soild, >20:1 dr, 93% ee, m.p.:220.1-220.4 ℃; **The ee was determined by HPLC** (Chiralpak IC, EtOH/hexane = 20/80, flow rate 1.0 mL/min, λ = 254 nm, major diastereomer: t_{minor} = 10.0 min, t_{major} = 10.7 min); $[\alpha]_D^{20}$ = +24.3 (*c* 1.00, CH_2Cl_2) for major diastereomer;

^{1}H NMR (400 MHz, DMSO-d_6) δ 7.51 – 7.42 (m, 3H), 7.30 – 7.22 (m, 5H), 7.18 (s, 2H), 7.14 – 7.08 (m, 1H), 6.63 – 6.53 (m, 1H), 5.39 (d, *J* = 7.6 Hz, 1H), 4.95 (t, *J* = 6.0 Hz, 1H), 4.85 (d, *J* = 8.2 Hz, 1H), 4.52 (td, *J* = 8.4, 4.3 Hz, 1H), 4.42 (q, *J* = 8.4, 7.8 Hz, 1H), 4.02 (d, *J* = 5.6 Hz, 1H), 3.94 (t, *J* = 7.4 Hz, 1H), 2.89 (dt, *J* = 13.4, 8.0 Hz, 1H), 2.29 (s, 3H), 2.26 – 2.16 (m, 1H).

^{13}C NMR (101 MHz, DMSO-d_6) δ 178.6, 144.7, 142.4, 138.3, 133.2, 131.4, 130.3, 130.0, 128.0, 127.9, 127.4, 127.1, 126.0, 124.0, 115.8, 69.9, 67.8, 66.4, 61.7, 50.4, 32.5, 21.0.

HRMS (ESI-TOF) *m/z*: $[M + H]^+$ calcd for $C_{26}H_{25}N_2O_4S$, 461.1530; found, 461.1540.

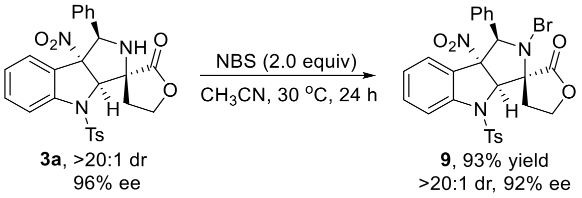

(1'*R*,3*S*,3a'*S*,8b'*S*)-2'-bromo-8b'-nitro-1'-phenyl-4'-tosyl-1',3a',4,4',5,8b'-hexahydro-2*H*,2'*H*-spiro[furan-3,3'-pyrrolo[3,4-*b*]indol]-2-one (9)

54.4 mg, 93% yield, white soild, >20:1 yield, 92% ee; m.p.:153.4-153.8 ℃; **The ee was determined by HPLC** (Chiralpak IC, EtOH/hexane = 20/80, flow rate 1.0 mL/min, λ = 254 nm, major diastereomer: t_{minor} = 7.1 min, t_{major} = 7.9 min); $[\alpha]_D^{20}$ = +34.5 (*c* 1.00, CH_2Cl_2) for major diastereomer;

^{1}H NMR (400 MHz, DMSO-d_6) δ 7.68 (d, *J* = 8.2 Hz, 1H), 7.47 (dd, *J* = 14.0, 8.0 Hz, 3H), 7.39 – 7.33 (m, 1H), 7.31 – 7.23 (m, 4H), 7.05 (d, *J* = 7.6 Hz, 2H), 6.87 – 6.79 (m, 1H), 5.78 (d, *J* = 7.8 Hz, 1H), 5.50 (s, 1H), 5.33 (s, 1H), 4.58 (td, *J* = 8.5, 4.0 Hz, 1H), 4.45 (q, *J* = 8.0 Hz, 1H), 3.03 (dt, *J* = 13.6, 8.2 Hz, 1H), 2.35 – 2.28 (m, 1H), 2.27 (s, 3H).

^{13}C NMR (101 MHz, DMSO-d_6) δ177.1, 145.4, 143.5, 134.7, 132.2, 131.8, 130.0, 129.0, 128.6, 127.8, 127.6, 127.5, 124.4, 124.3, 116.2, 101.1, 73.9, 66.9, 66.6, 66.5, 31.6, 21.0.

HRMS (ESI-TOF) *m/z*: $[M + H]^+$ calcd for $C_{26}H_{23}{}^{79}BrN_3O_6S$, 584.0485; found, 584.0492. $[M + H]^+$ calcd for $C_{26}H_{23}{}^{81}BrN_3O_6S$, 586.0468; found, 586.0472.

5. X-ray crystal structure of compounds 3a and 5c

Single crystal of compound **3a** was prepared from the mixture solvent of ethyl acetate/petroleum ether (1/2) at room temperature by slow evaporation of solvent. A suitable crystal was selected for structure determination on a XtaLAB AFC12 (RINC): Kappa single diffractometer. The crystal was kept at 150.00(10) K during data collection. Using Olex2 [1], the

structure was solved with the SHELXT [2] structure solution program using Intrinsic Phasing and refined with the SHELXL [3] refinement package using Least Squares minimisation.

[1] Dolomanov, O. V.; Bourhis, L. J.; Gildea, R. J, Howard, J. A. K.; Puschmann, H. *J. Appl. Cryst*. **2009**, *42*, 339-341.

[2] Sheldrick, G. M. *Acta Cryst.* **2008**, A64, 112-122.

[3] Sheldrick, G. M. *Acta Cryst*. **2015**, C71, 3-8.

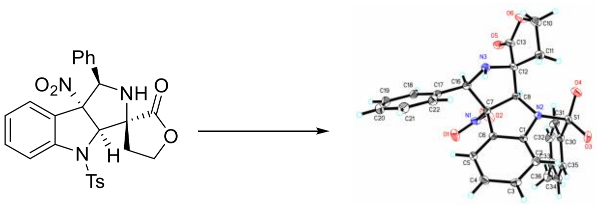

3a **3a**, CCDC-2178034

Table 1 Crystal data and structure refinement for 3a.

Identification code	**3a**
Empirical formula	$C_{26}H_{23}N_3O_6S$
Formula weight	505.53
Temperature/K	150.00(10)
Crystal system	orthorhombic
Space group	$P2_12_12_1$
a/Å	9.1955(2)
b/Å	12.9582(3)
c/Å	19.8231(5)
α/°	90
β/°	90
γ/°	90
Volume/Å^3	2362.06(10)
Z	4
ρ_{calc}g/cm^3	1.422
μ/mm^{-1}	1.637
F(000)	1056.0
Crystal size/mm^3	0.14 × 0.11 × 0.09
Radiation	Cu Kα (λ = 1.54184)
2Θ range for data collection/°	8.152 to 144.48
Index ranges	$-11 \le h \le 10$, $-15 \le k \le 12$, $-21 \le l \le 24$
Reflections collected	10706
Independent reflections	4529 [R_{int} = 0.0301, R_{sigma} = 0.0279]
Data/restraints/parameters	4529/0/330
Goodness-of-fit on F^2	1.045
Final R indexes [I>=2σ (I)]	R_1 = 0.0426, wR_2 = 0.1115
Final R indexes [all data]	R_1 = 0.0431, wR_2 = 0.1120
Largest diff. peak/hole / e Å^{-3}	0.45/-0.41

Flack/Hooft parameter	-0.005(14)/-0.005(6)

Single crystal of compound **5c** was prepared from the mixture solvent of dichloromethane/EtOH (1/5) at room temperature by slow evaporation of solvent.

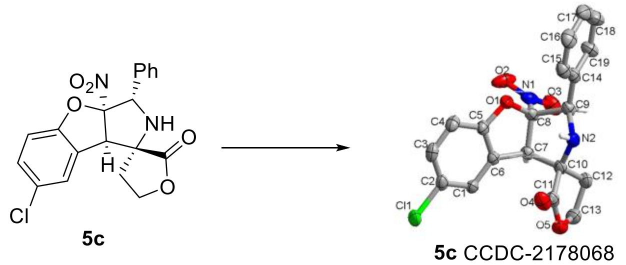

5c CCDC-2178068

Table 1 Crystal data and structure refinement for 5c.

Identification code	**5c**
Empirical formula	$C_{19}H_{15}ClN_2O_5$
Formula weight	386.78
Temperature/K	293(2)
Crystal system	orthorhombic
Space group	$P2_12_12_1$
a/Å	9.9074(6)
b/Å	13.3728(8)
c/Å	13.4556(9)
α/°	90
β/°	90
γ/°	90
Volume/Å^3	1782.7(2)
Z	4
ρ_{calc}g/cm^3	1.441
μ/mm^{-1}	2.204
F(000)	800.0
Crystal size/mm^3	0.15 × 0.1 × 0.09
Radiation	CuKα (λ = 1.54184)
2Θ range for data collection/°	9.324 to 144.02
Index ranges	$-12 \le h \le 11$, $-16 \le k \le 16$, $-15 \le l \le 16$
Reflections collected	13126
Independent reflections	3409 [R_{int} = 0.0373, R_{sigma} = 0.0308]
Data/restraints/parameters	3409/1/259
Goodness-of-fit on F^2	1.056
Final R indexes [I>=2σ (I)]	R_1 = 0.0444, wR_2 = 0.1191
Final R indexes [all data]	R_1 = 0.0528, wR_2 = 0.1288
Largest diff. peak/hole / e Å^{-3}	0.31/-0.30
Flack parameter	-0.014(13)

6. Unsuccessful examples

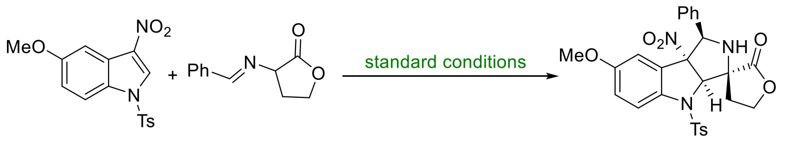

trace

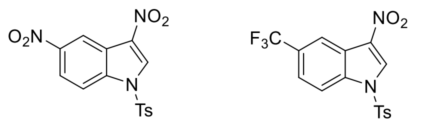

These two substrates are not available at present

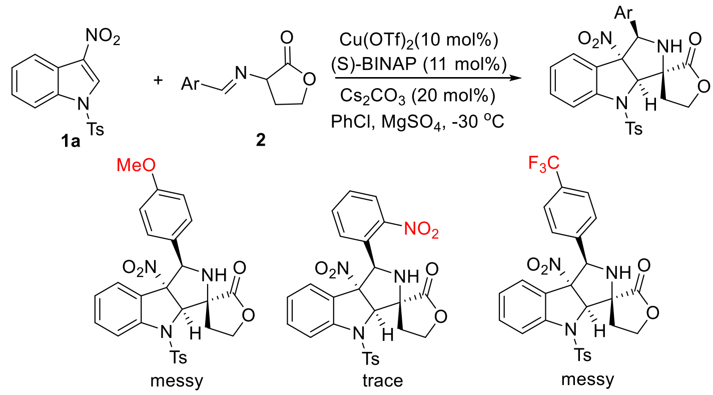

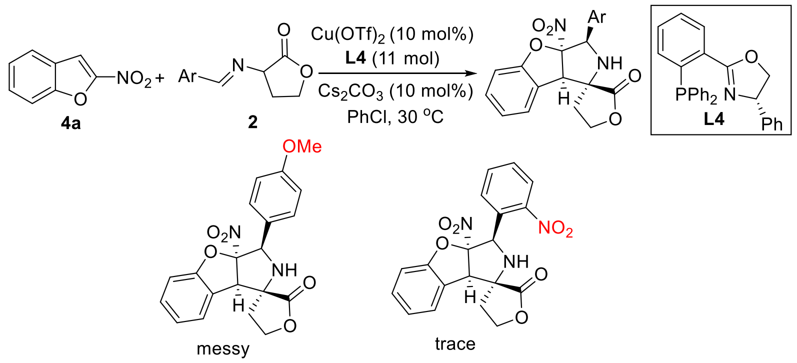

7. ^{1}H NMR, ^{13}C NMR and HPLC spectra for compounds 3,5 and 6-9

^{1}H NMR (400 MHz, DMSO-d_6) and ^{13}C NMR (101 MHz, DMSO-d_6) of 3a

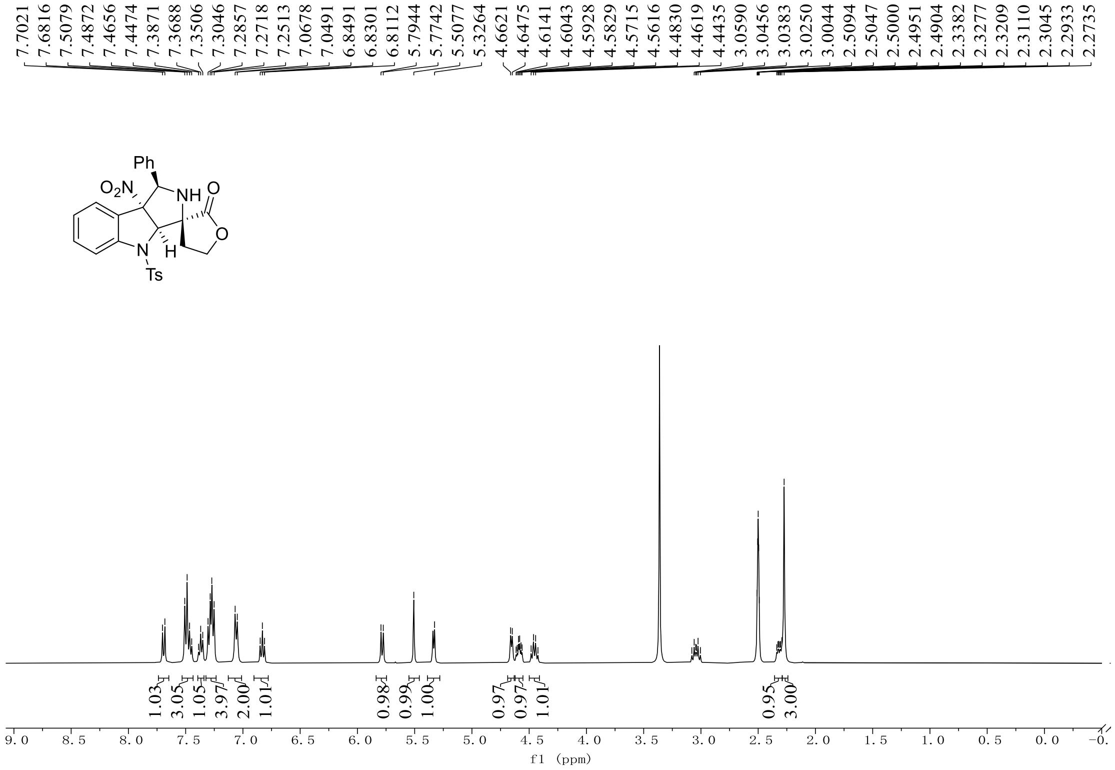

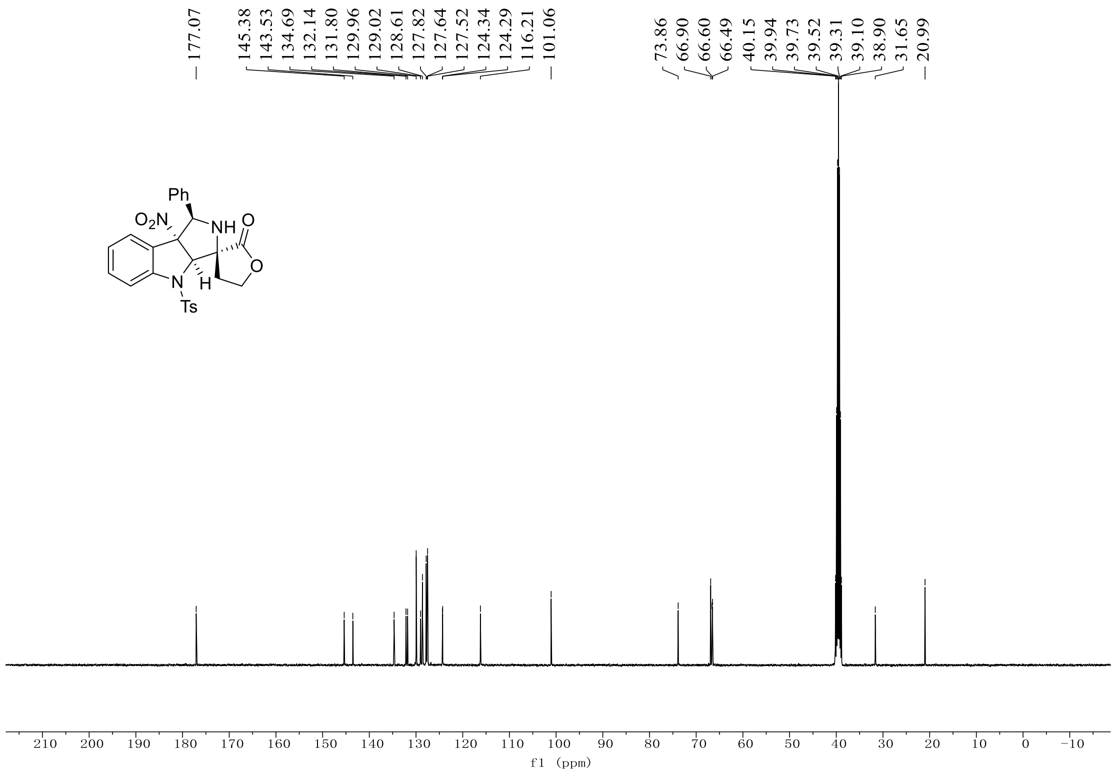

HPLC spectra of 3a

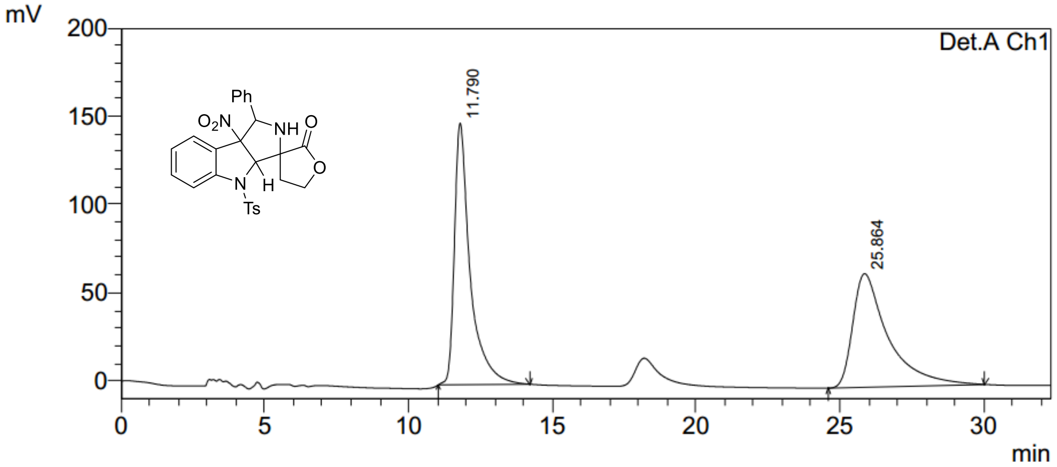

1 Det.A Ch1/254nm

PeakTable

Detector A Ch1 254nm

Peak#	Ret. Time	Area	Height	Area %
1	11.790	5624264	148086	50.483
2	25.864	5516663	64333	49.517
Total		11140927		100.000

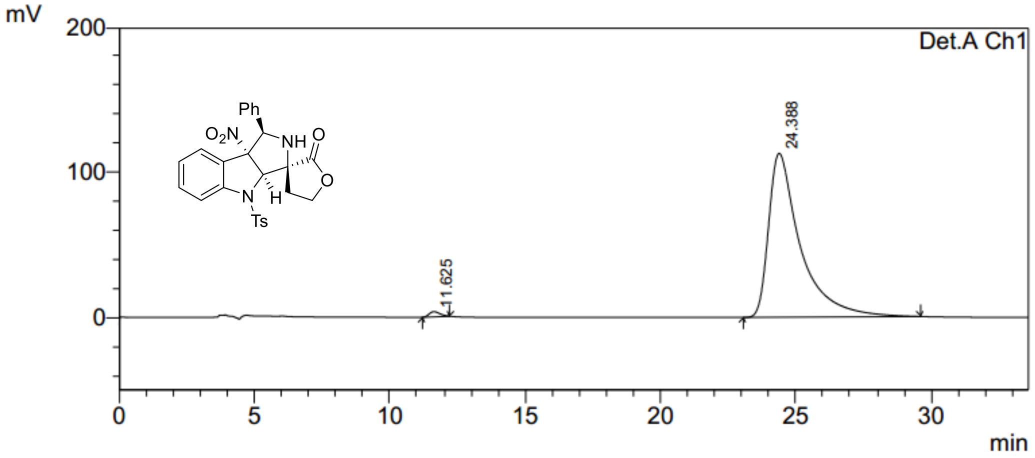

1 Det.A Ch1/254nm

PeakTable

Detector A Ch1 254nm

Peak#	Ret. Time	Area	Height	Area %
1	11.625	100348	3610	1.076
2	24.388	9229724	113106	98.924
Total		9330072		100.000

^{1}H NMR (400 MHz, DMSO-d_6) and ^{13}C NMR (101 MHz, DMSO-d_6) of 3b

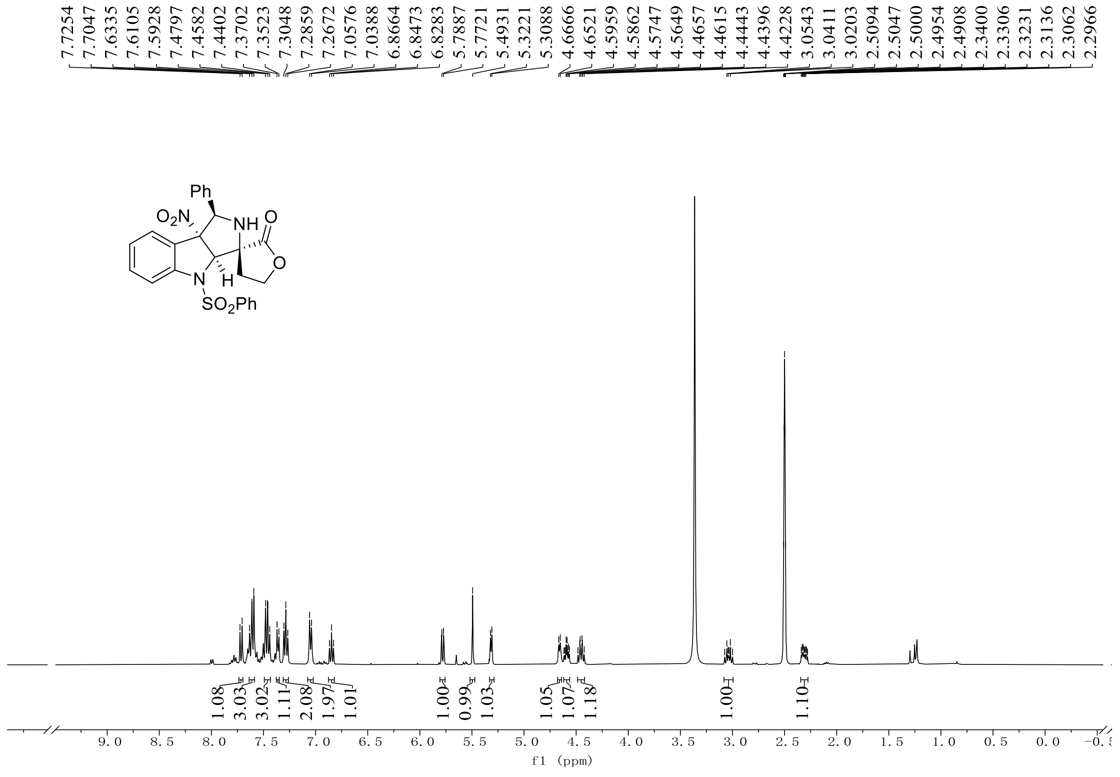

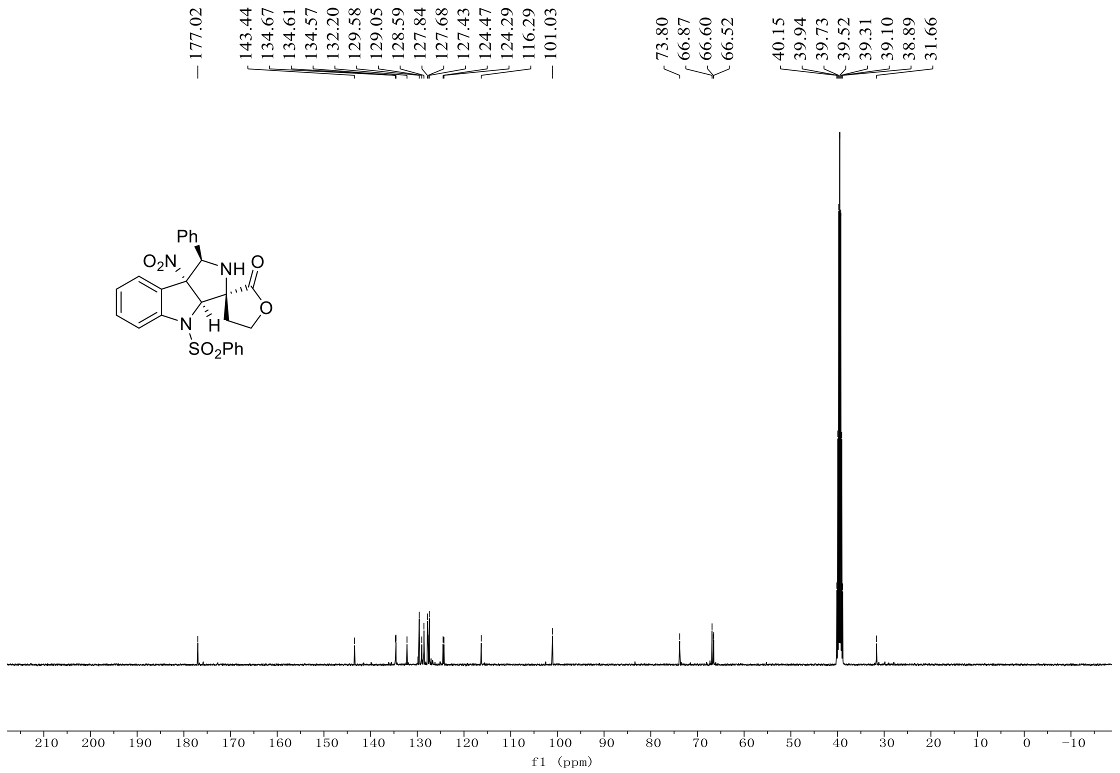

HPLC spectra of 3b

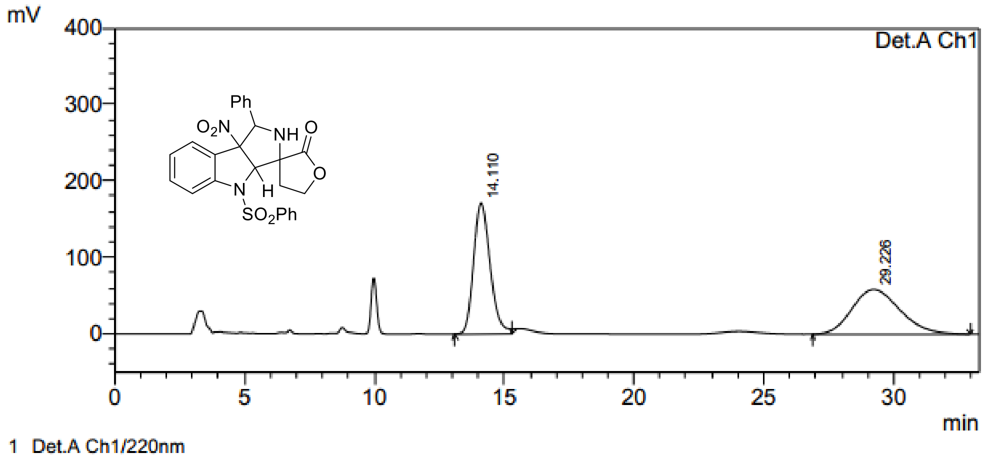

PeakTable

Detector A Ch1 220nm

Peak#	Ret. Time	Area	Height	Area %
1	14.110	7664788	171374	50.085
2	29.226	7638637	58684	49.915
Total		15303425		100.000

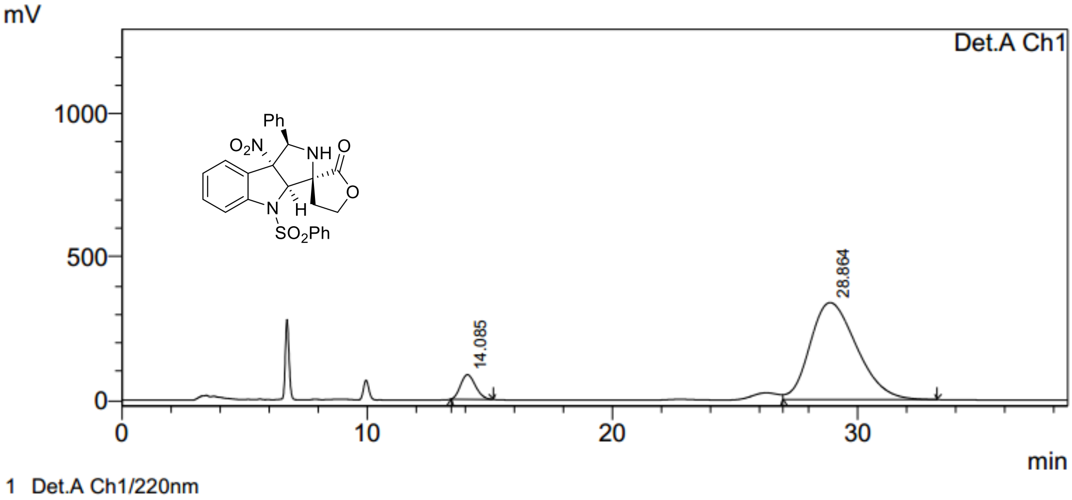

PeakTable

Detector A Ch1 220nm

Peak#	Ret. Time	Area	Height	Area %
1	14.085	3658641	86526	7.513
2	28.864	45036347	339465	92.487
Total		48694988		100.000

^{1}H NMR (400 MHz, DMSO-d_6) and ^{13}C NMR (101 MHz, DMSO-d_6) of 3c

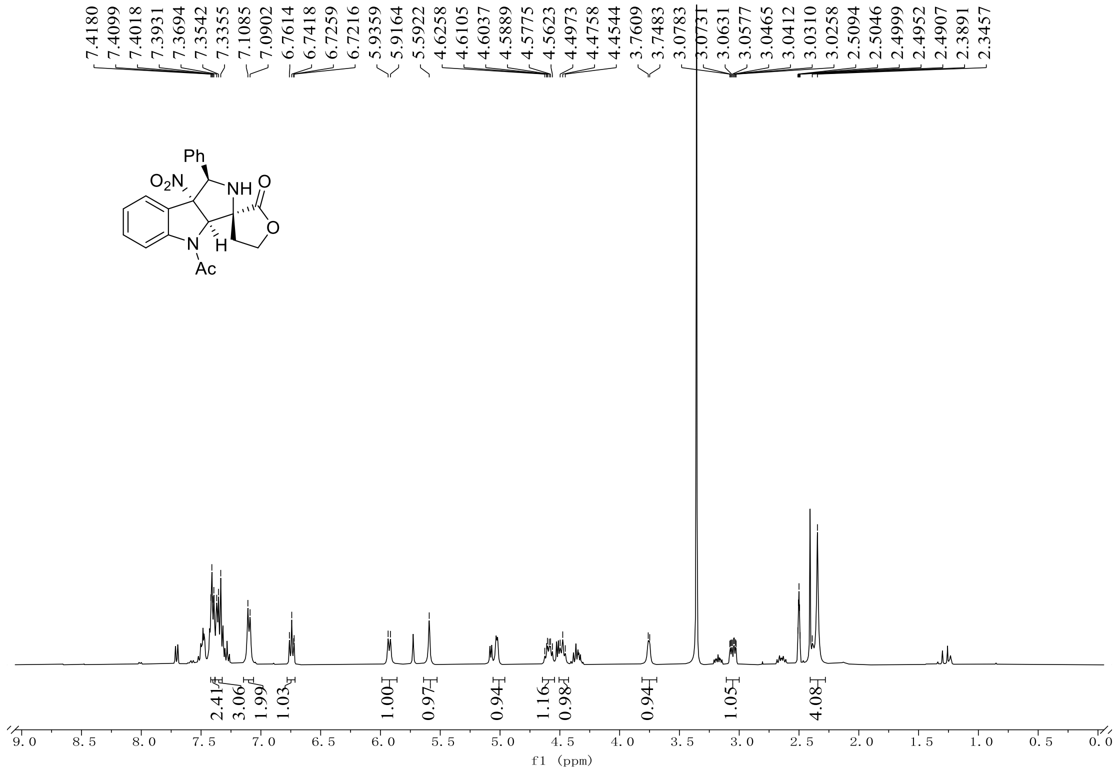

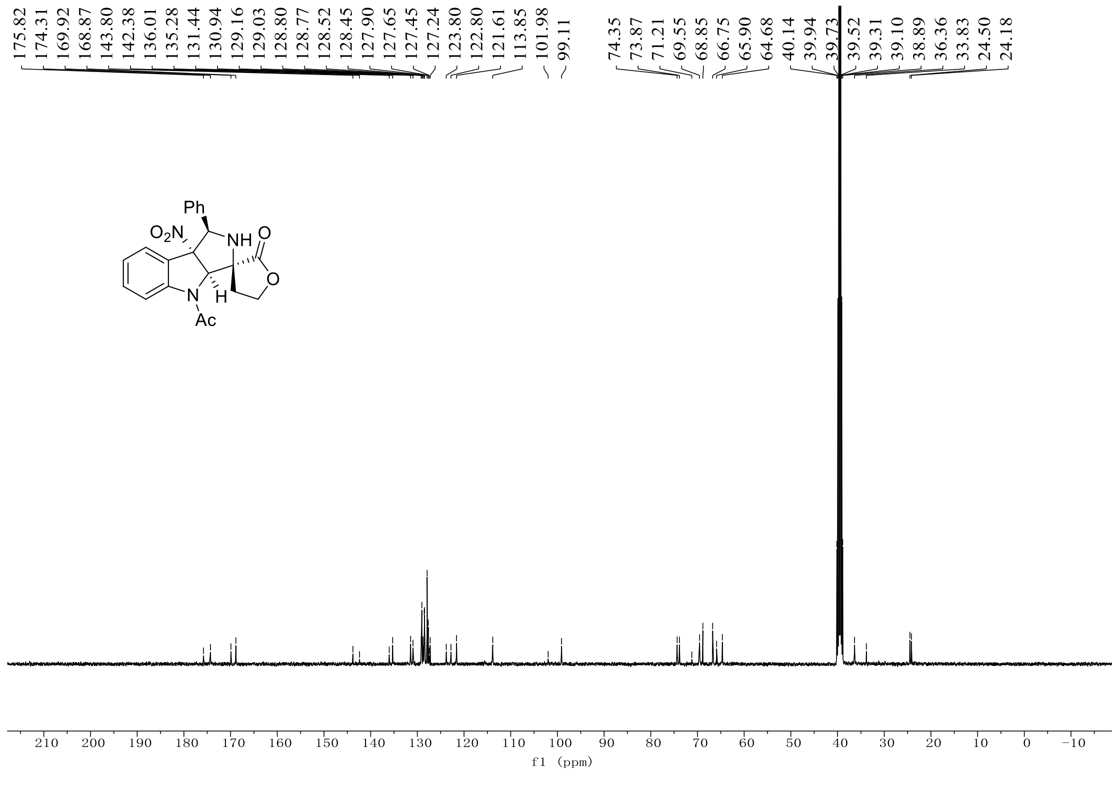

HPLC spectra of 3c

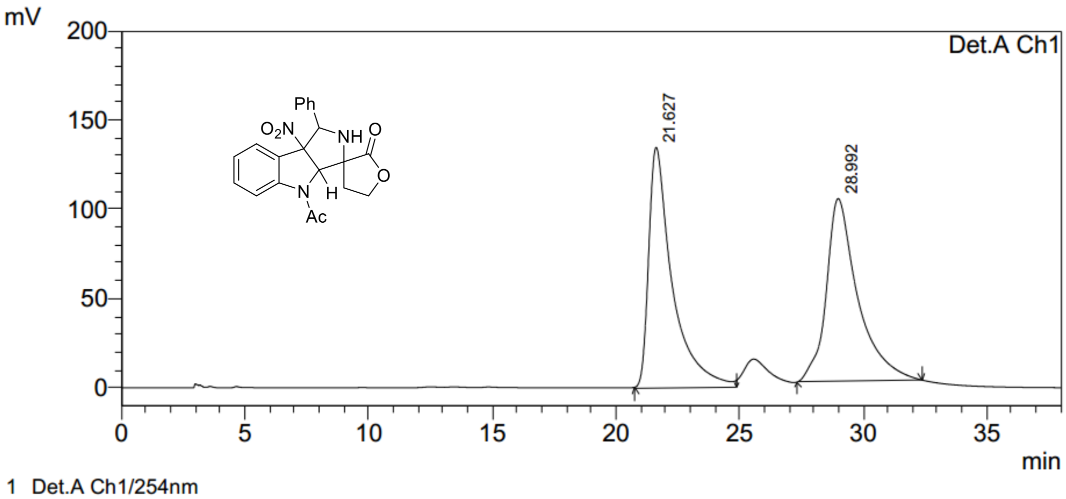

1 Det.A Ch1/254nm

PeakTable

Detector A Ch1 254nm

Peak#	Ret. Time	Area	Height	Area %
1	21.627	8980846	134713	49.803
2	28.992	9051775	102136	50.197
Total		18032621		100.000

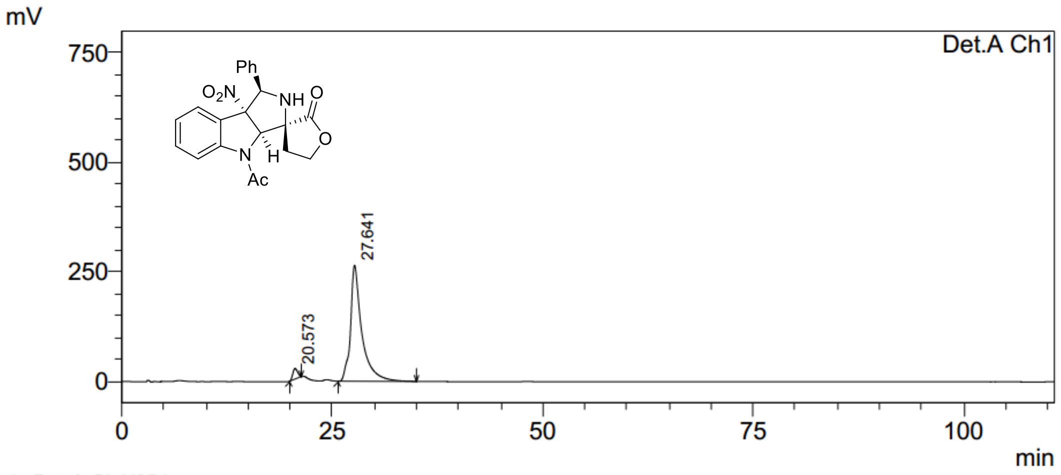

1 Det.A Ch1/254nm

PeakTable

Detector A Ch1 254nm

Peak#	Ret. Time	Area	Height	Area %
1	20.573	958003	24408	3.705
2	27.641	24896678	264828	96.295
Total		25854682		100.000

^{1}H NMR (400 MHz, DMSO-d_6) and ^{13}C NMR (101 MHz, DMSO-d_6) of 3d

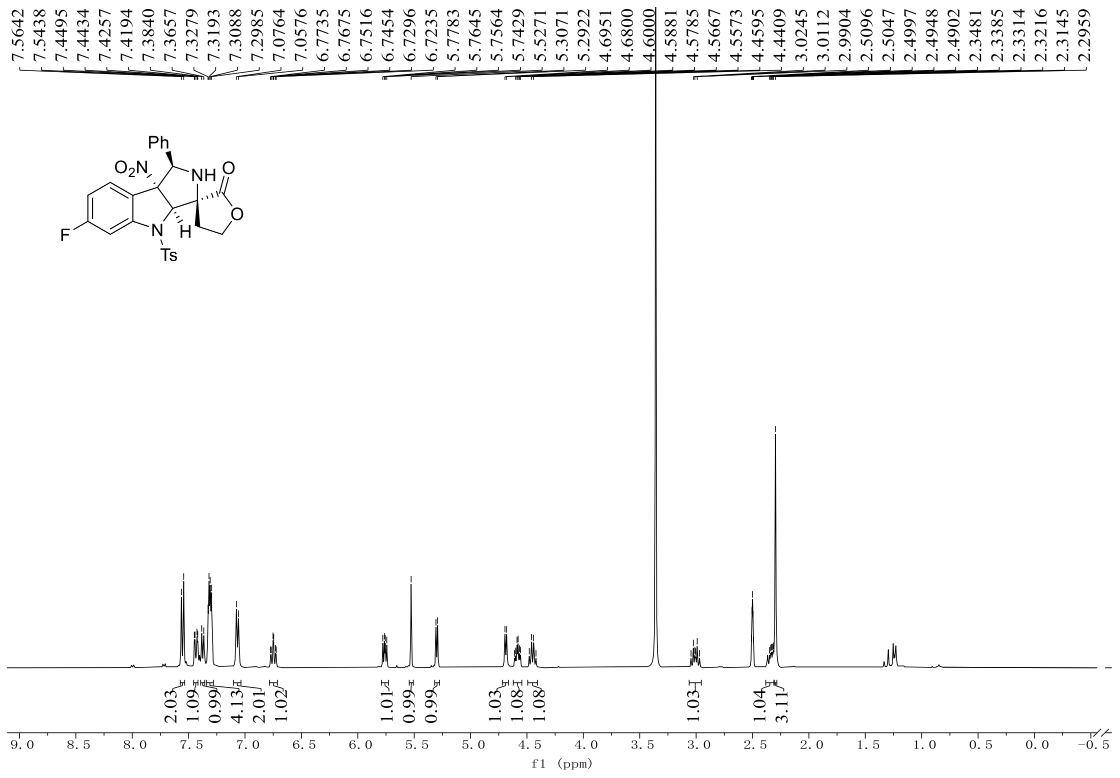

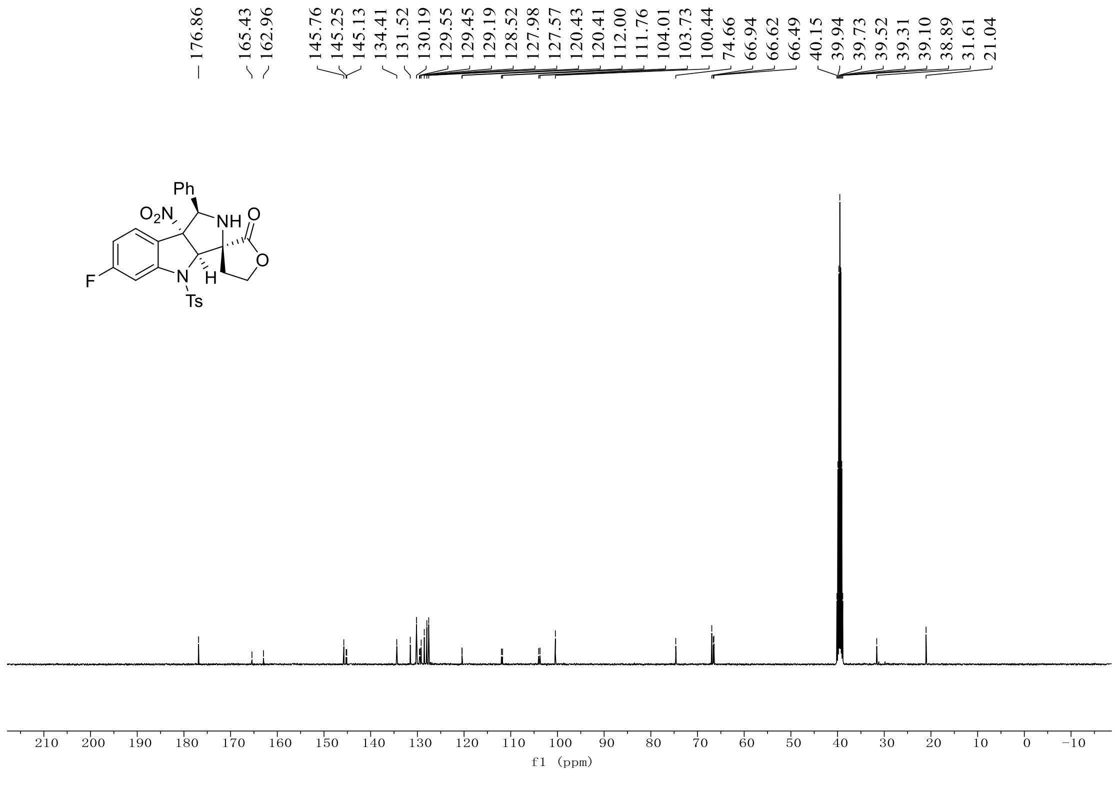

HPLC spectra of 3d

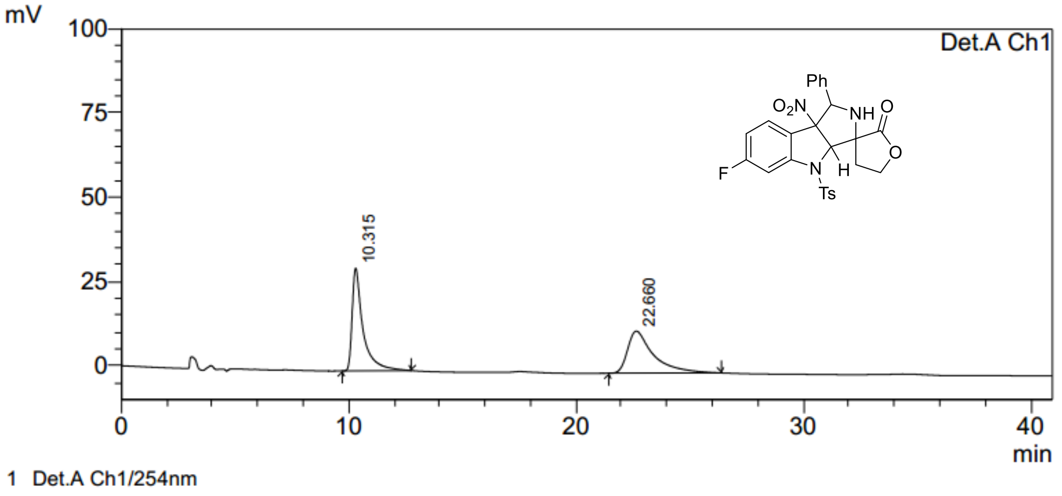

PeakTable

Detector A Ch1 254nm

Peak#	Ret. Time	Area	Height	Area %
1	10.315	1016807	30426	50.776
2	22.660	985716	12448	49.224
Total		2002522		100.000

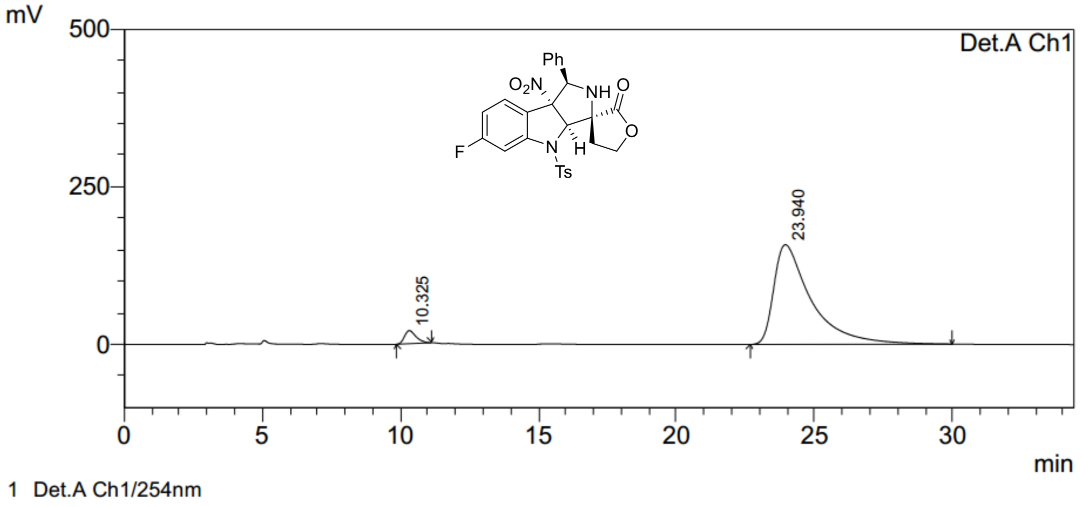

PeakTable

Detector A Ch1 254nm

Peak#	Ret. Time	Area	Height	Area %
1	10.325	604156	20761	3.893
2	23.940	14916849	157860	96.107
Total		15521005		100.000

^{1}H NMR (400 MHz, DMSO-d_6) and ^{13}C NMR (101 MHz, DMSO-d_6) of 3e

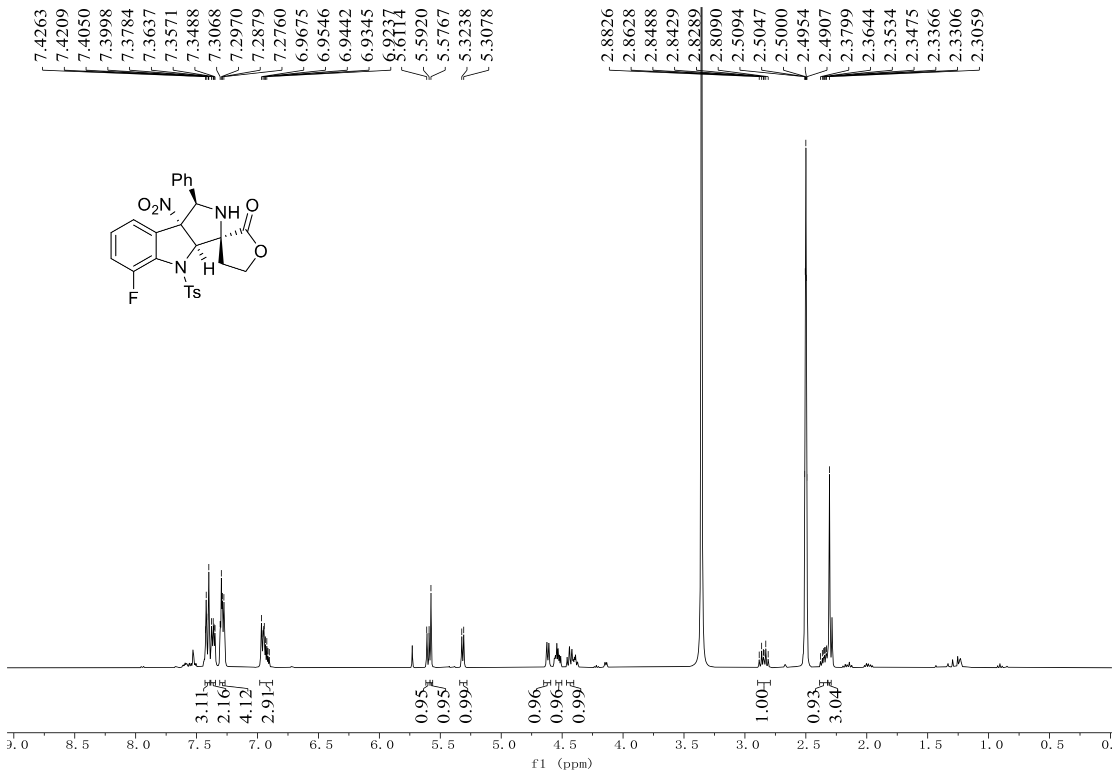

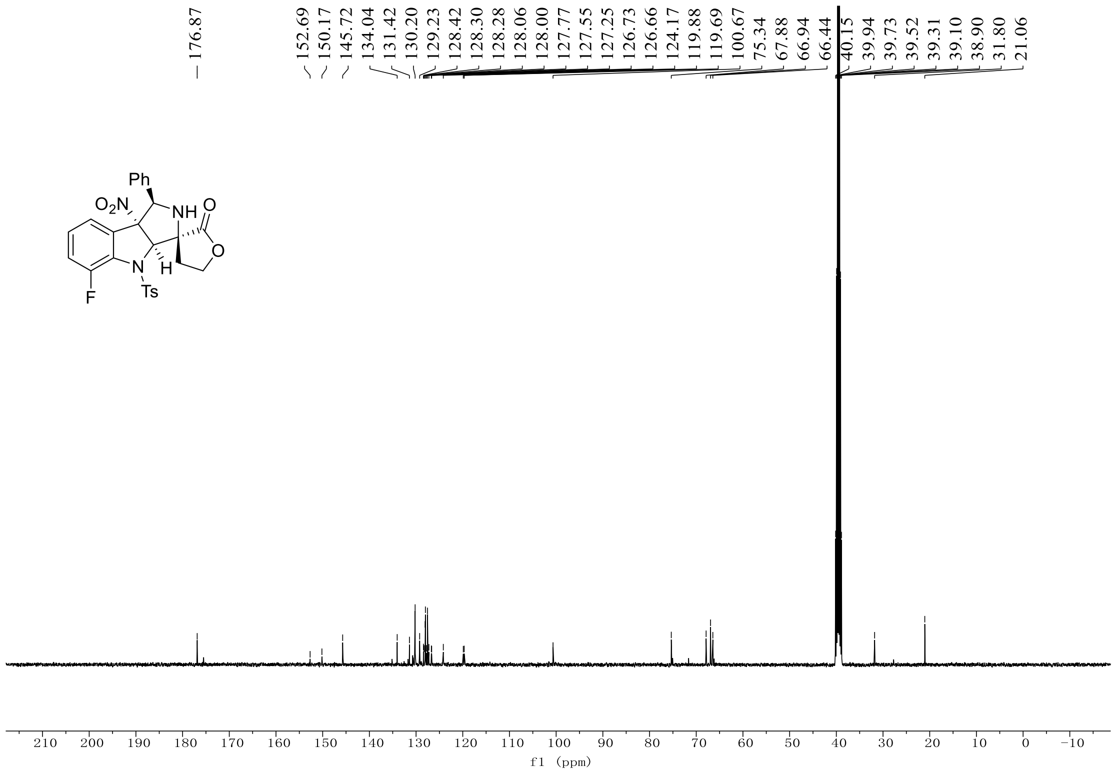

HPLC spectra of 3e

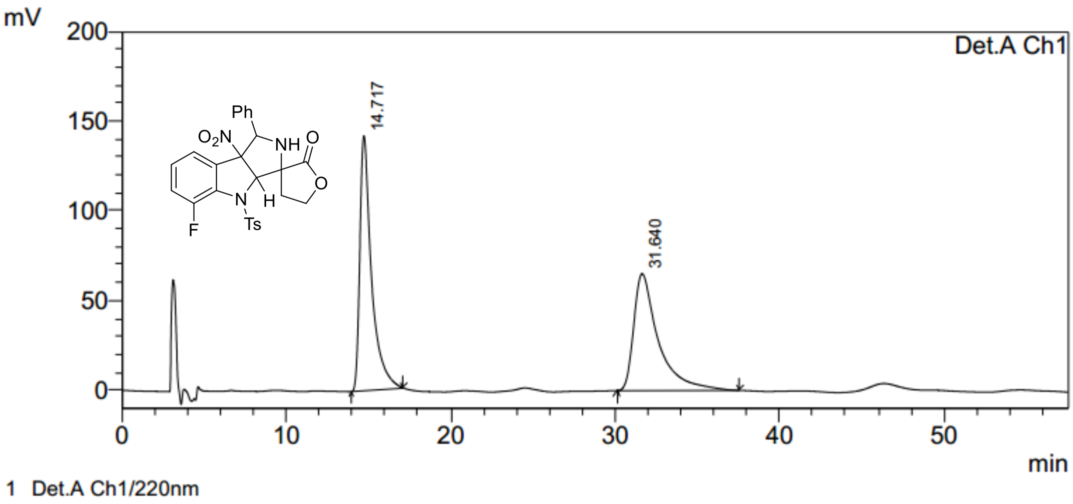

PeakTable

Detector A Ch1 220nm

Peak#	Ret. Time	Area	Height	Area %
1	14.717	7019346	142067	50.829
2	31.640	6790249	65175	49.171
Total		13809594		100.000

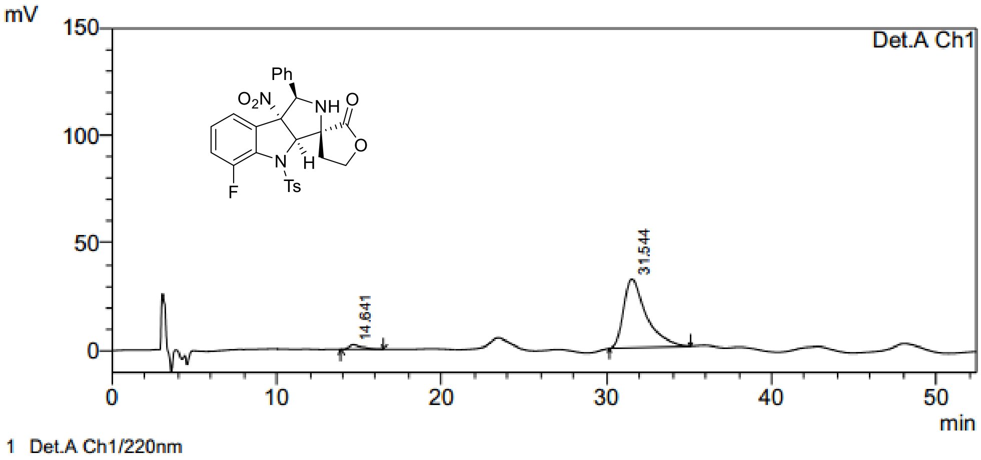

PeakTable

Detector A Ch1 220nm

Peak#	Ret. Time	Area	Height	Area %
1	14.641	113057	2153	3.546
2	31.544	3075118	32006	96.454
Total		3188175		100.000

^{1}H NMR (400 MHz, DMSO-d_6) and ^{13}C NMR (101 MHz, DMSO-d_6) of 3f

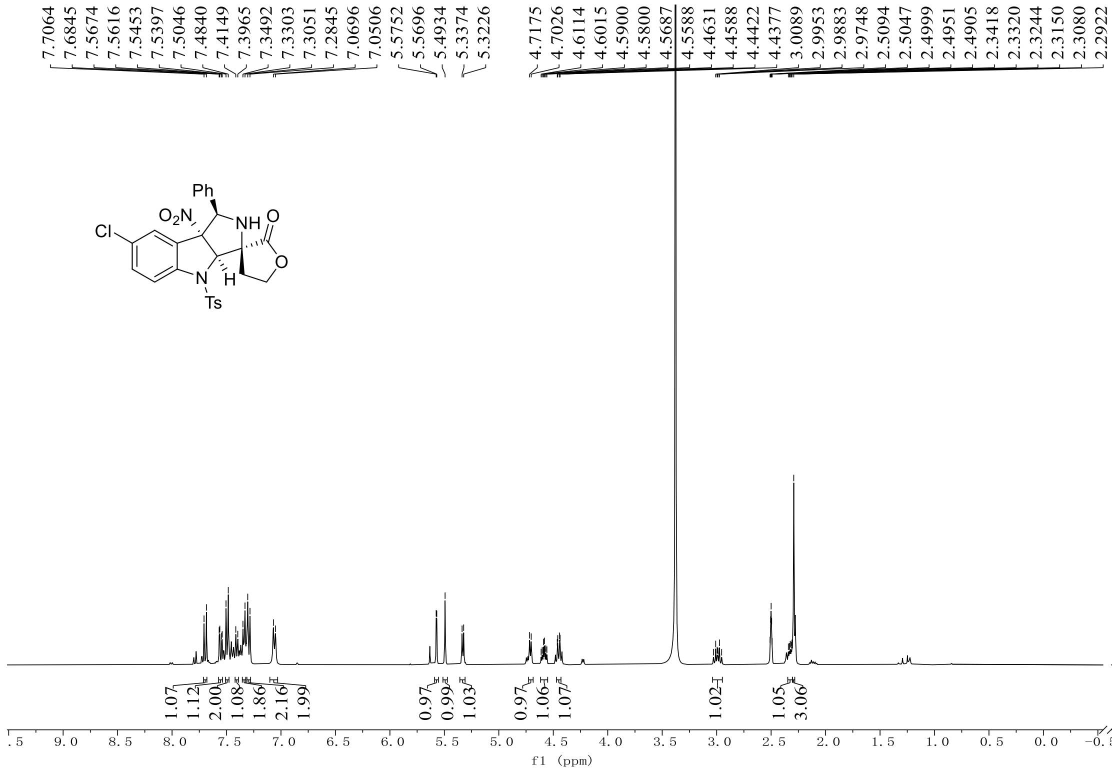

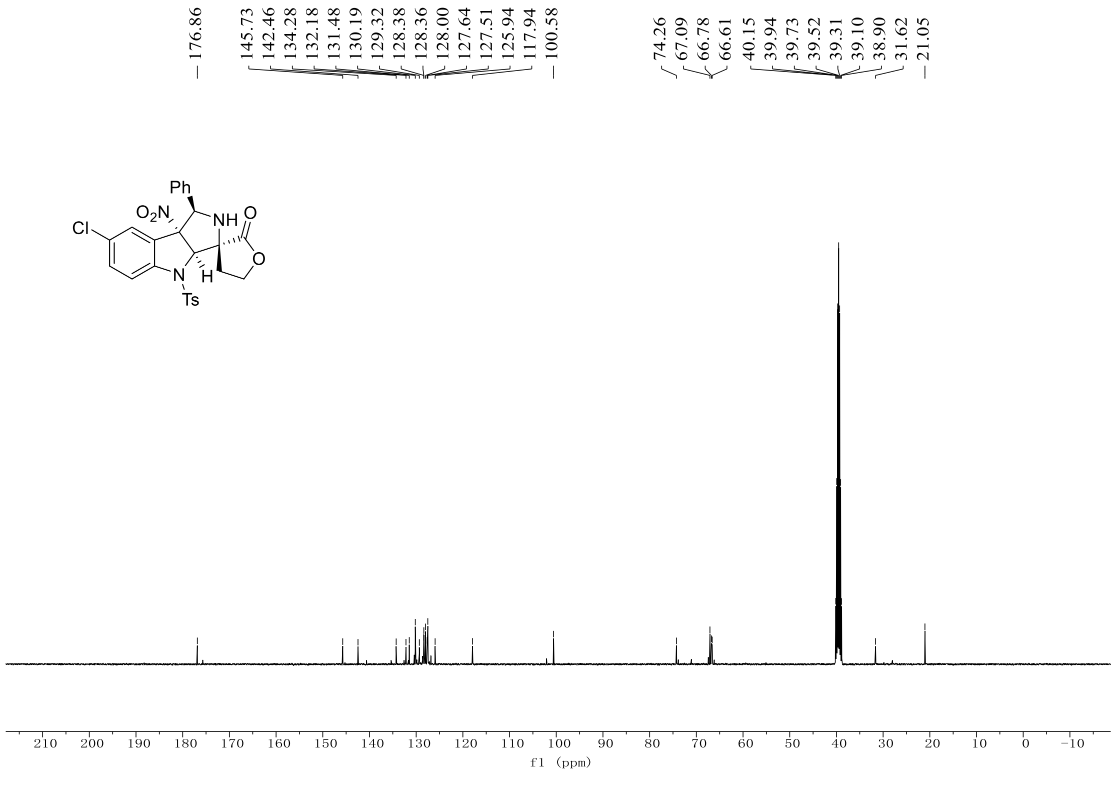

HPLC spectra of 3f

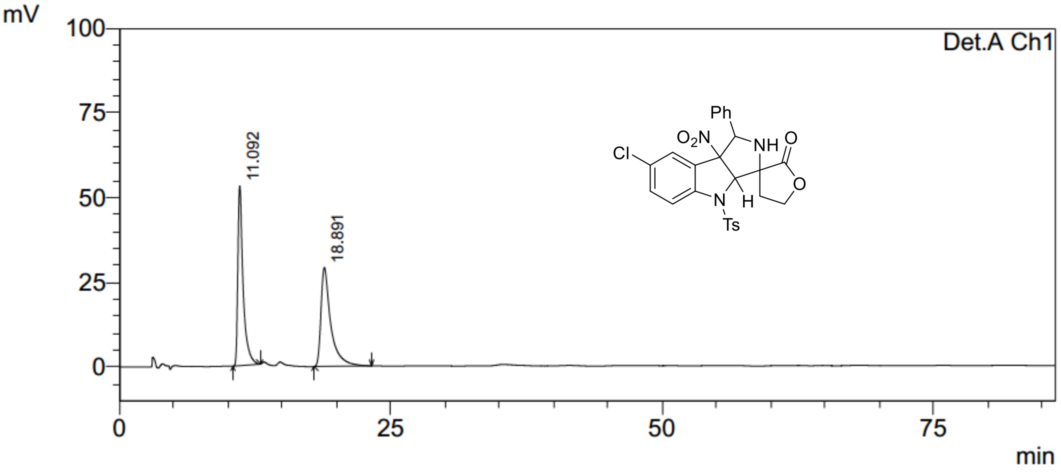

1 Det.A Ch1/254nm

PeakTable

Detector A Ch1 254nm

Peak#	Ret. Time	Area	Height	Area %
1	11.092	1787960	53036	49.422
2	18.891	1829766	29162	50.578
Total		3617726		100.000

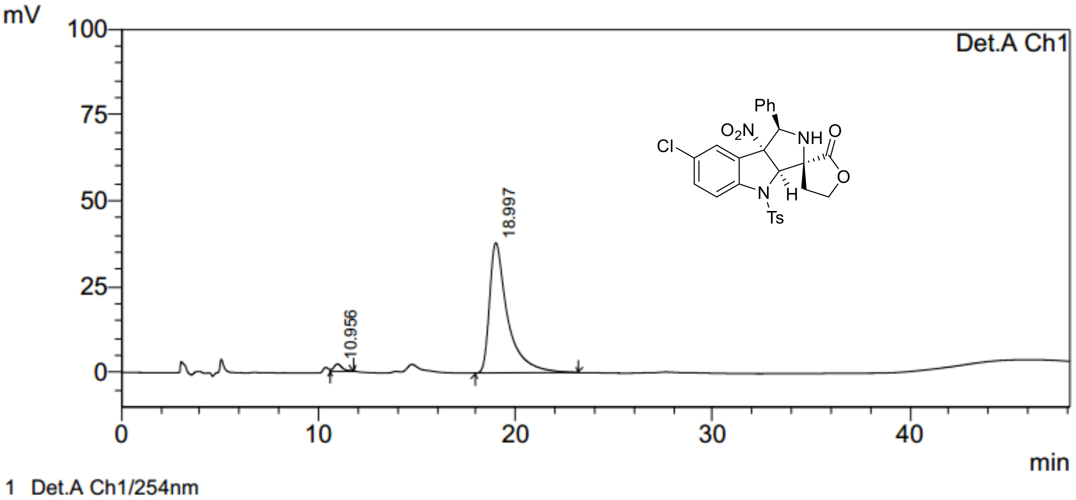

1 Det.A Ch1/254nm

PeakTable

Detector A Ch1 254nm

Peak#	Ret. Time	Area	Height	Area %
1	10.956	63225	2136	2.539
2	18.997	2427051	37896	97.461
Total		2490276		100.000

^{1}H NMR (400 MHz, DMSO-d_6) and ^{13}C NMR (101 MHz, DMSO-d_6) of 3g

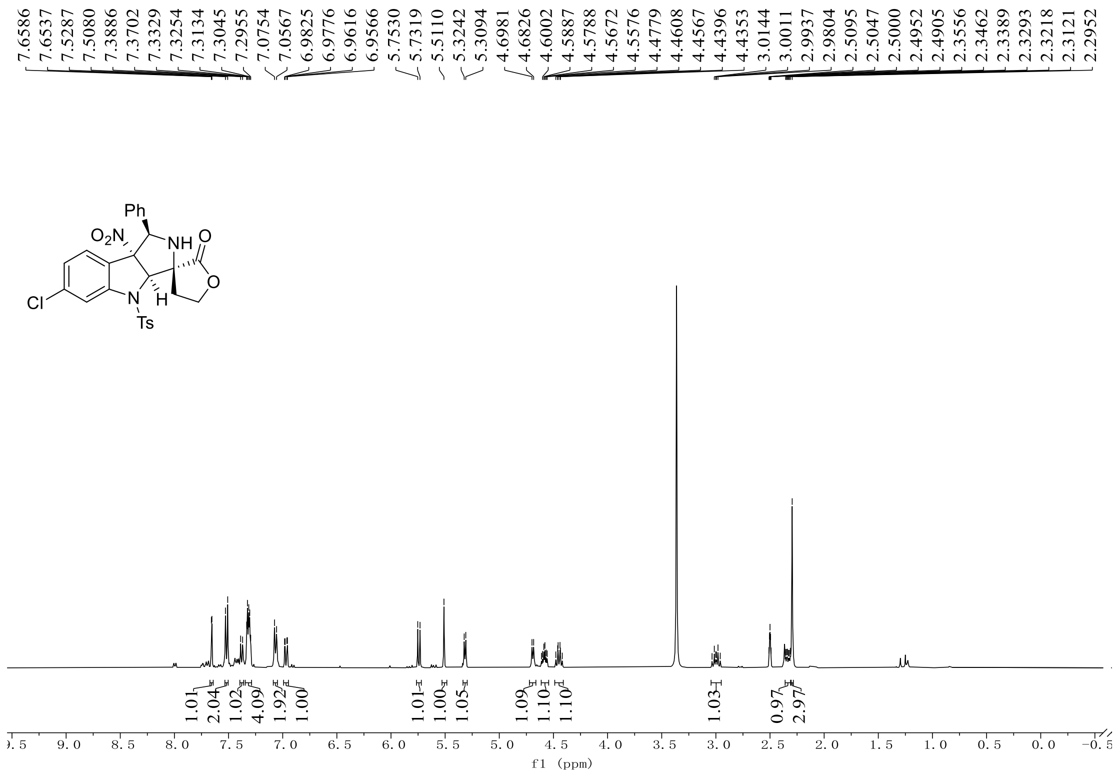

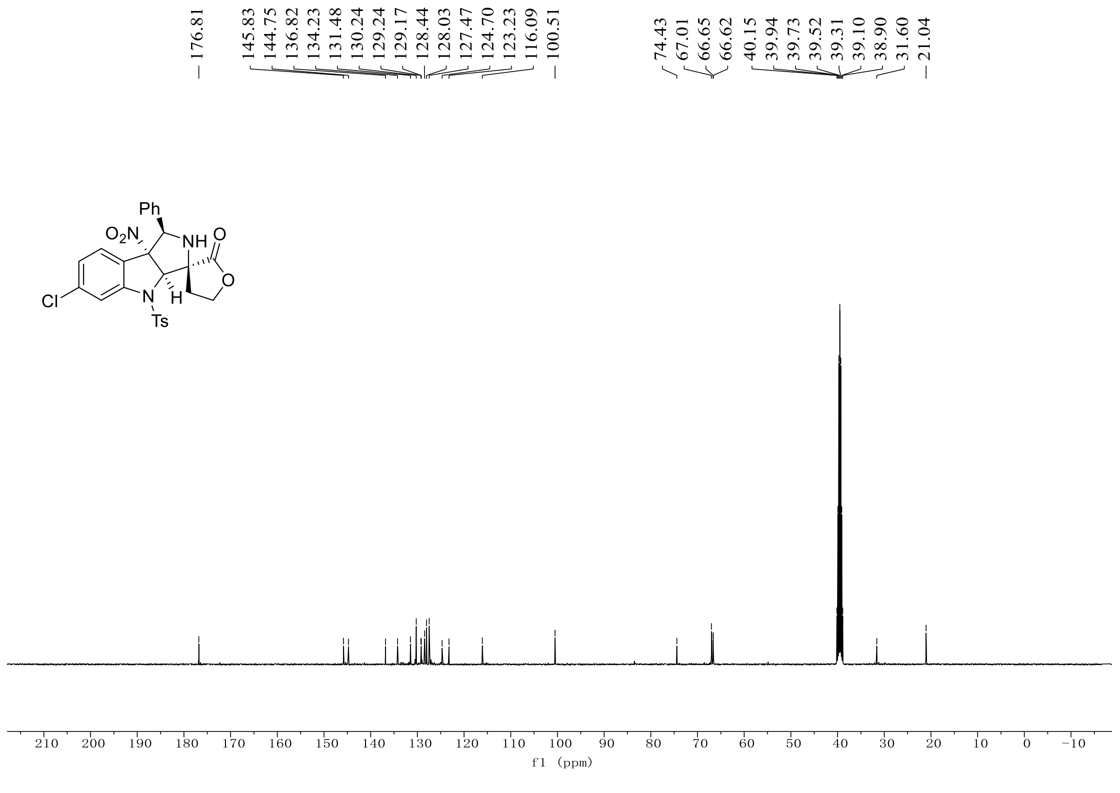

HPLC spectra of 3g

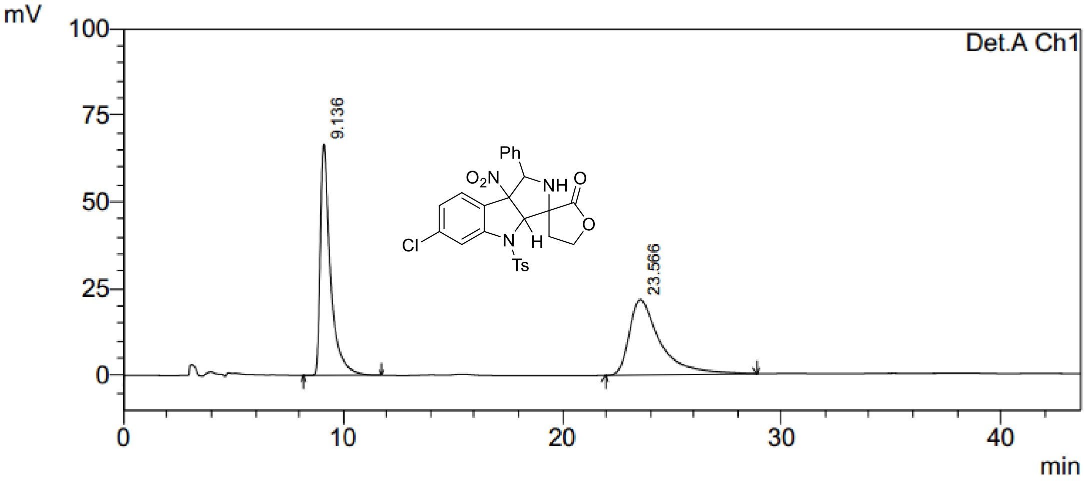

1 Det.A Ch1/254nm

PeakTable

Detector A Ch1 254nm

Peak#	Ret. Time	Area	Height	Area %
1	9.136	2139465	66667	50.197
2	23.566	2122697	21718	49.803
Total		4262162		100.000

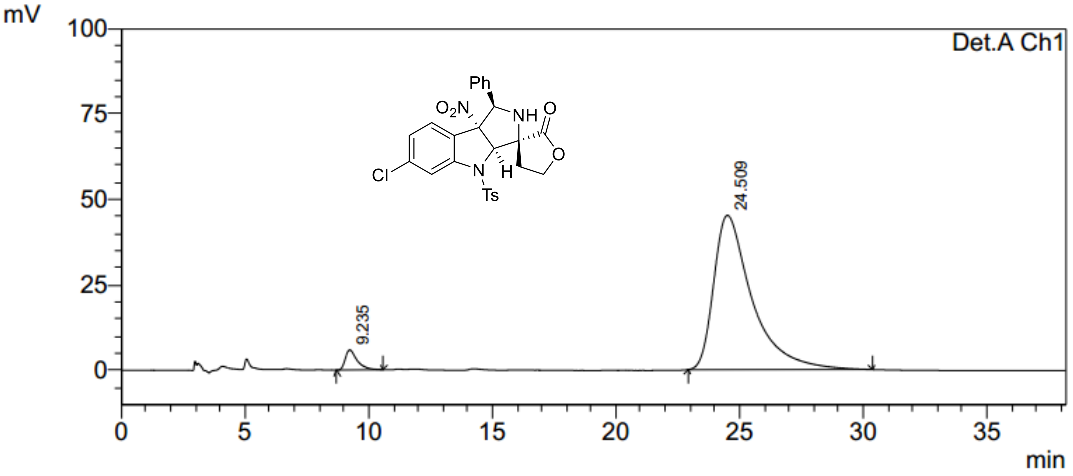

1 Det.A Ch1/254nm

PeakTable

Detector A Ch1 254nm

Peak#	Ret. Time	Area	Height	Area %
1	9.235	197435	5885	3.892
2	24.509	4875199	45129	96.108
Total		5072634		100.000

^{1}H NMR (400 MHz, DMSO-d_6) and ^{13}C NMR (101 MHz, DMSO-d_6) of 3h

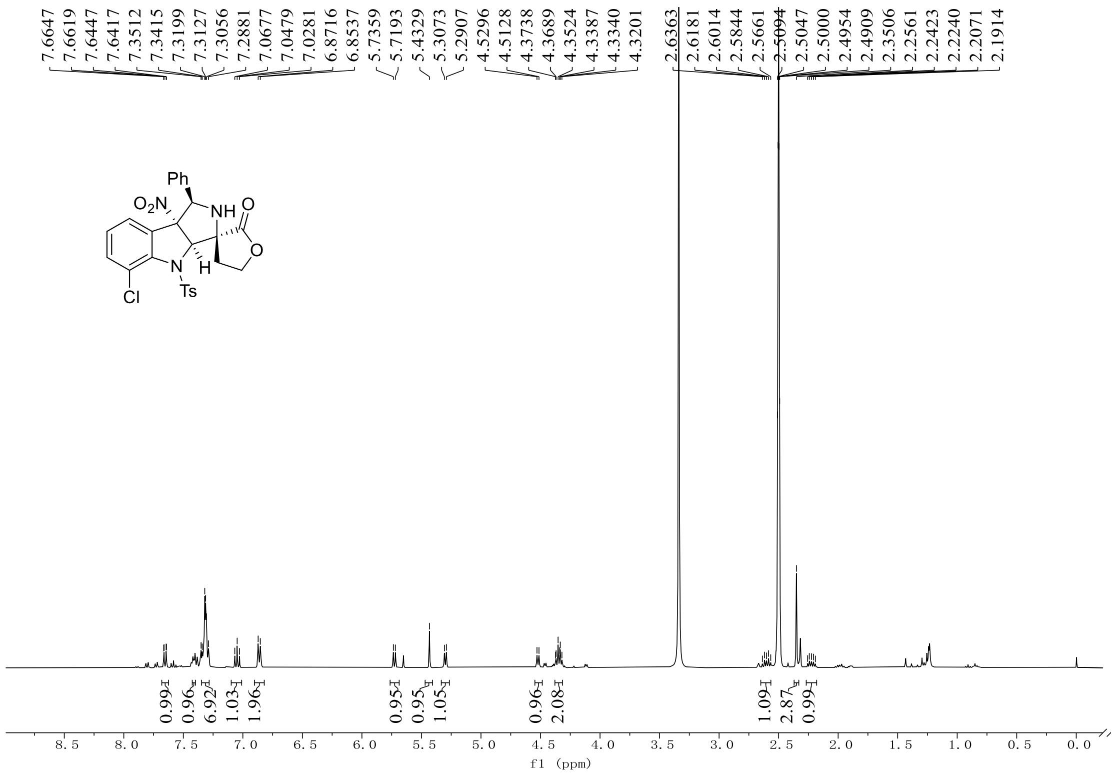

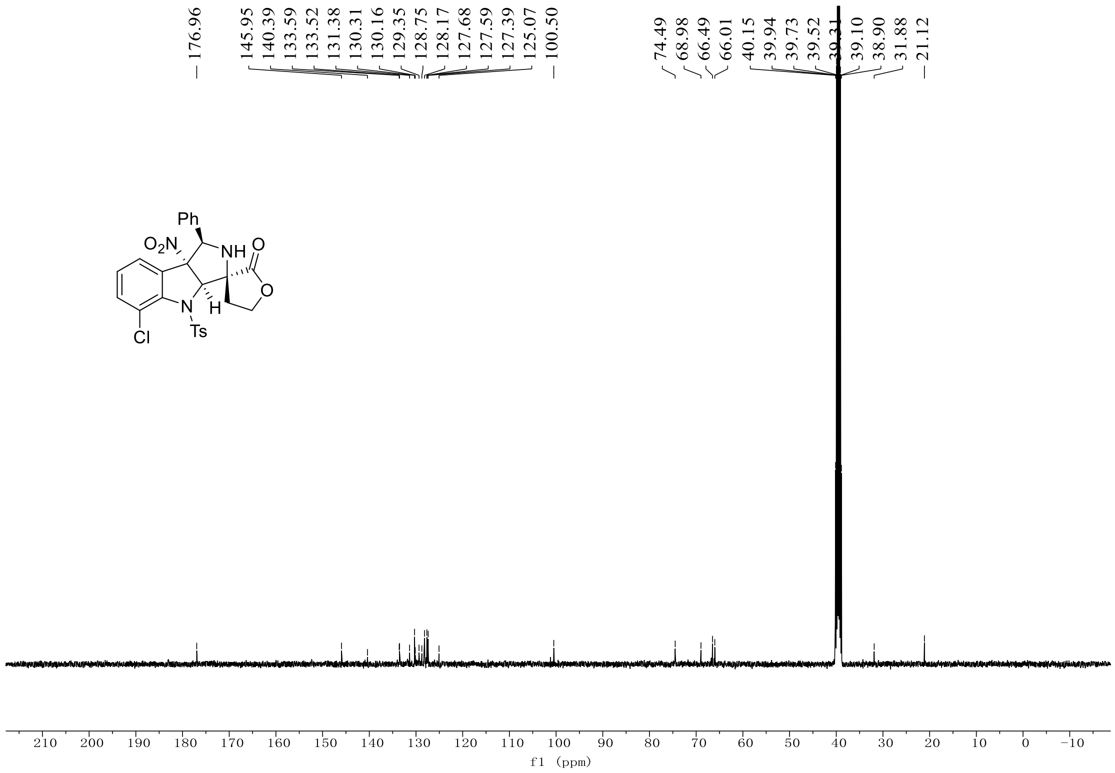

HPLC spectra of 3h

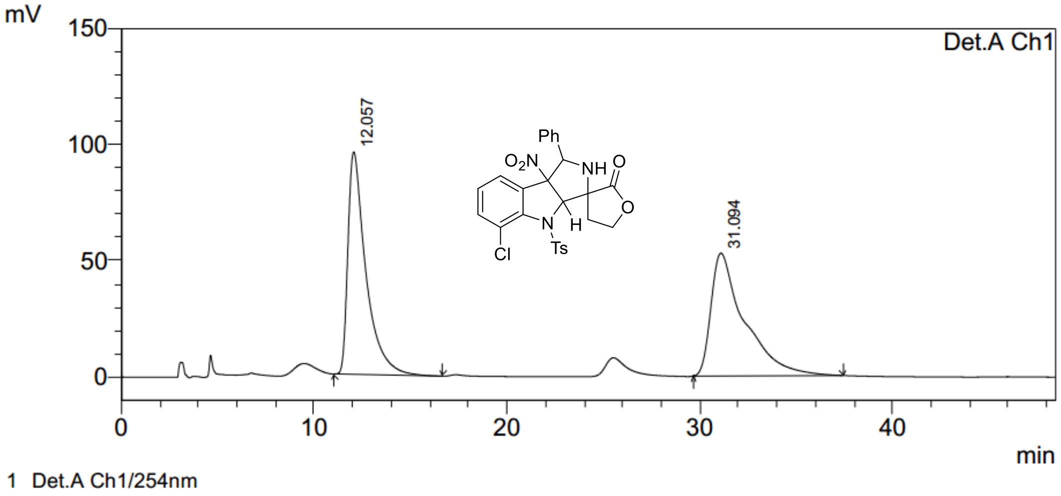

PeakTable

Detector A Ch1 254nm

Peak#	Ret. Time	Area	Height	Area %
1	12.057	6189274	95583	49.261
2	31.094	6374966	52778	50.739
Total		12564239		100.000

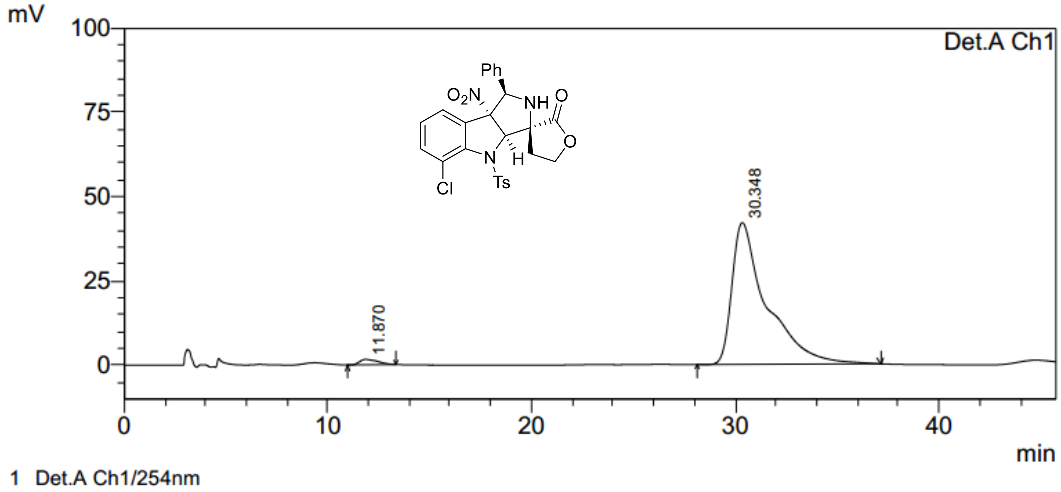

PeakTable

Detector A Ch1 254nm

Peak#	Ret. Time	Area	Height	Area %
1	11.870	99525	1607	1.962
2	30.348	4973741	42034	98.038
Total		5073266		100.000

^{1}H NMR (400 MHz, DMSO-d_6) and ^{13}C NMR (101 MHz, DMSO-d_6) of 3i

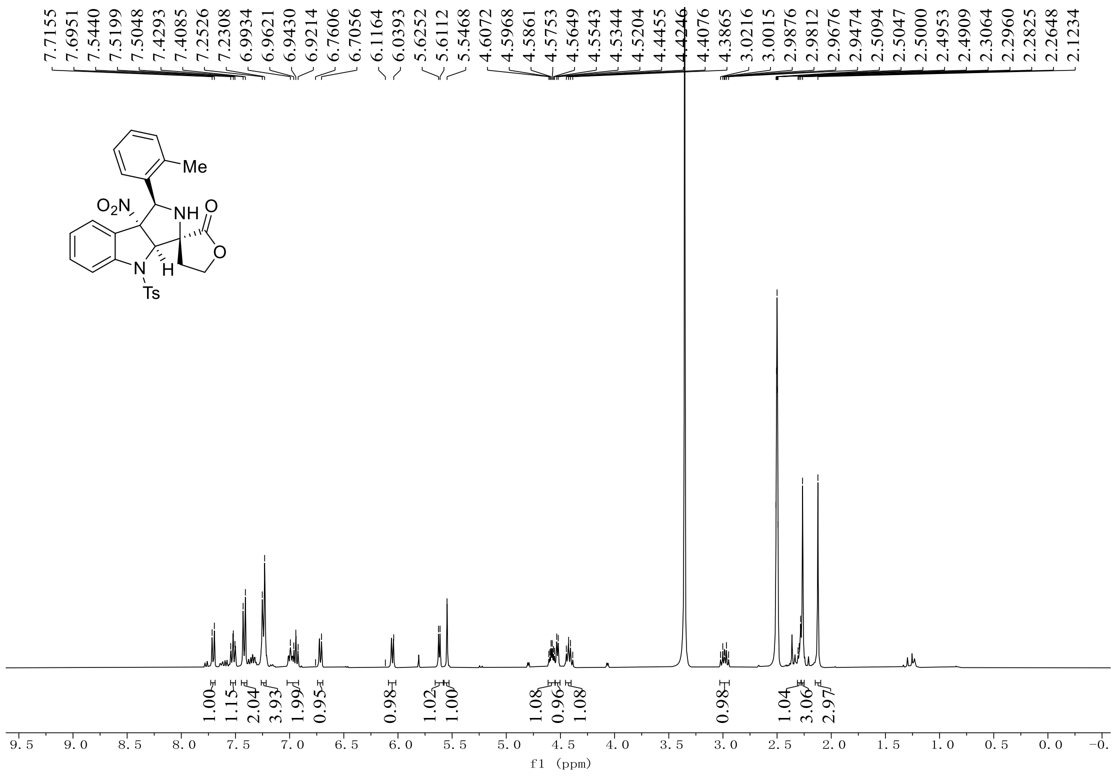

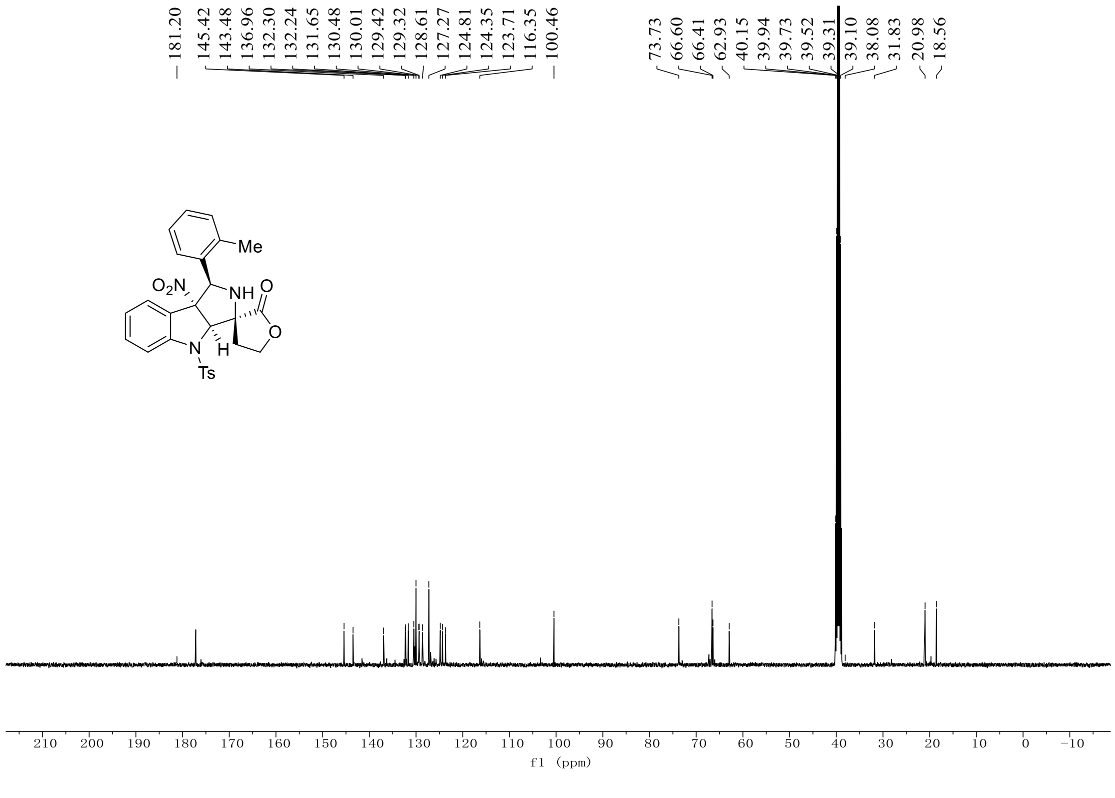

HPLC spectra of 3i

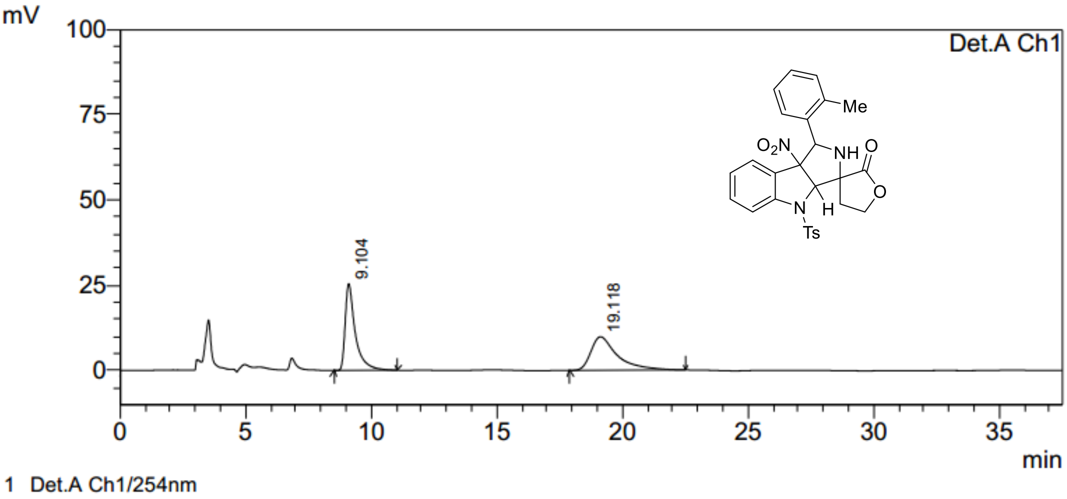

PeakTable

Detector A Ch1 254nm

Peak#	Ret. Time	Area	Height	Area %
1	9.104	711498	25428	49.721
2	19.118	719476	9815	50.279
Total		1430974		100.000

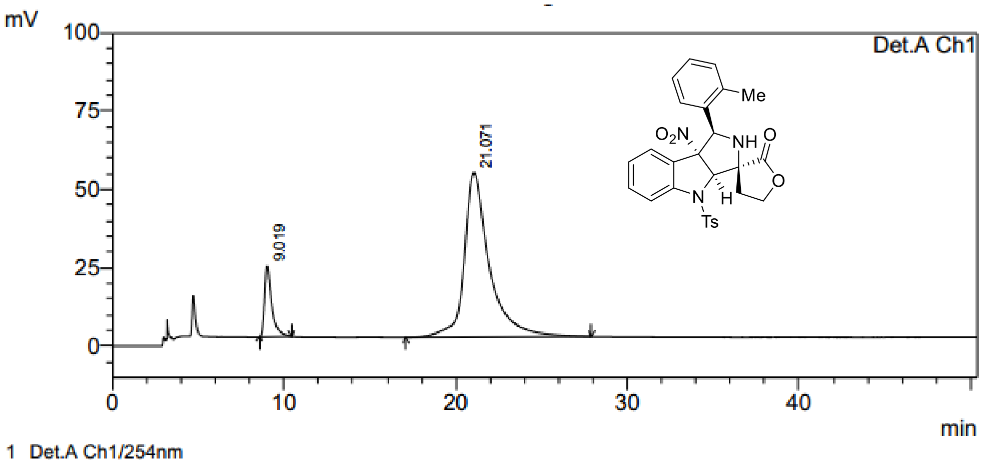

PeakTable

Detector A Ch1 254nm

Peak#	Ret. Time	Area	Height	Area %
1	9.019	668440	22664	11.091
2	21.071	5358264	52426	88.909
Total		6026704		100.000

^{1}H NMR (400 MHz, DMSO-d_6) and ^{13}C NMR (101 MHz, DMSO-d_6) of 3j

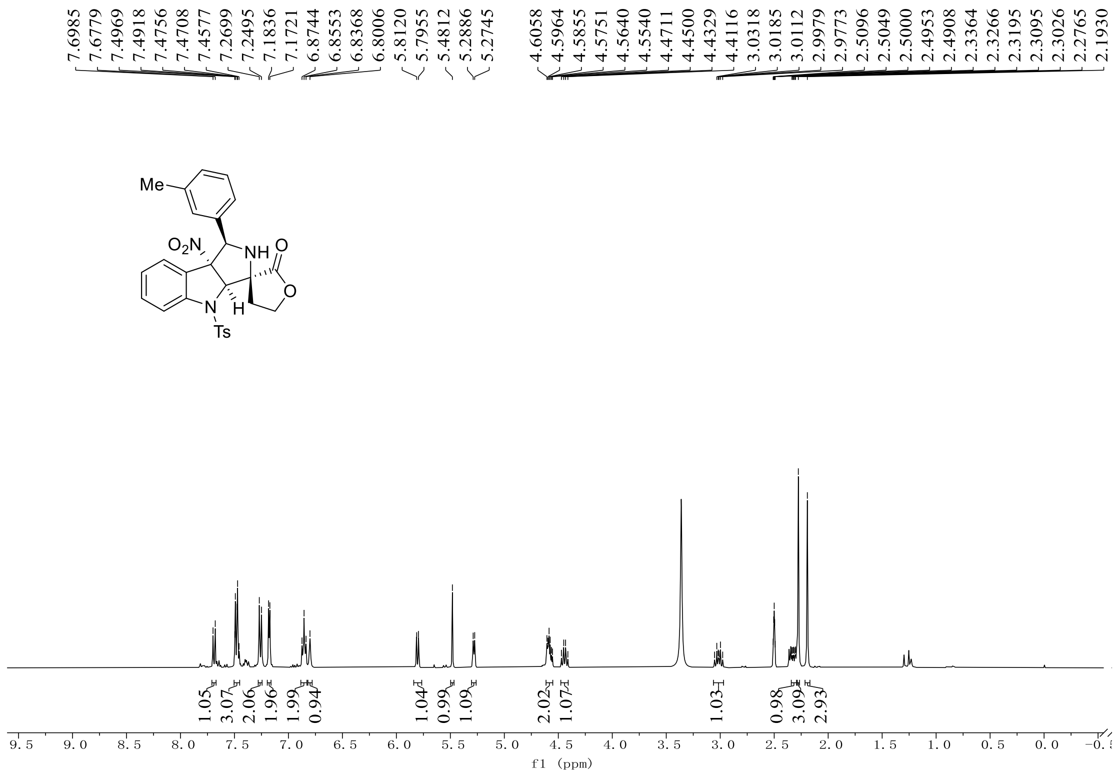

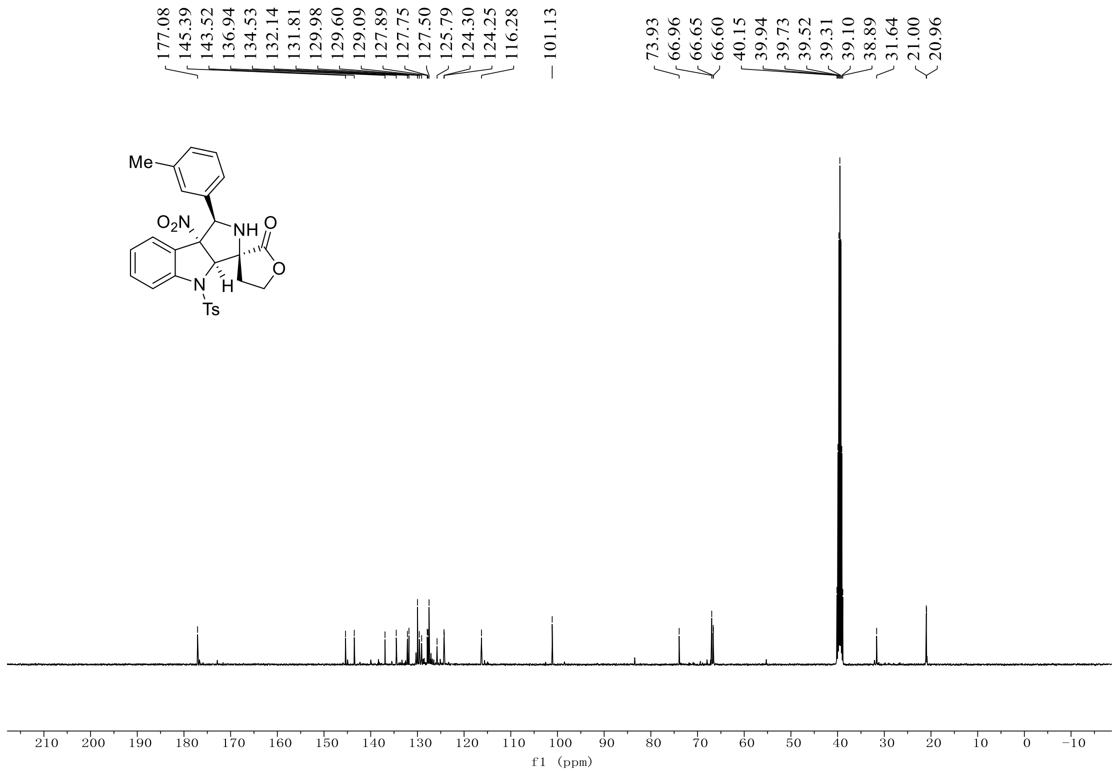

HPLC spectra of 3j

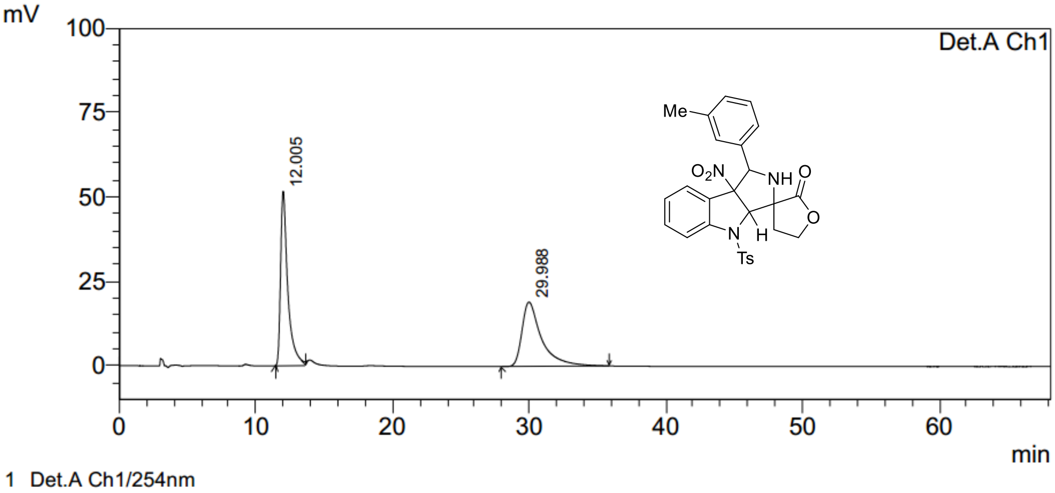

PeakTable

Detector A Ch1 254nm

Peak#	Ret. Time	Area	Height	Area %
1	12.005	1897125	51641	50.041
2	29.988	1894024	19032	49.959
Total		3791149		100.000

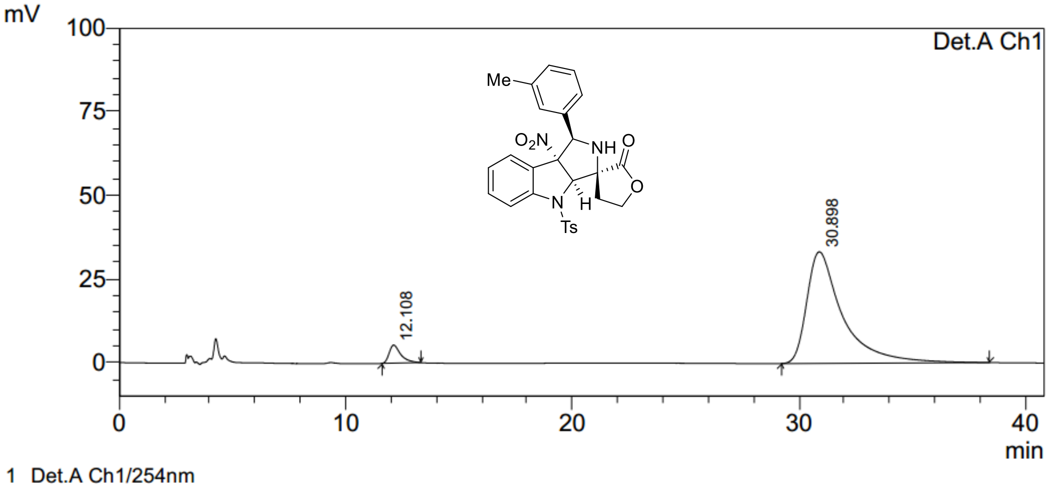

PeakTable

Detector A Ch1 254nm

Peak#	Ret. Time	Area	Height	Area %
1	12.108	194276	5420	5.024
2	30.898	3672663	33306	94.976
Total		3866938		100.000

^{1}H NMR (400 MHz, DMSO-d_6) and ^{13}C NMR (101 MHz, DMSO-d_6) of 3k

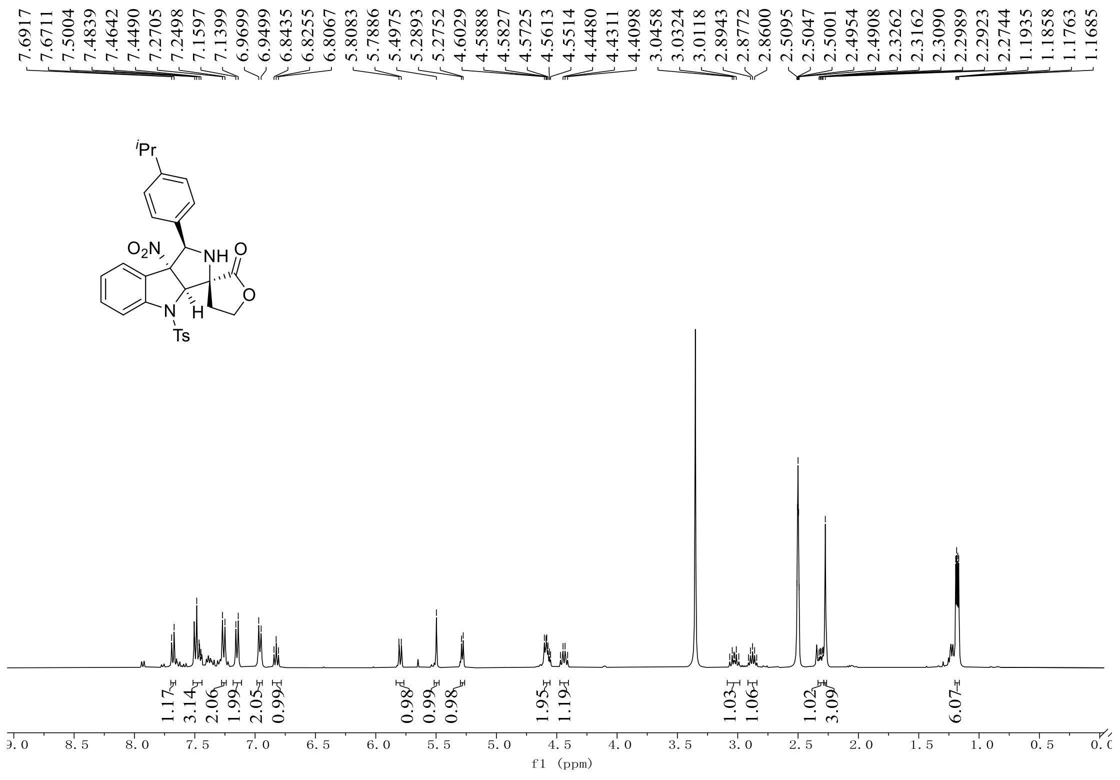

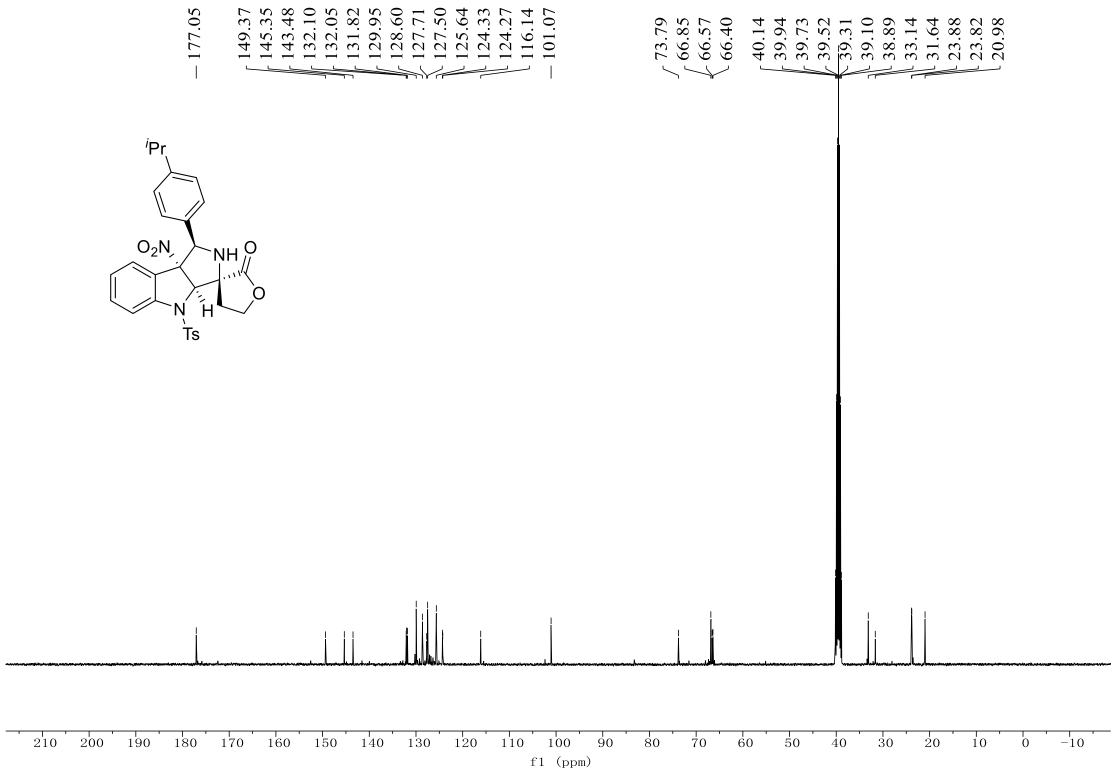

HPLC spectra of 3k

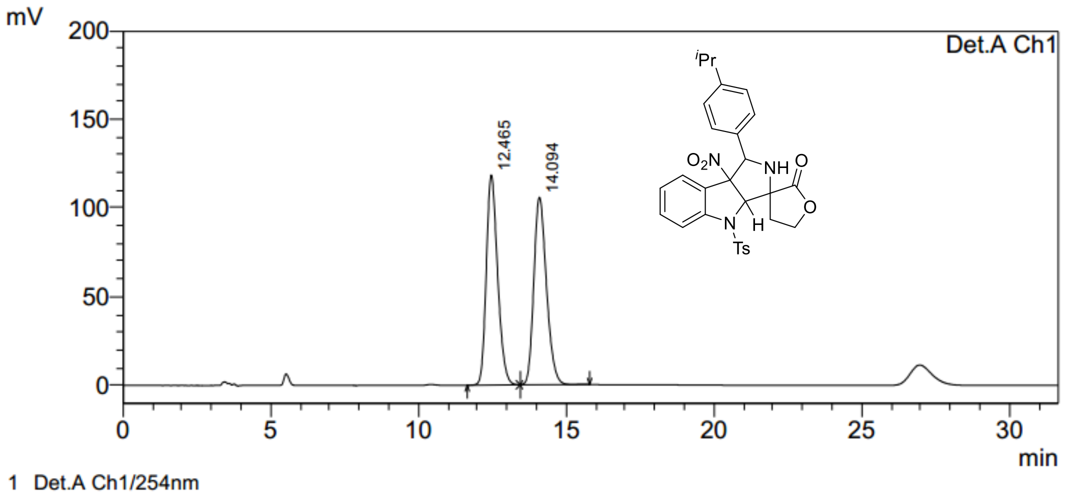

PeakTable

Detector A Ch1 254nm

Peak#	Ret. Time	Area	Height	Area %
1	12.465	3155874	118369	50.061
2	14.094	3148129	105744	49.939
Total		6304002		100.000

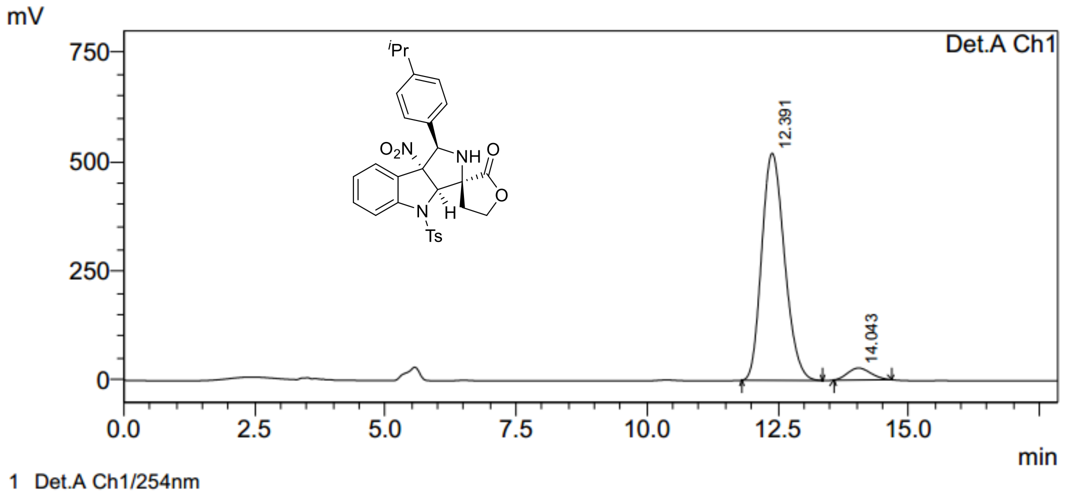

PeakTable

Detector A Ch1 254nm

Peak#	Ret. Time	Area	Height	Area %
1	12.391	15719711	518659	94.942
2	14.043	837408	27133	5.058
Total		16557120		100.000

^{1}H NMR (400 MHz, DMSO-d_6) and ^{13}C NMR (101 MHz, DMSO-d_6) of 3l

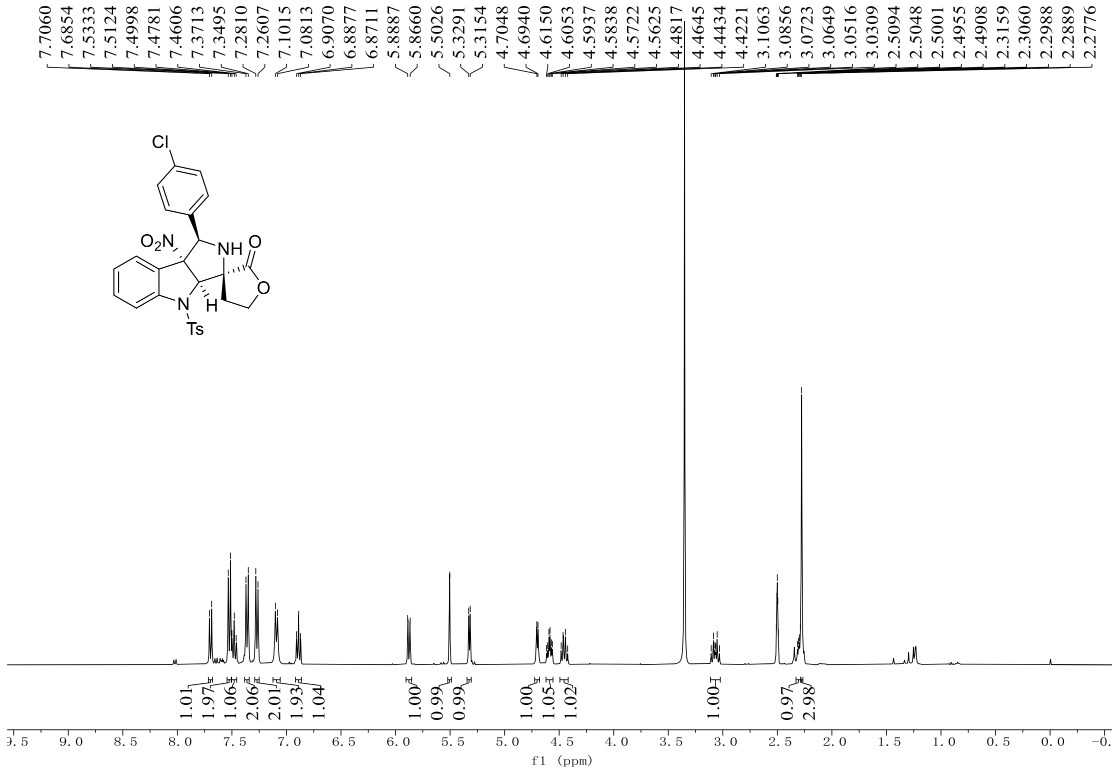

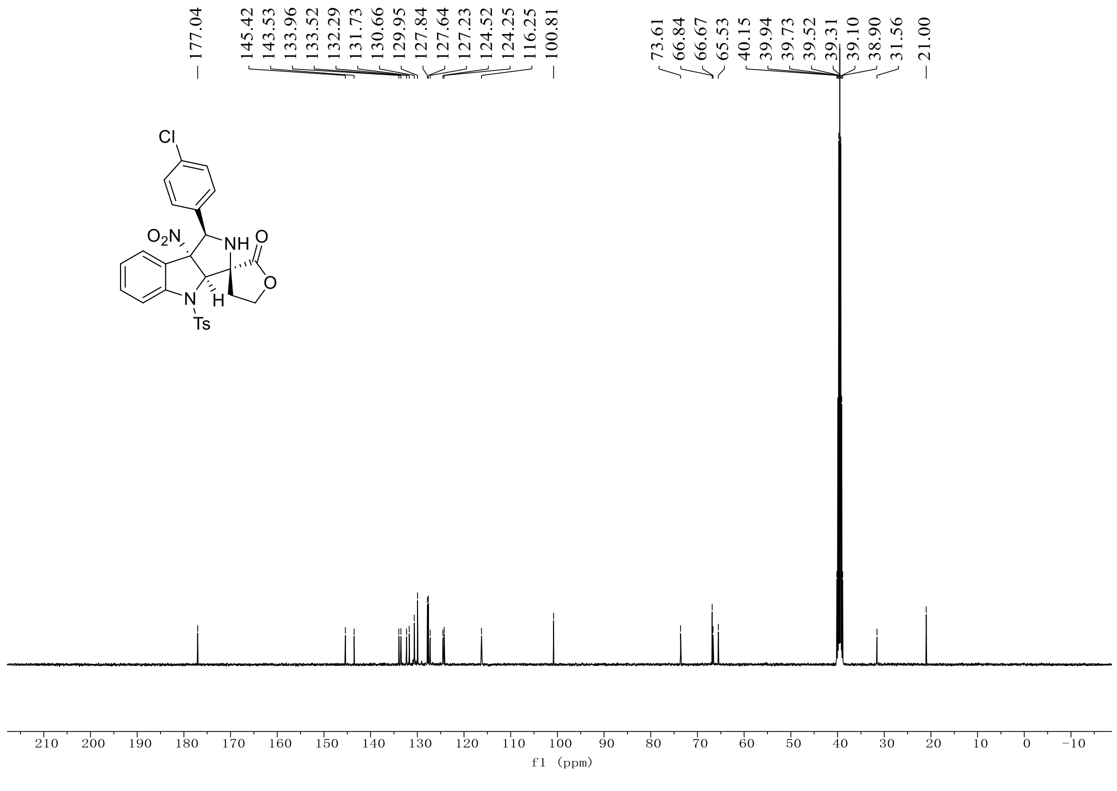

HPLC spectra of 3l

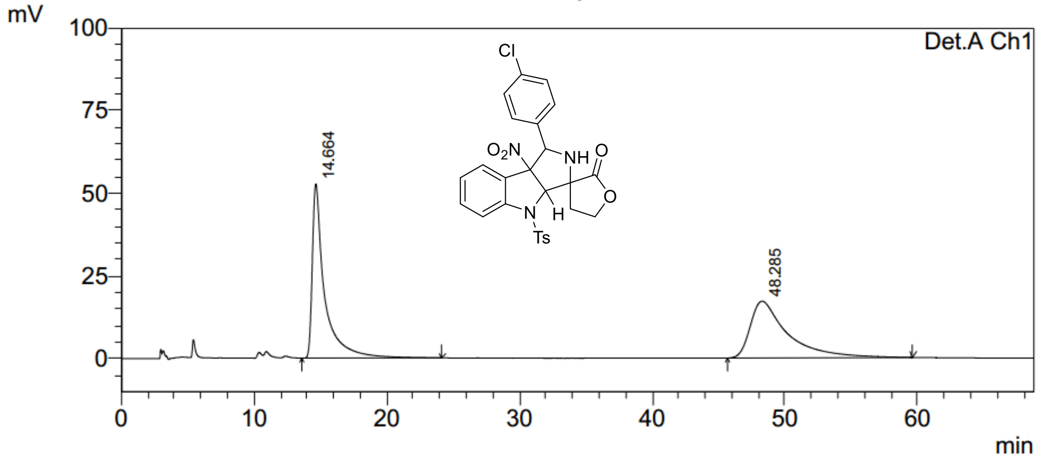

1 Det.A Ch1/254nm

PeakTable

Detector A Ch1 254nm

Peak#	Ret. Time	Area	Height	Area %
1	14.664	3396511	52588	50.343
2	48.285	3350258	17127	49.657
Total		6746769		100.000

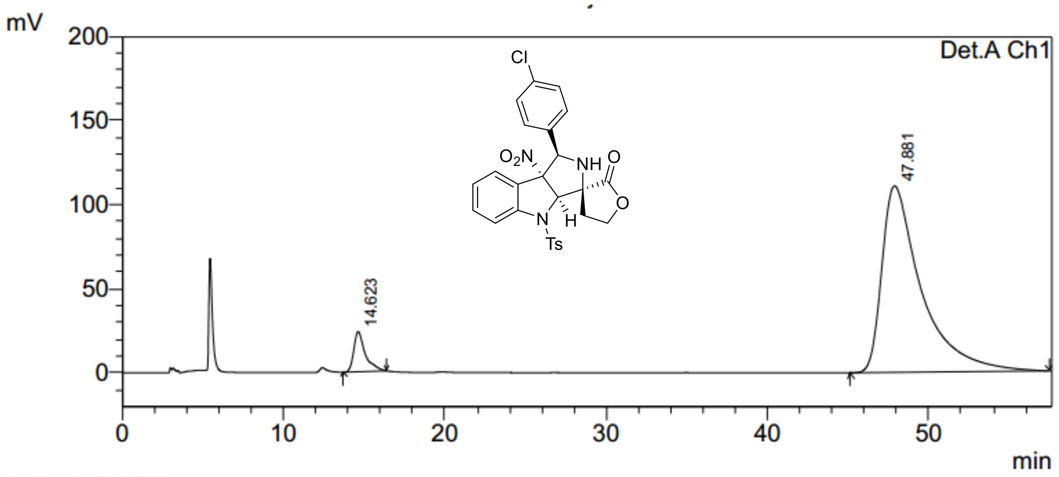

1 Det.A Ch1/254nm

PeakTable

Detector A Ch1 254nm

Peak#	Ret. Time	Area	Height	Area %
1	14.623	1182802	23923	5.908
2	47.881	18837911	110717	94.092
Total		20020712		100.000

^{1}H NMR (400 MHz, DMSO-d_6) and ^{13}C NMR (101 MHz, DMSO-d_6) of 3m

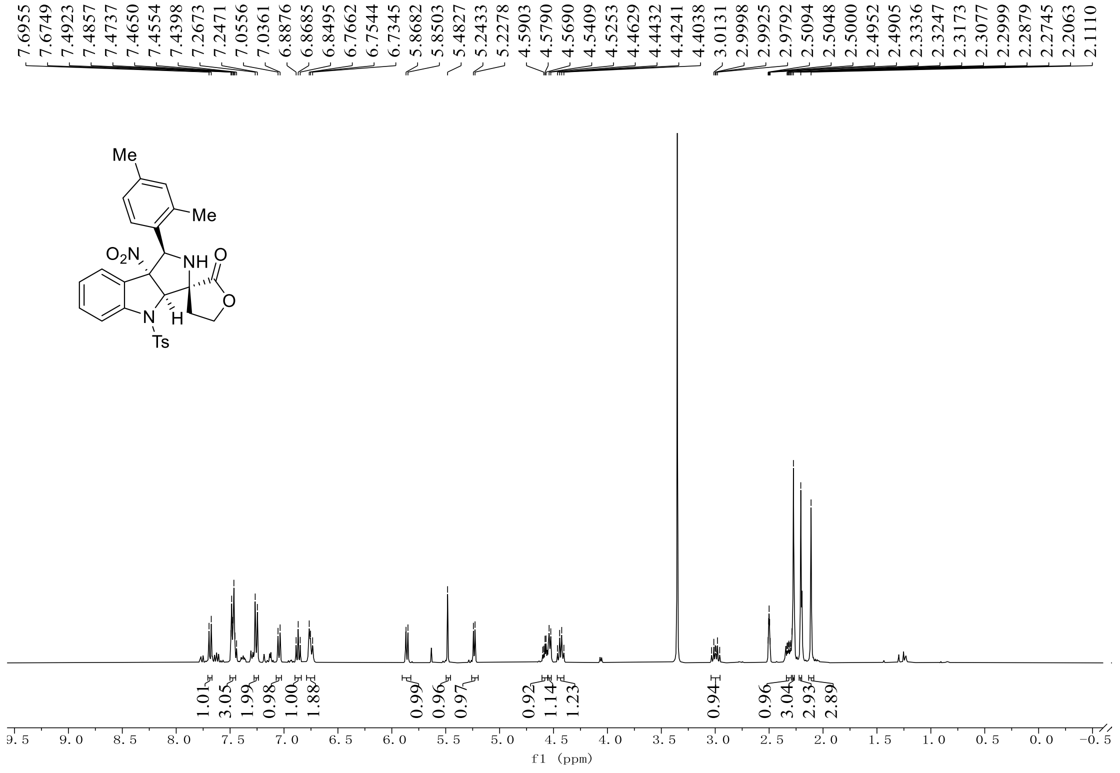

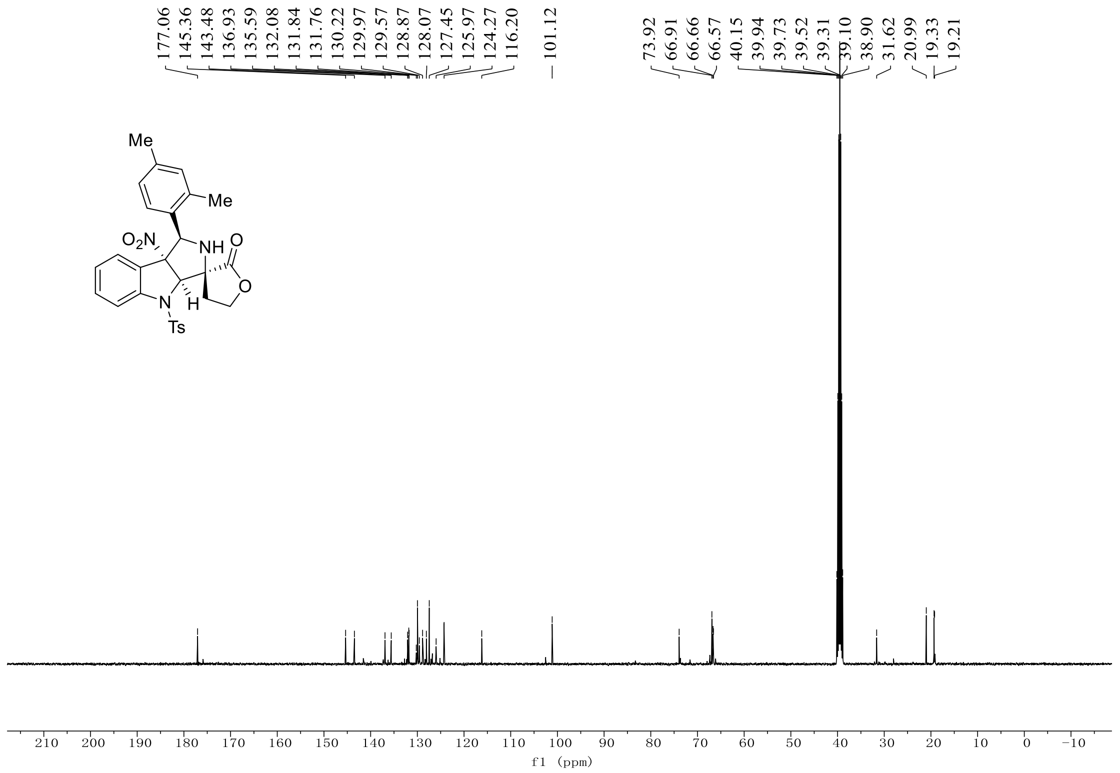

HPLC spectra of 3m

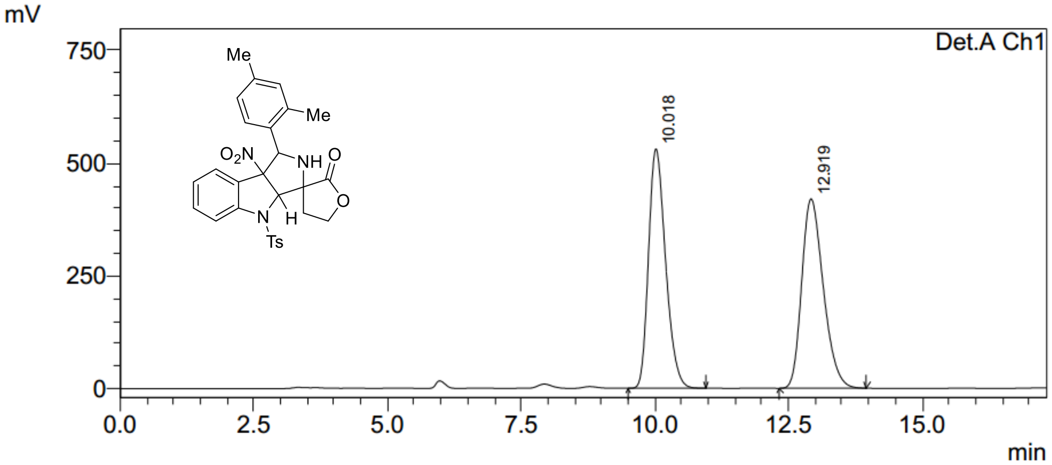

1 Det.A Ch1/254nm

PeakTable

Detector A Ch1 254nm

Peak#	Ret. Time	Area	Height	Area %
1	10.018	11783053	530770	49.944
2	12.919	11809406	419987	50.056
Total		23592459		100.000

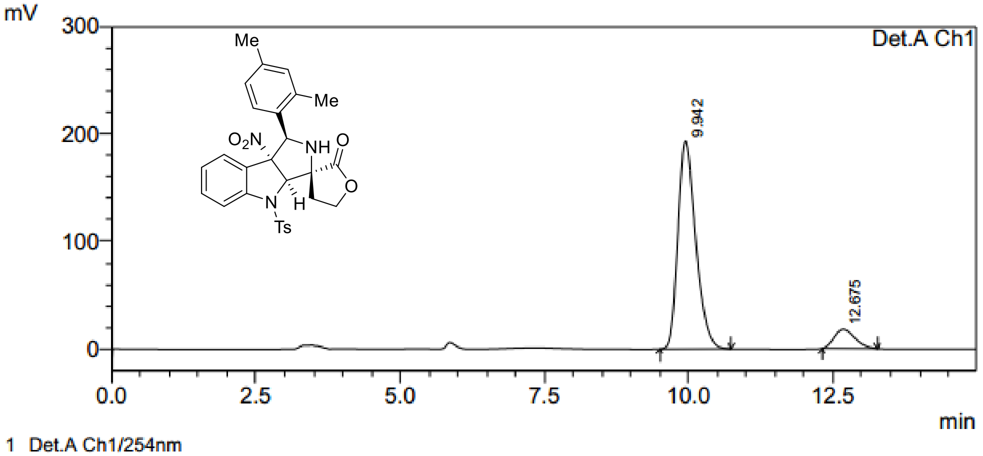

1 Det.A Ch1/254nm

PeakTable

Detector A Ch1 254nm

Peak#	Ret. Time	Area	Height	Area %
1	9.942	4138827	193173	90.233
2	12.675	448019	17857	9.767
Total		4586845		100.000

^{1}H NMR (400 MHz, DMSO-d_6) and ^{13}C NMR (101 MHz, DMSO-d_6) of 3n

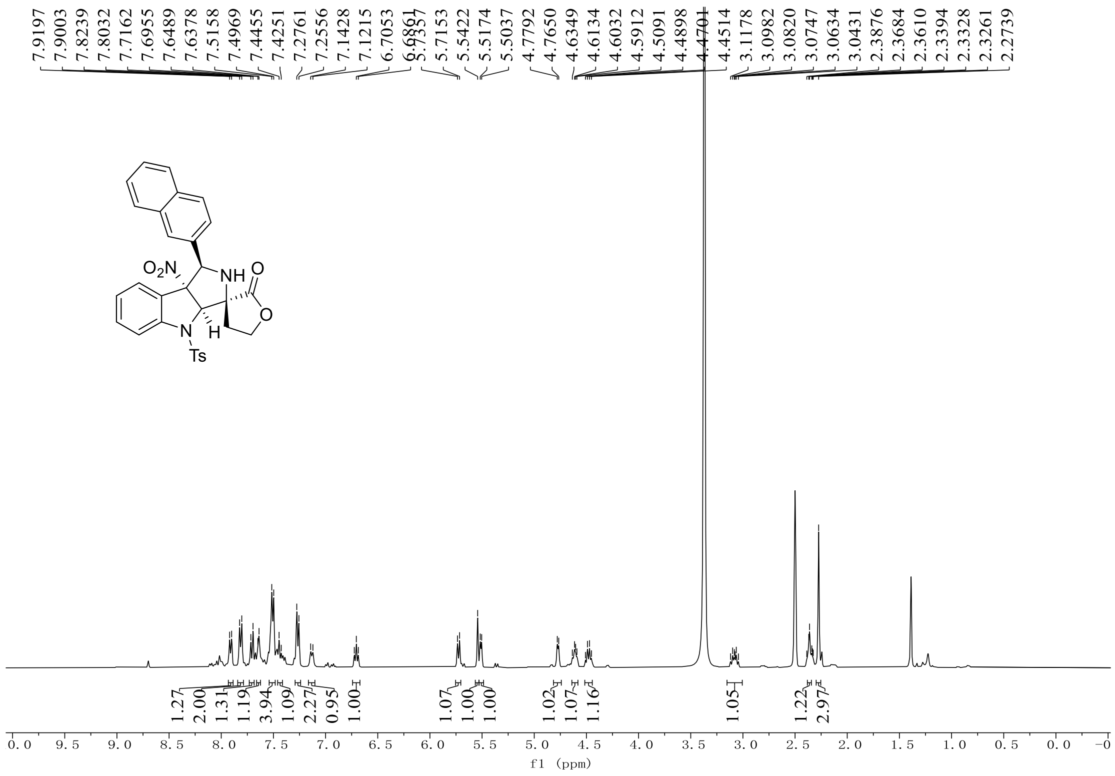

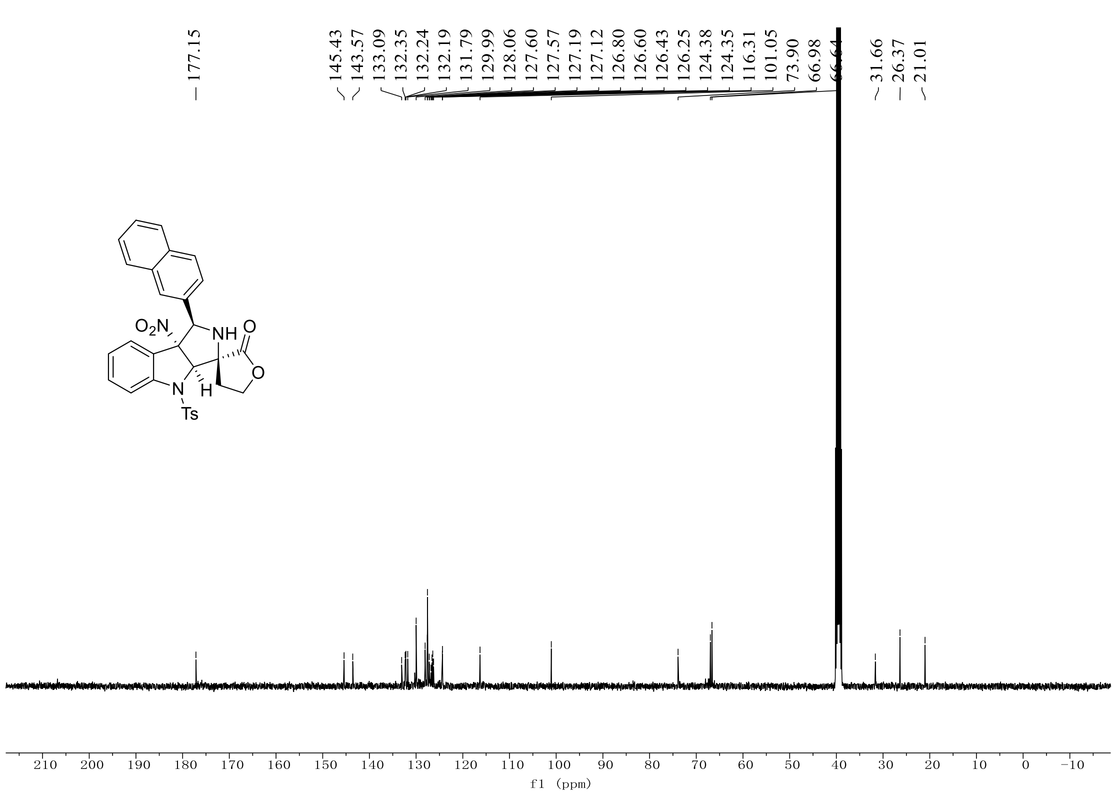

HPLC spectra of 3n

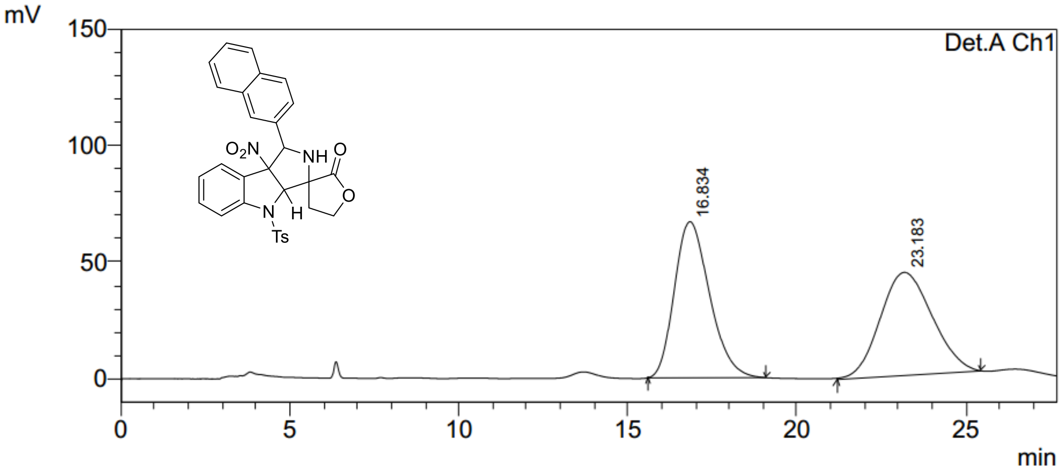

1 Det.A Ch1/254nm

PeakTable

Detector A Ch1 254nm

Peak#	Ret. Time	Area	Height	Area %
1	16.834	4847732	66825	50.576
2	23.183	4737280	44168	49.424
Total		9585012		100.000

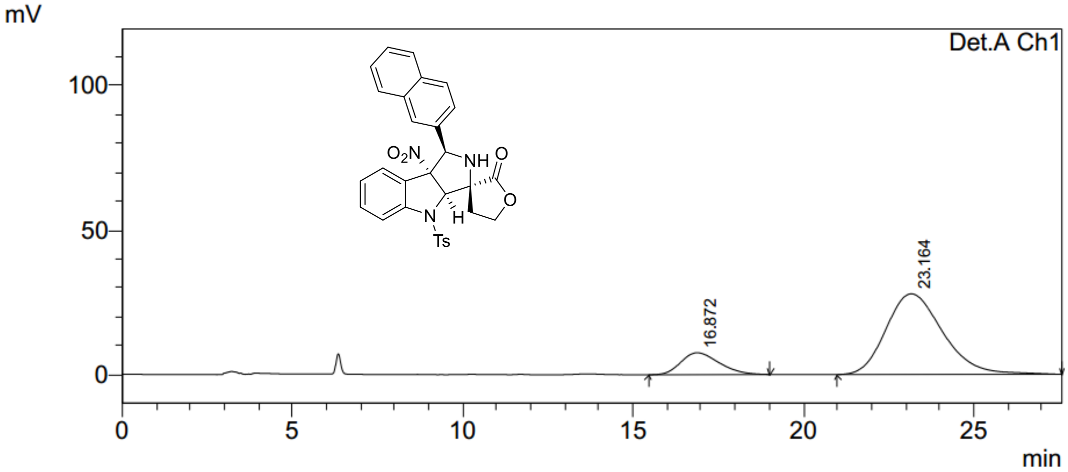

1 Det.A Ch1/254nm

PeakTable

Detector A Ch1 254nm

Peak#	Ret. Time	Area	Height	Area %
1	16.872	592993	7578	15.449
2	23.164	3245321	27962	84.551
Total		3838315		100.000

^{1}H NMR (400 MHz, DMSO-d_6) and ^{13}C NMR (101 MHz, DMSO-d_6) of 3o

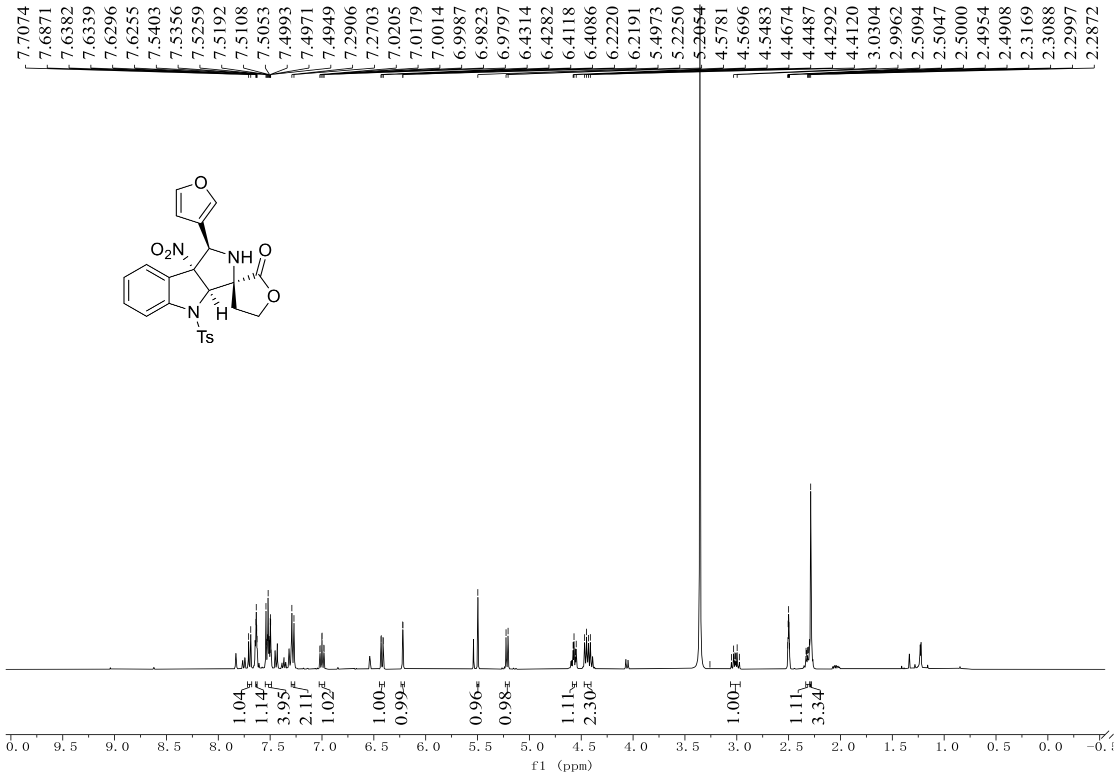

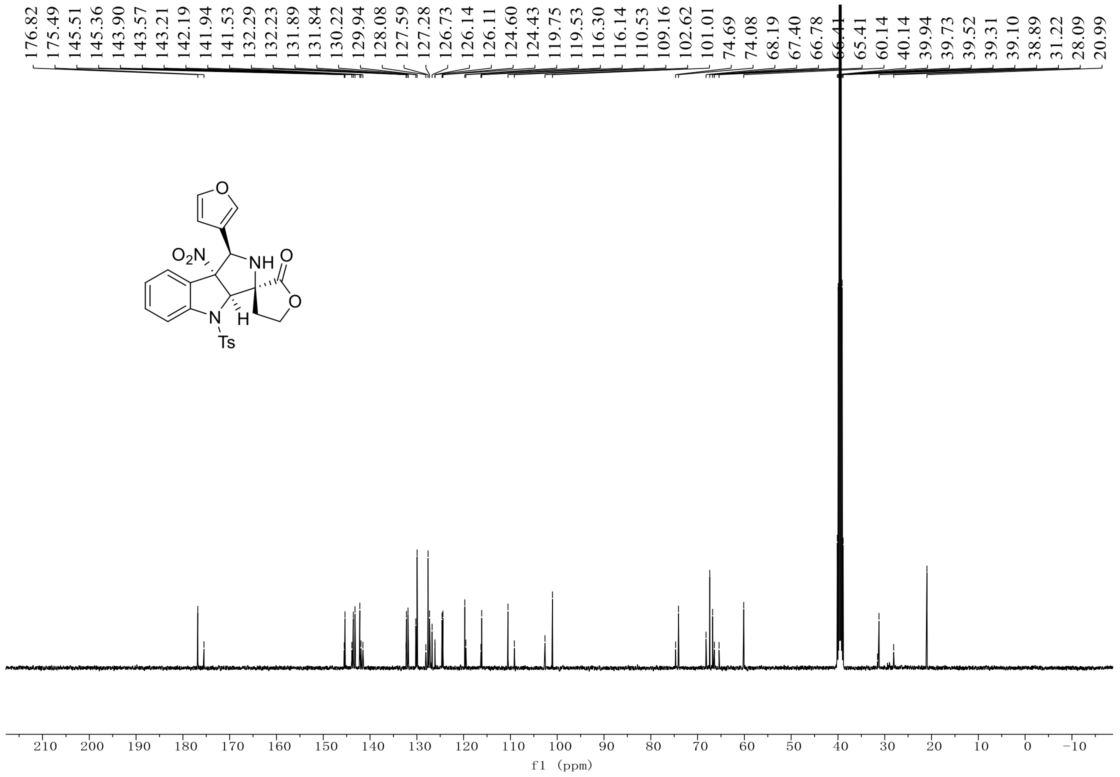

HPLC spectra of 3o

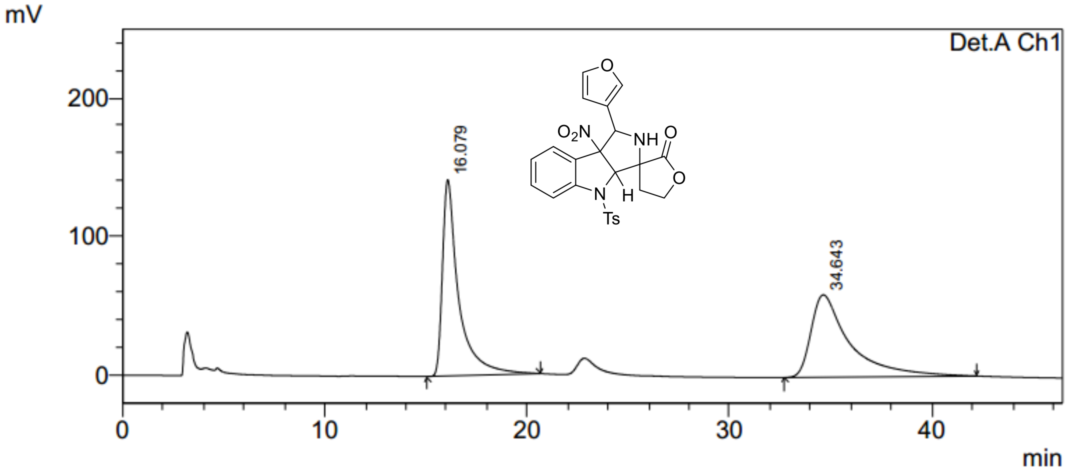

1 Det.A Ch1/220nm

PeakTable

Detector A Ch1 220nm

Peak#	Ret. Time	Area	Height	Area %
1	16.079	7884641	141286	49.830
2	34.643	7938419	59360	50.170
Total		15823060		100.000

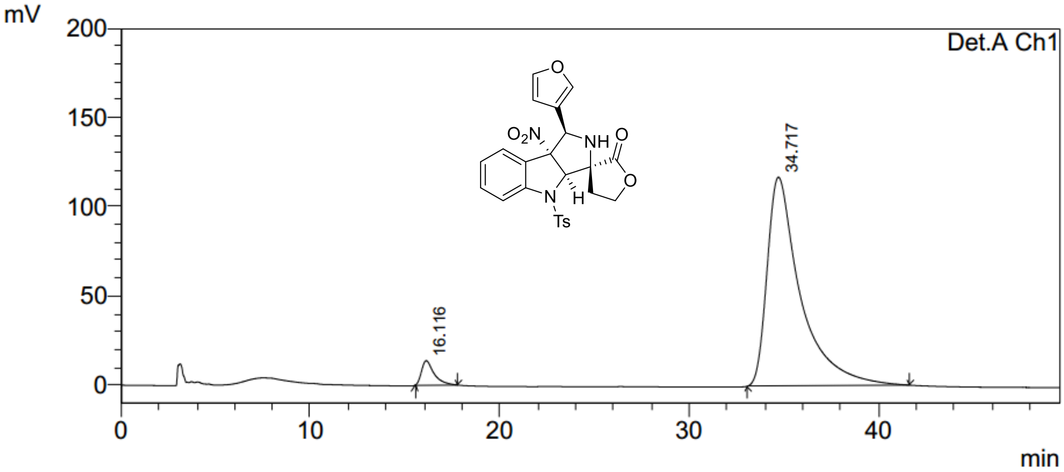

1 Det.A Ch1/220nm

PeakTable

Detector A Ch1 220nm

Peak#	Ret. Time	Area	Height	Area %
1	16.116	644937	13822	4.320
2	34.717	14284340	116887	95.680
Total		14929277		100.000

^{1}H NMR (400 MHz, DMSO-d_6) and ^{13}C NMR (101 MHz, DMSO-d_6) of 5a

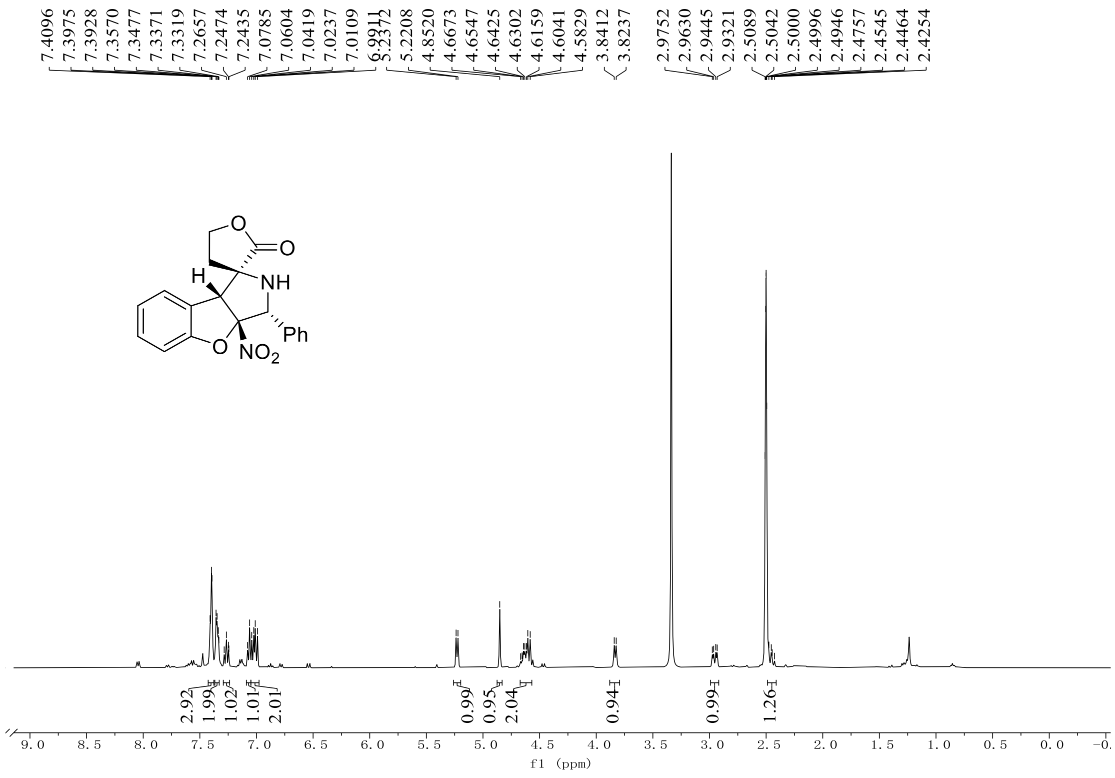

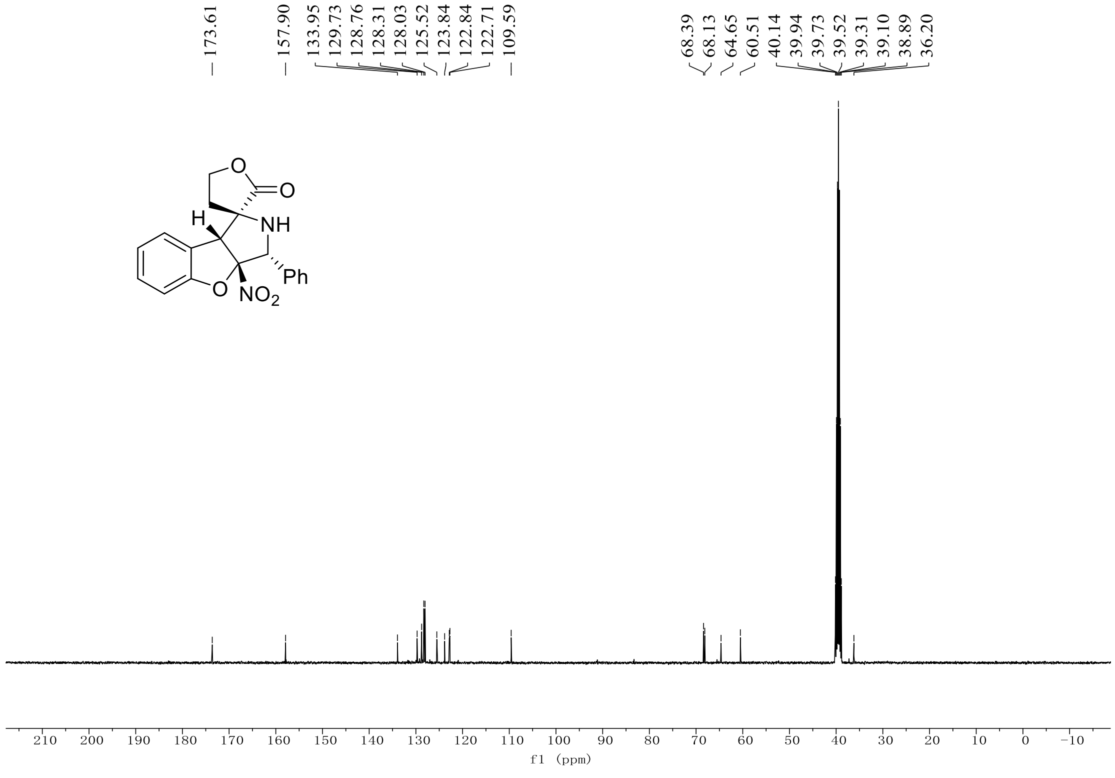

HPLC spectra of 5a

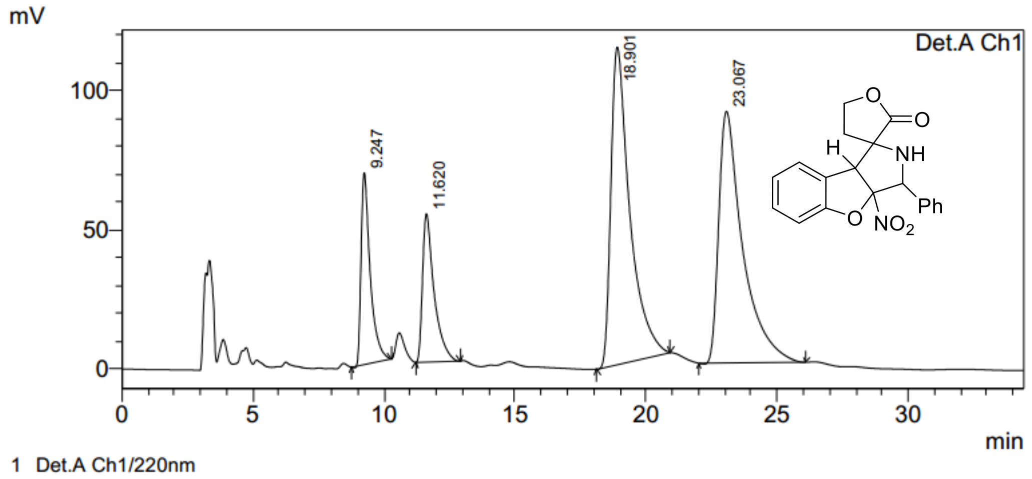

PeakTable

Detector A Ch1 220nm

Peak#	Ret. Time	Area	Height	Area %
1	9.247	1666777	69011	11.276
2	11.620	1636389	53384	11.070
3	18.901	5738022	114193	38.818
4	23.067	5740620	90490	38.836
Total		14781808		100.000

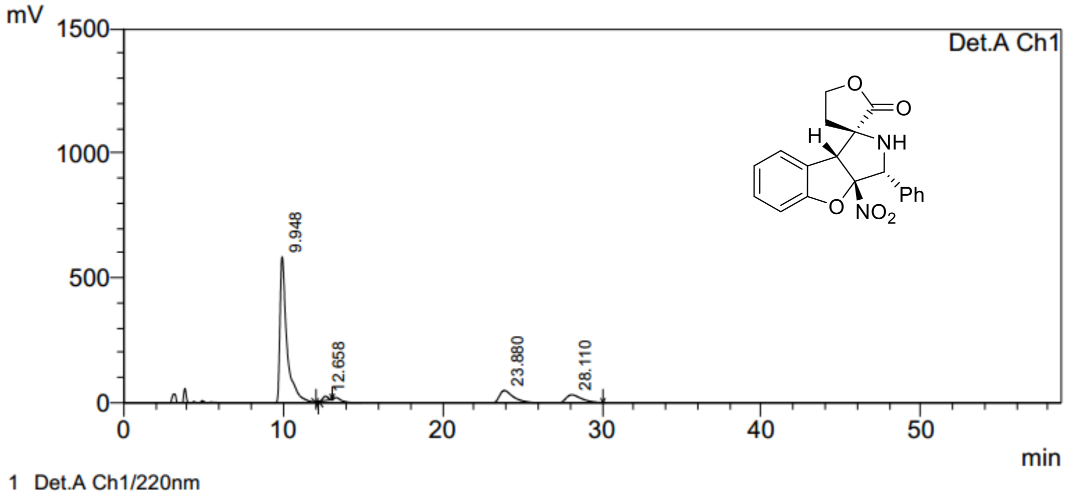

PeakTable

Detector A Ch1 220nm

Peak#	Ret. Time	Area	Height	Area %
1	9.948	17986169	585911	74.694
2	12.658	374132	17478	1.554
3	23.880	3349278	52267	13.909
4	28.110	2370149	33695	9.843
Total		24079728		100.000

^{1}H NMR (400 MHz, Chloroform-*d*) and ^{13}C NMR (101 MHz, DMSO-d_6) of 5b

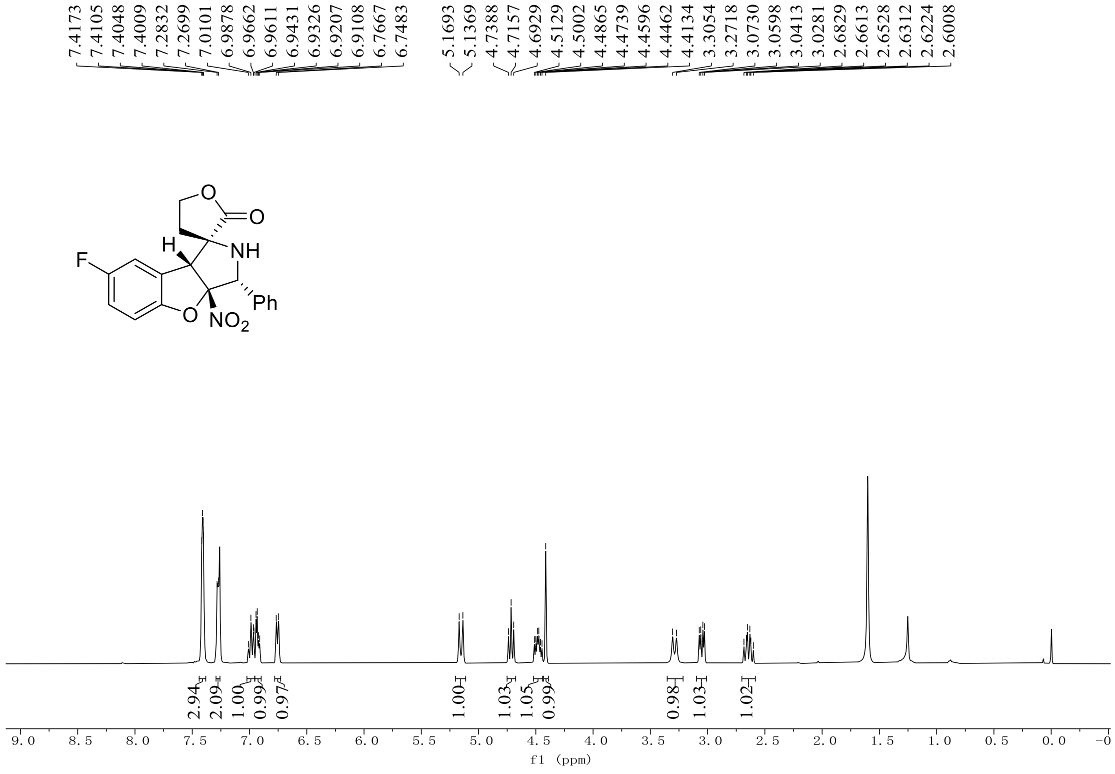

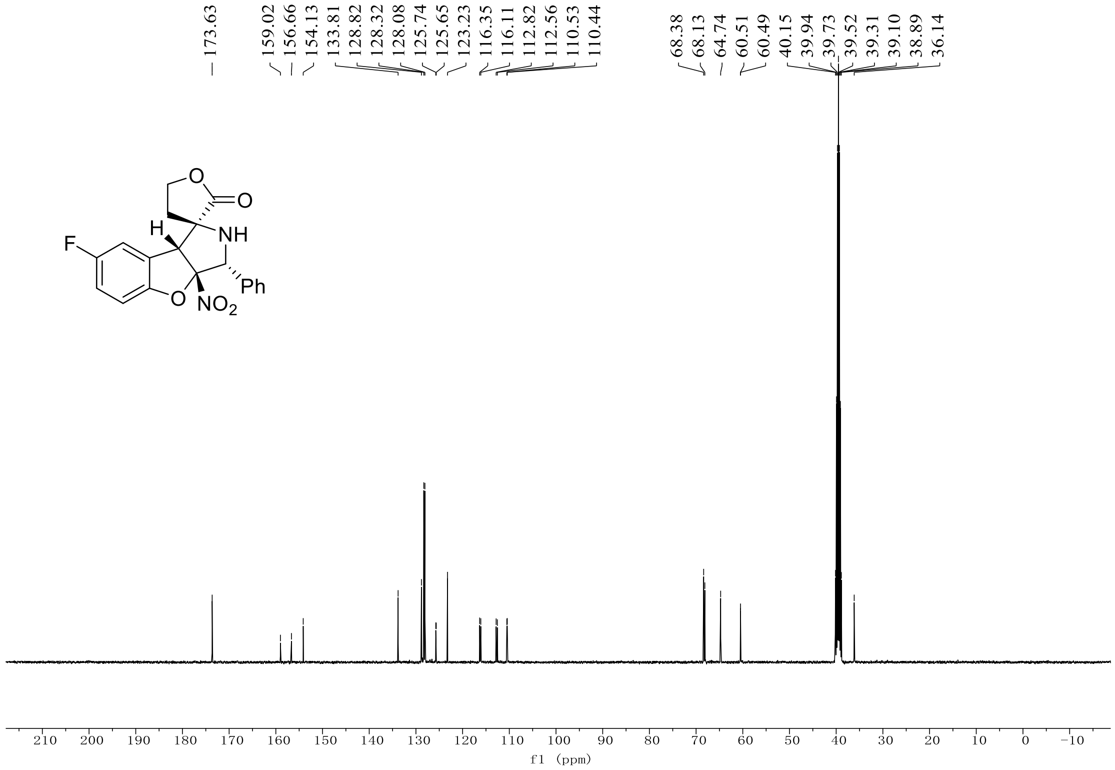

HPLC spectra of 5b

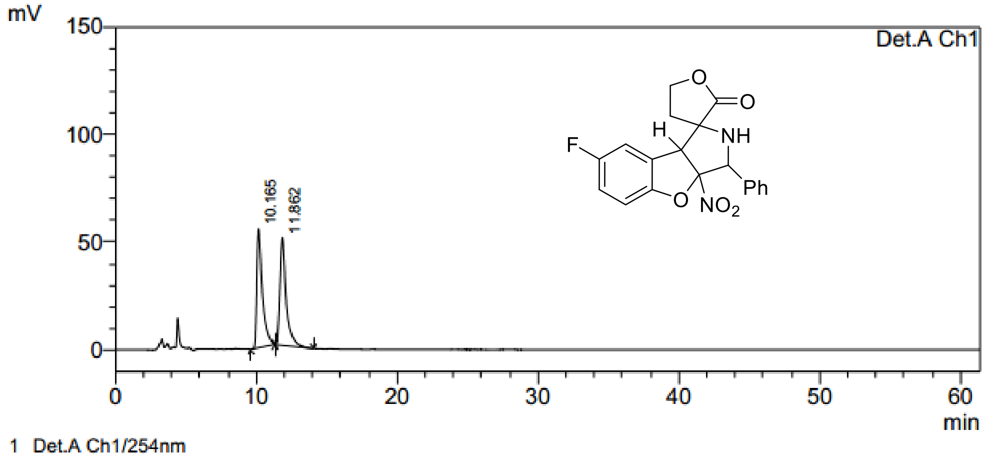

PeakTable

Detector A Ch1 254nm

Peak#	Ret. Time	Area	Height	Area %
1	10.165	1525355	55085	49.631
2	11.862	1548020	49958	50.369
Total		3073375		100.000

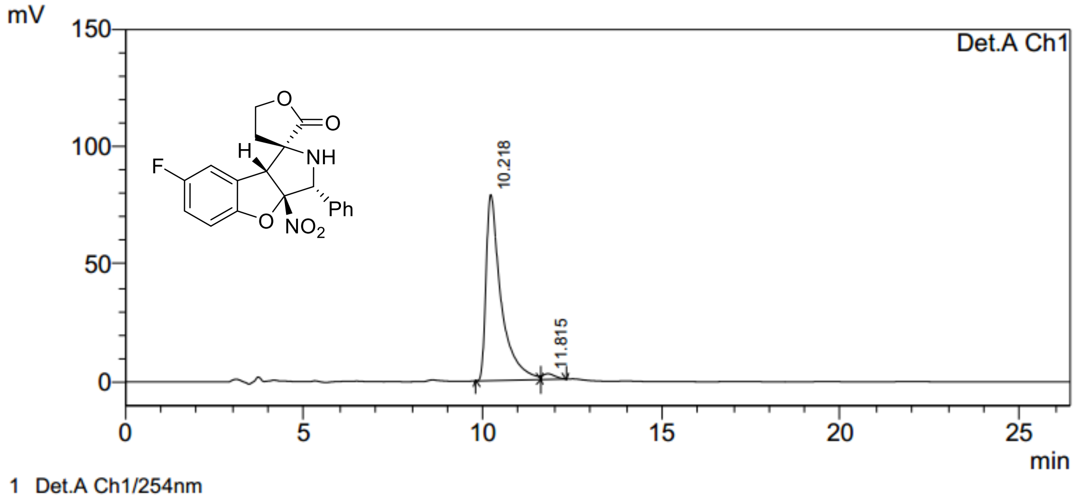

PeakTable

Detector A Ch1 254nm

Peak#	Ret. Time	Area	Height	Area %
1	10.218	2305886	79013	97.469
2	11.815	59875	2411	2.531
Total		2365761		100.000

^{1}H NMR (400 MHz, DMSO-d_6) and ^{13}C NMR (101 MHz, DMSO-d_6) of 5c

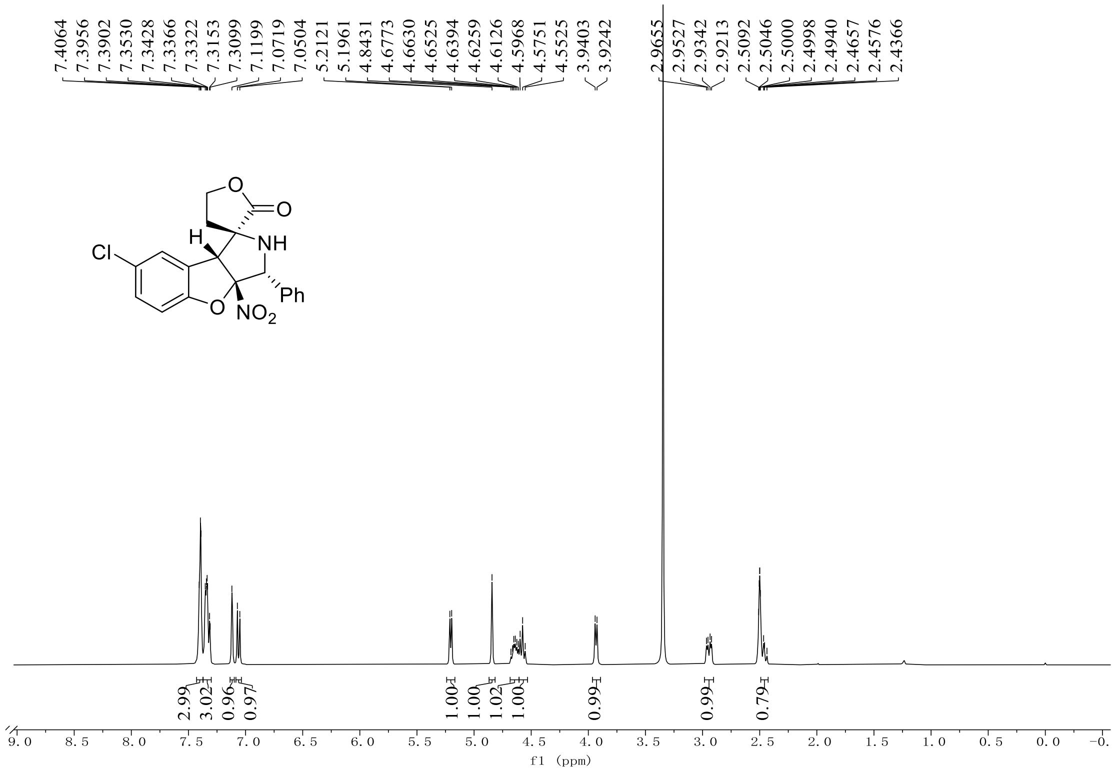

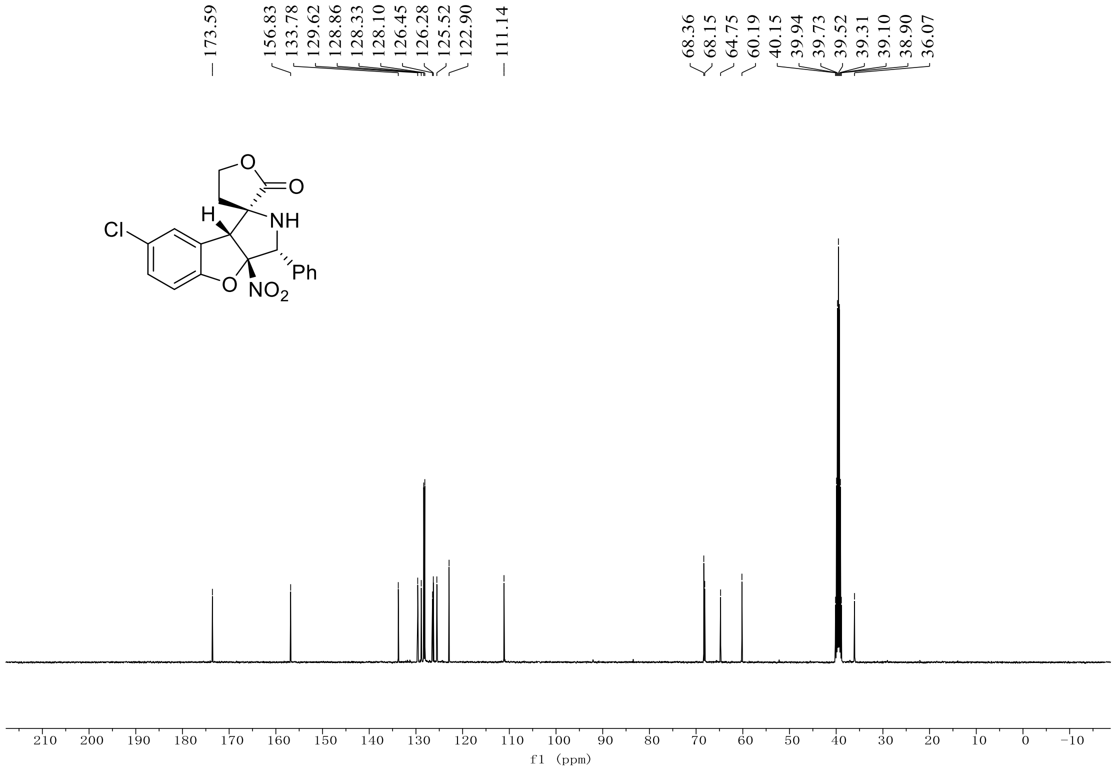

HPLC spectra of 5c

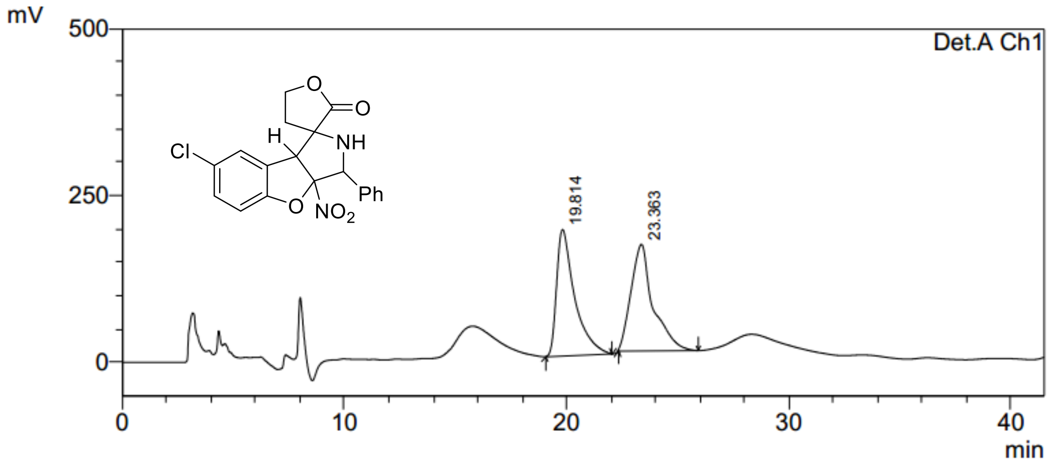

1 Det.A Ch1/220nm

PeakTable

Detector A Ch1 220nm

Peak#	Ret. Time	Area	Height	Area %
1	19.814	10708569	189445	49.268
2	23.363	11026718	159489	50.732
Total		21735287		100.000

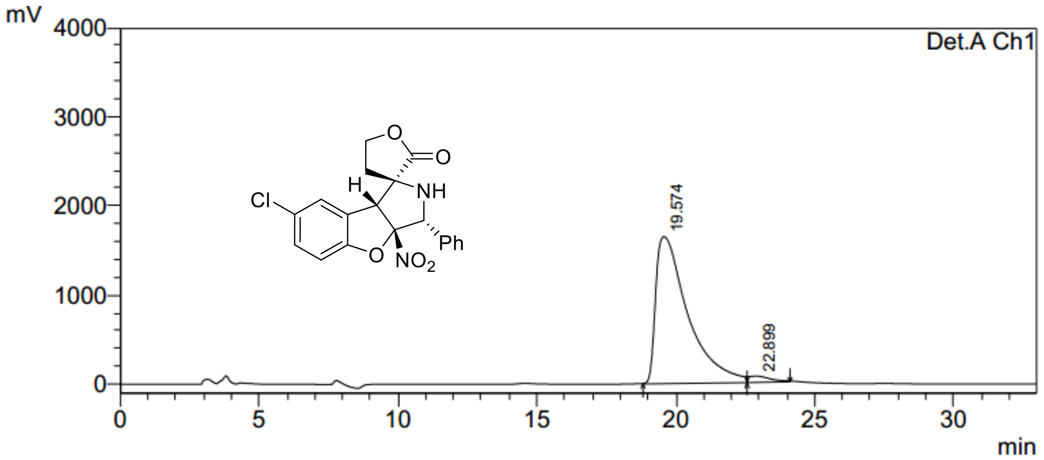

1 Det.A Ch1/220nm

PeakTable

Detector A Ch1 220nm

Peak#	Ret. Time	Area	Height	Area %
1	19.574	133099290	1650021	97.102
2	22.899	3972764	70192	2.898
Total		137072054		100.000

^{1}H NMR (400 MHz, DMSO-d_6) and ^{13}C NMR (101 MHz, DMSO-d_6) of 5d

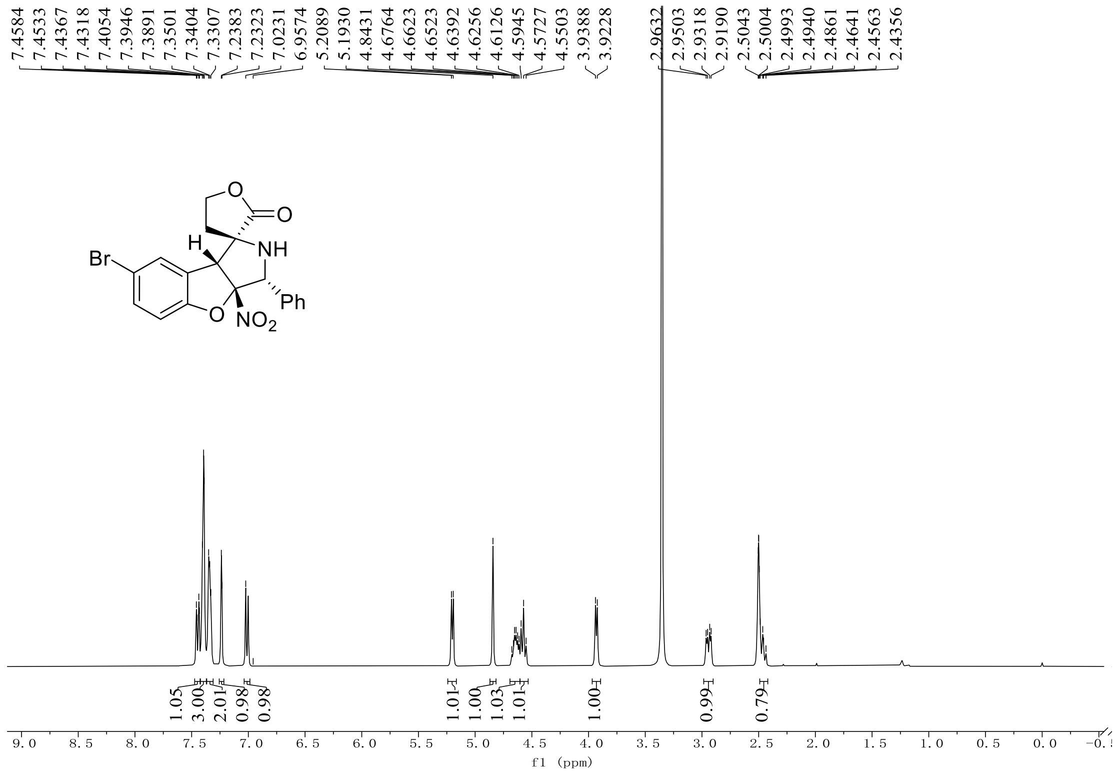

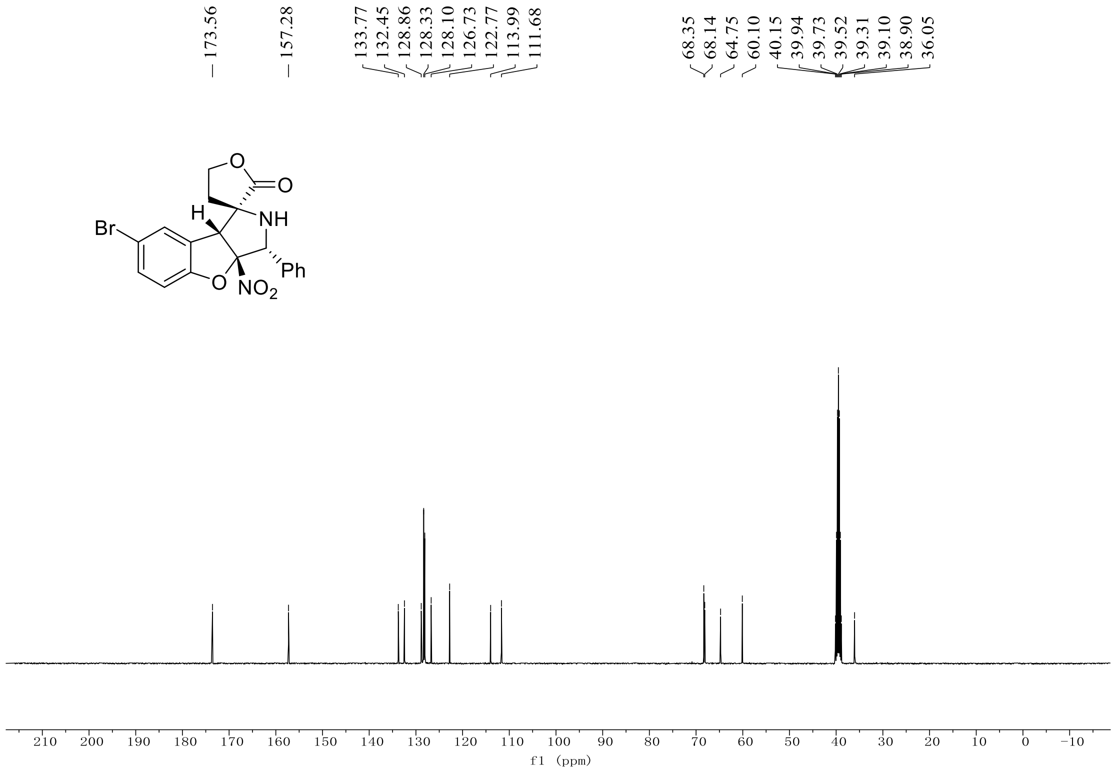

HPLC spectra of 5d

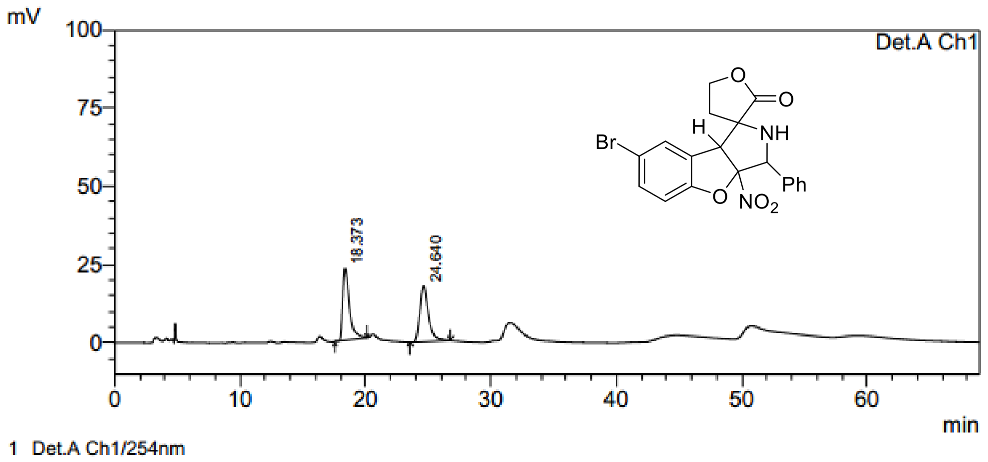

PeakTable

Detector A Ch1 254nm

Peak#	Ret. Time	Area	Height	Area %
1	18.373	848889	22893	50.076
2	24.640	846308	17761	49.924
Total		1695197		100.000

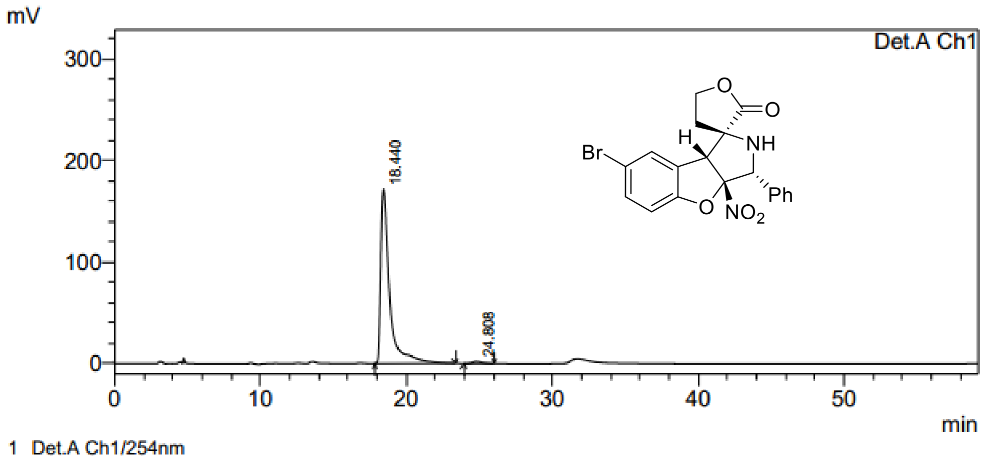

PeakTable

Detector A Ch1 254nm

Peak#	Ret. Time	Area	Height	Area %
1	18.440	7226885	171478	98.842
2	24.808	84673	1891	1.158
Total		7311558		100.000

^{1}H NMR (400 MHz, DMSO-d_6) and ^{13}C NMR (101 MHz, DMSO-d_6) of 5e

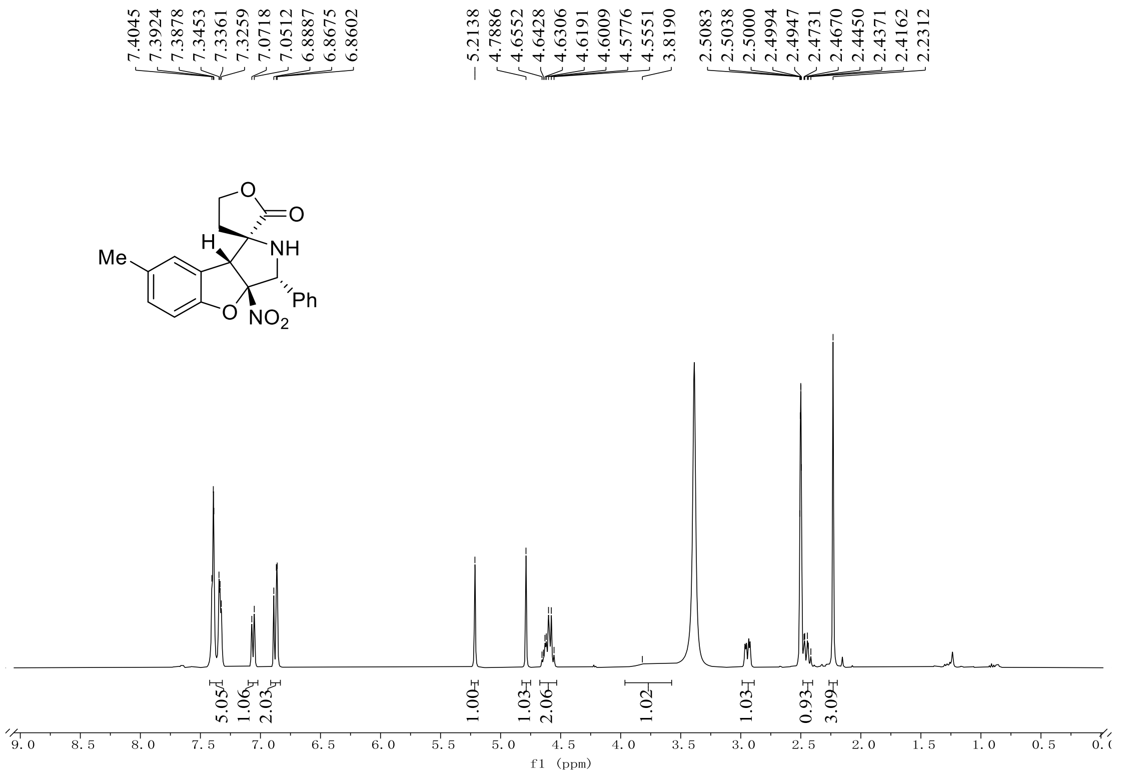

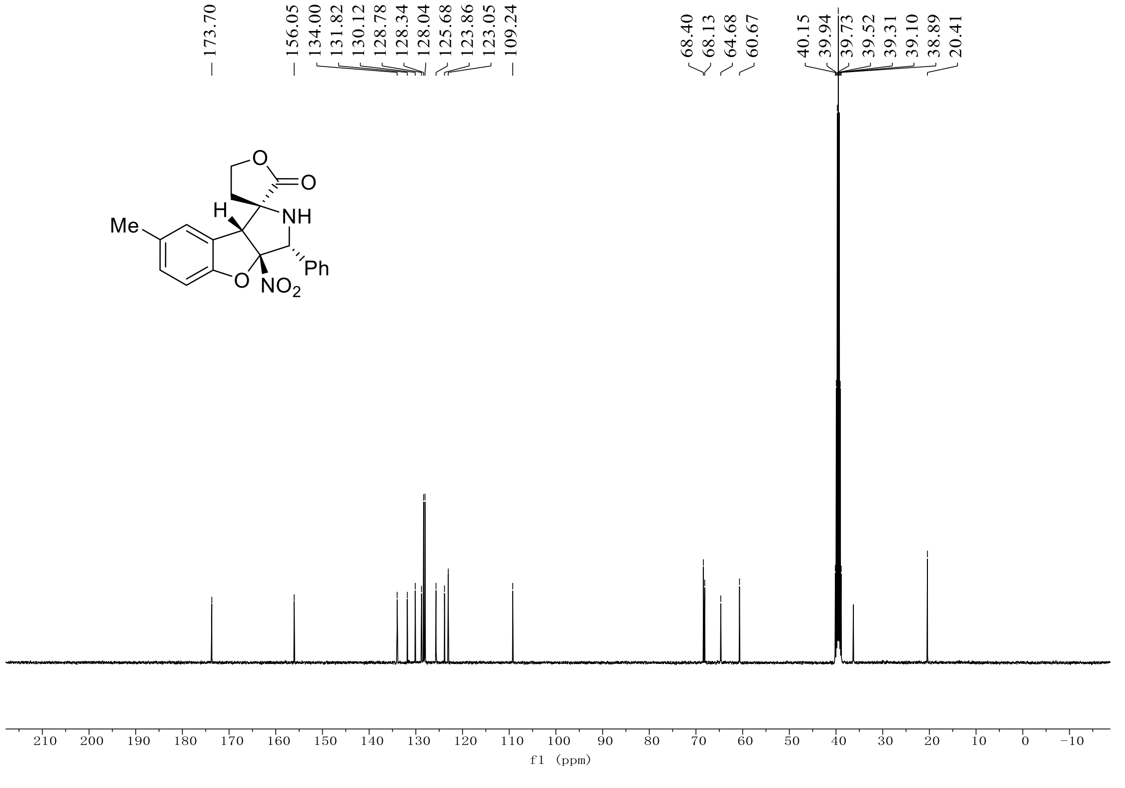

HPLC spectra of 5e

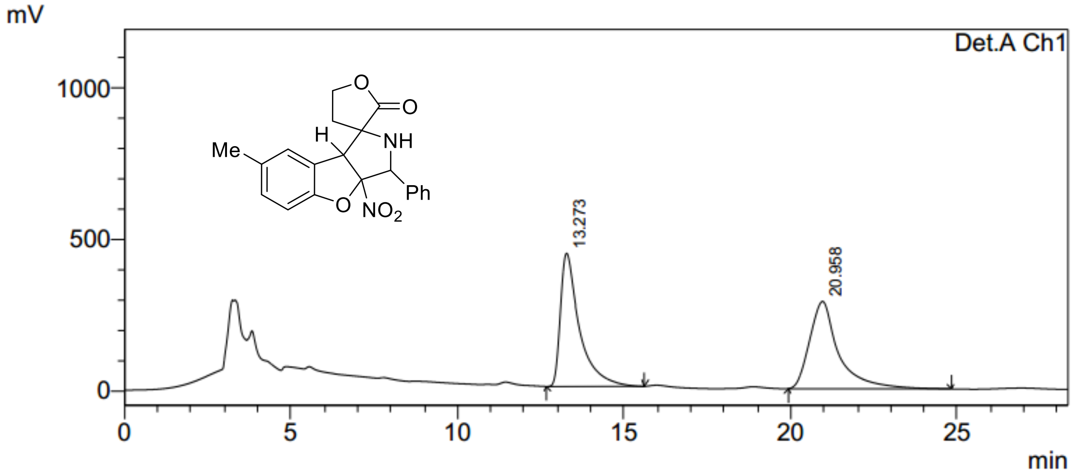

1 Det.A Ch1/220nm

PeakTable

Detector A Ch1 220nm

Peak#	Ret. Time	Area	Height	Area %
1	13.273	17555802	441957	49.835
2	20.958	17671785	290162	50.165
Total		35227587		100.000

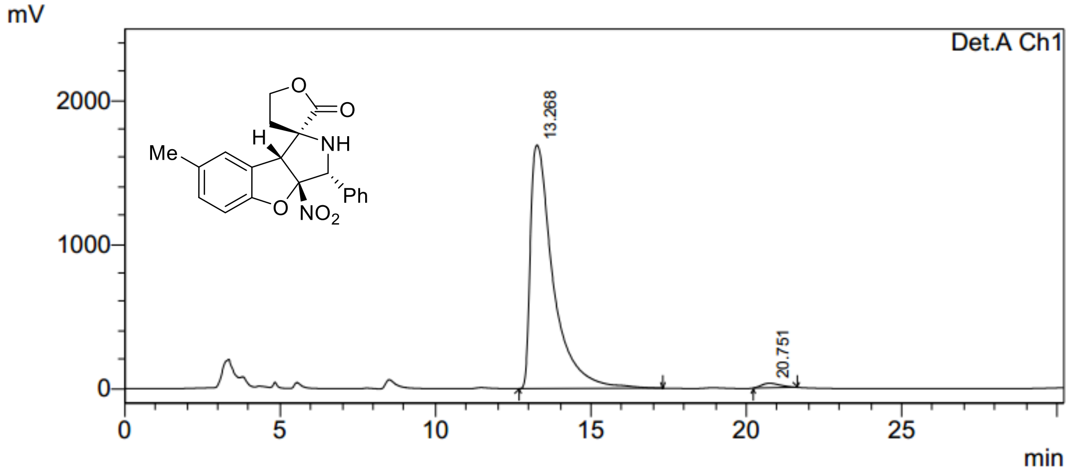

1 Det.A Ch1/220nm

PeakTable

Detector A Ch1 220nm

Peak#	Ret. Time	Area	Height	Area %
1	13.268	86122036	1689286	98.489
2	20.751	1321531	31410	1.511
Total		87443567		100.000

^{1}H NMR (400 MHz, DMSO-d_6) and ^{13}C NMR (101 MHz, DMSO-d_6) of 5f

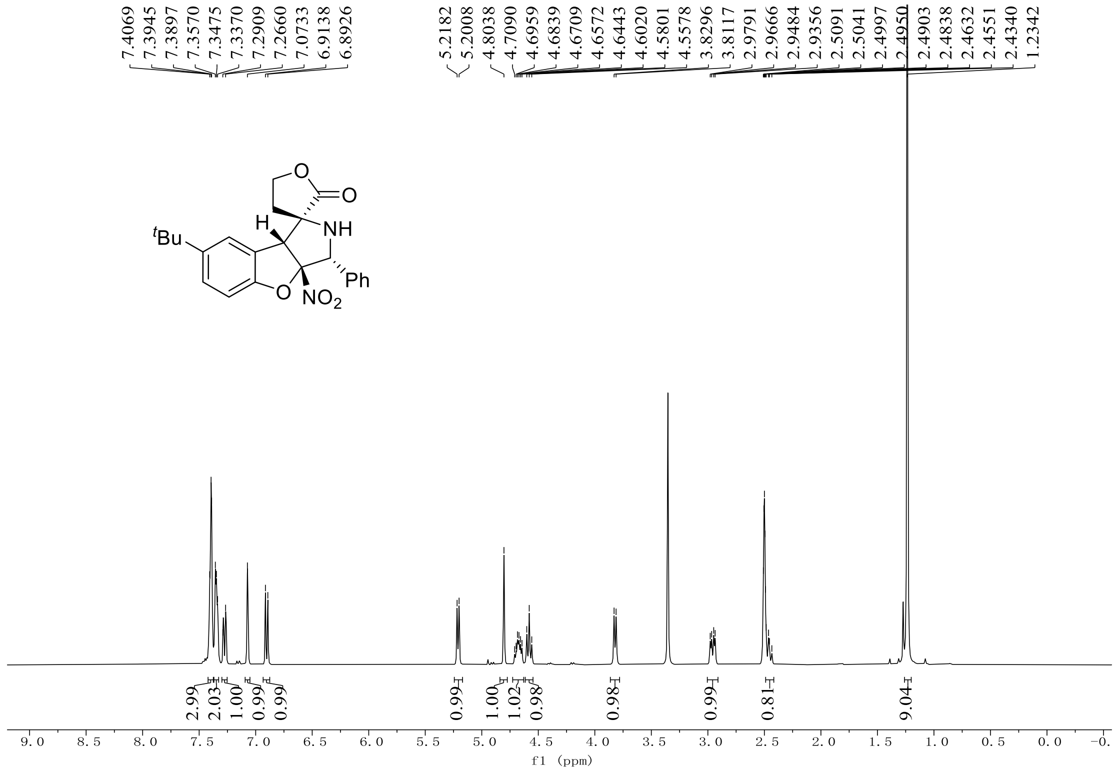

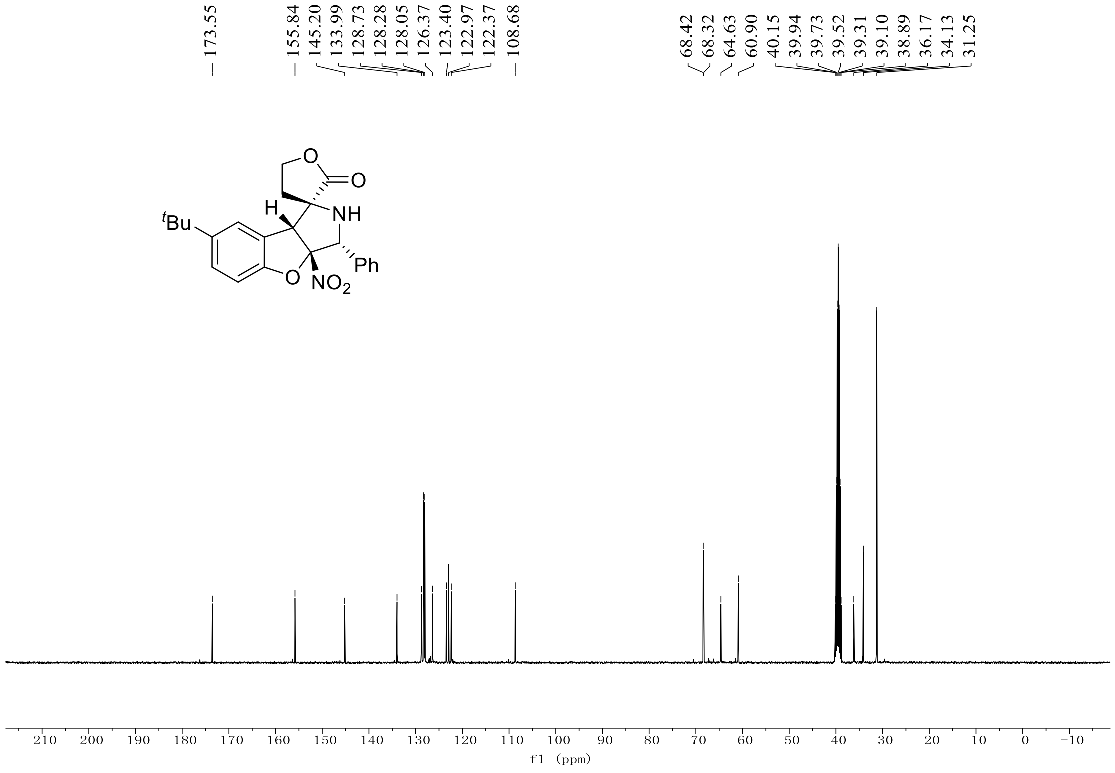

HPLC spectra of 5f

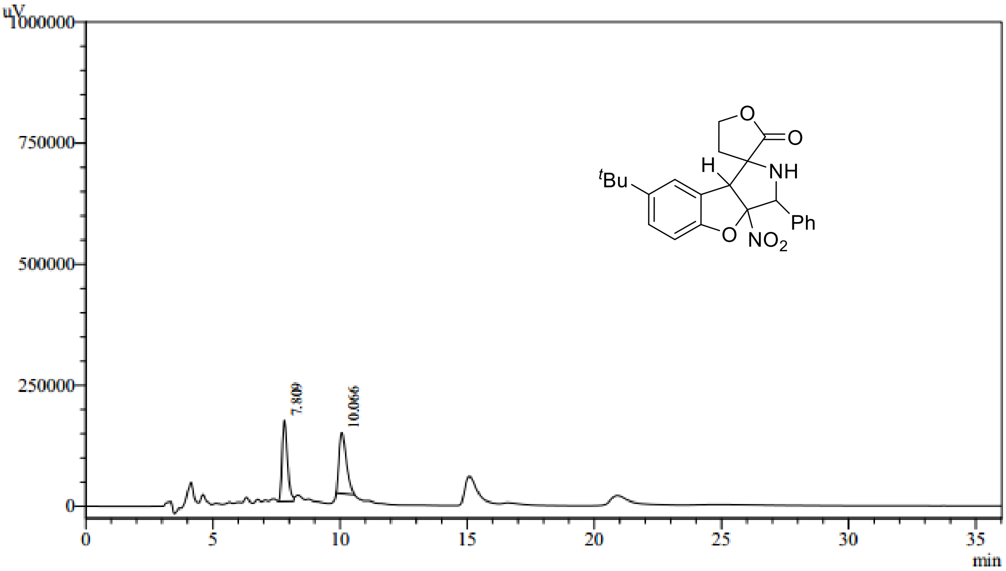

1 Det.A Ch1 / 254nm

Detector A Ch1 254nm

Peak#	Ret. Time	Area	Height	Area %	Height %
1	7.809	2453116	167288	49.261	57.038
2	10.066	2526767	126003	50.739	42.962
Total		4979884	293291	100.000	100.000

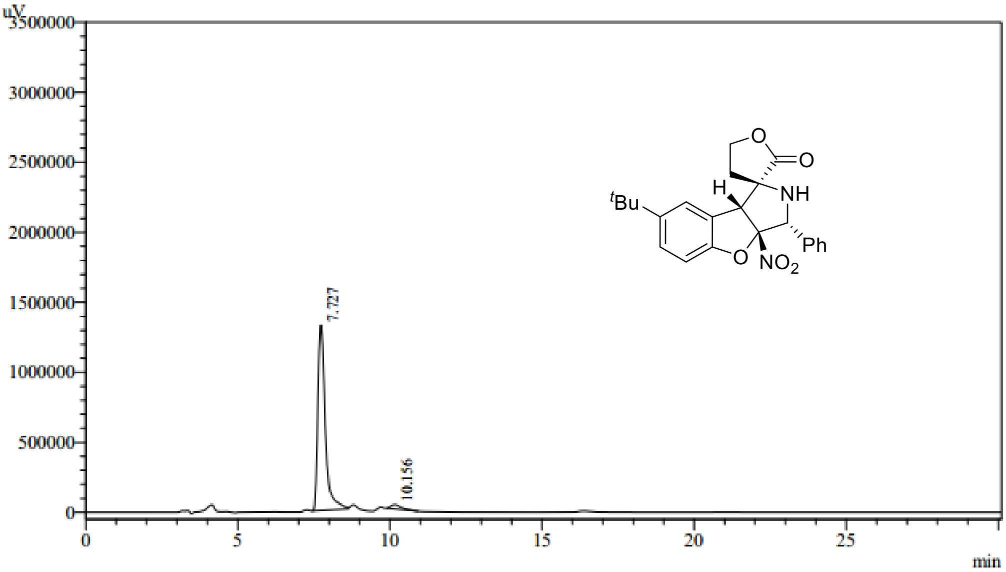

1 Det.A Ch1 / 254nm

Detector A Ch1 254nm

Peak#	Ret. Time	Area	Height	Area %	Height %
1	7.727	22679021	1324659	96.750	97.743
2	10.156	761948	30585	3.250	2.257
Total		23440969	1355243	100.000	100.000

^{1}H NMR (400 MHz, DMSO-d_6) and ^{13}C NMR (101 MHz, DMSO-d_6) of 5g

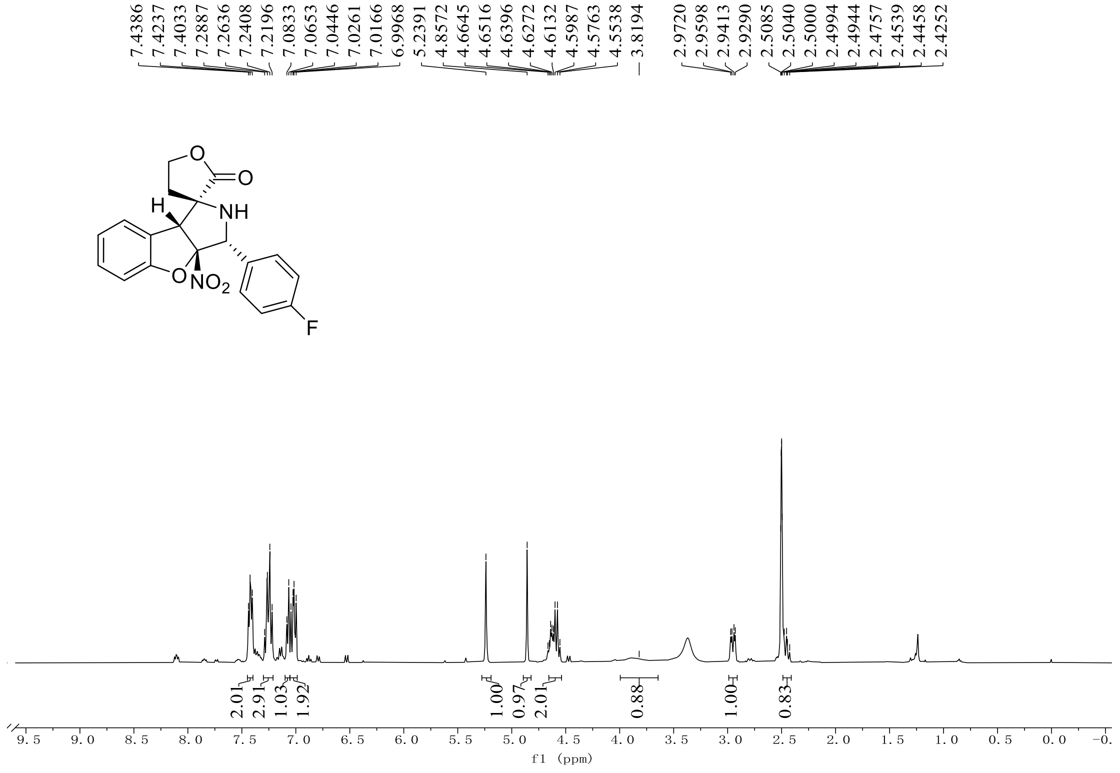

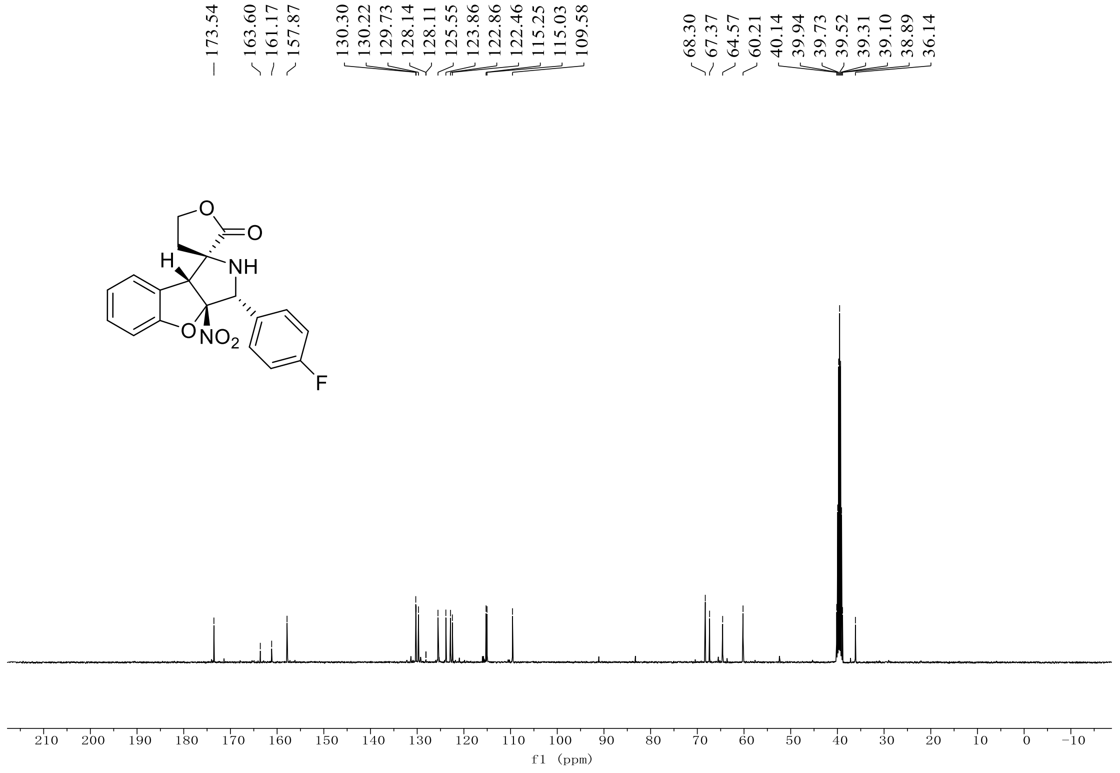

HPLC spectra of 5g

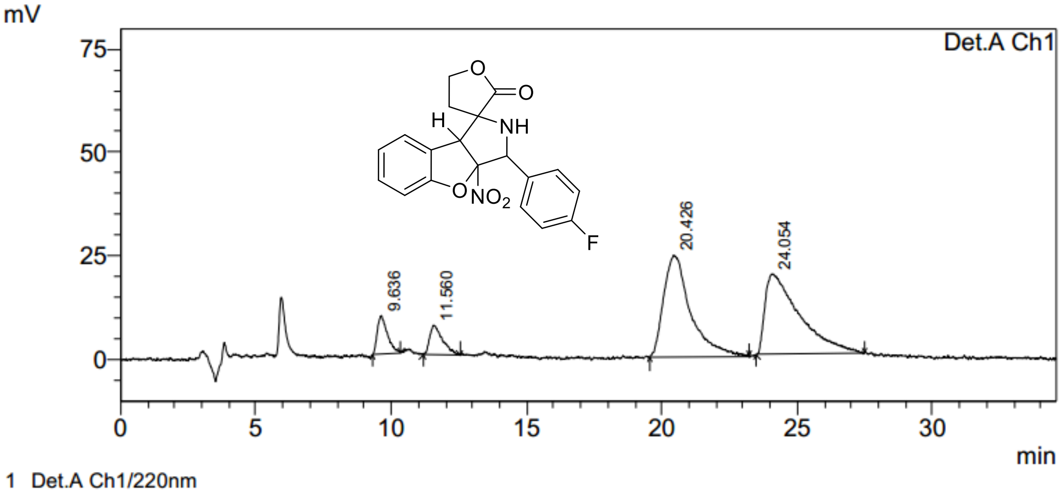

PeakTable

Detector A Ch1 220nm

Peak#	Ret. Time	Area	Height	Area %
1	9.636	238985	9296	6.142
2	11.560	233494	7062	6.001
3	20.426	1711645	24610	43.992
4	24.054	1706658	19330	43.864
Total		3890783		100.000

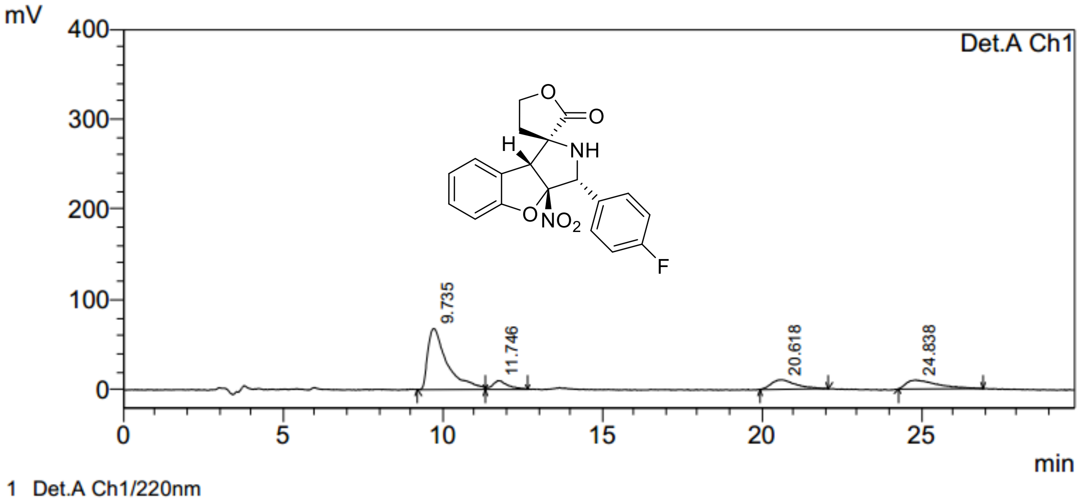

PeakTable

Detector A Ch1 220nm

Peak#	Ret. Time	Area	Height	Area %
1	9.735	2865620	67686	64.614
2	11.746	328069	9600	7.397
3	20.618	560612	10571	12.641
4	24.838	680706	10088	15.348
Total		4435006		100.000

^{1}H NMR (400 MHz, DMSO-d_6) and ^{13}C NMR (101 MHz, DMSO-d_6) of 5h

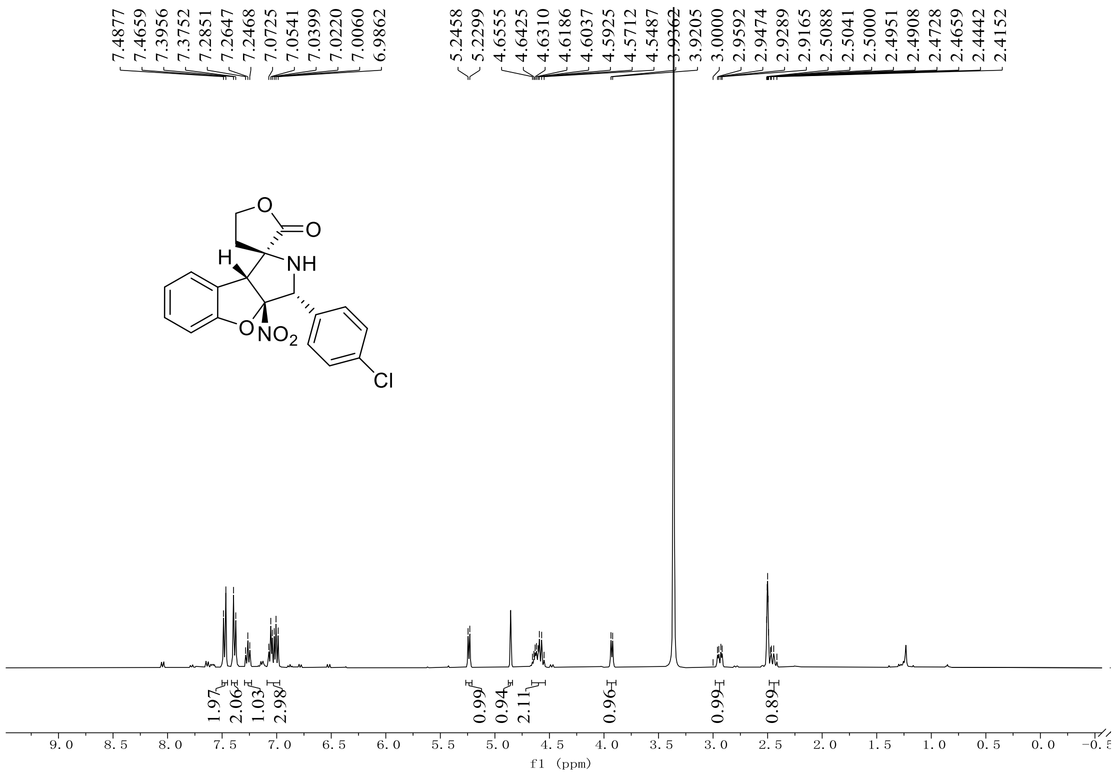

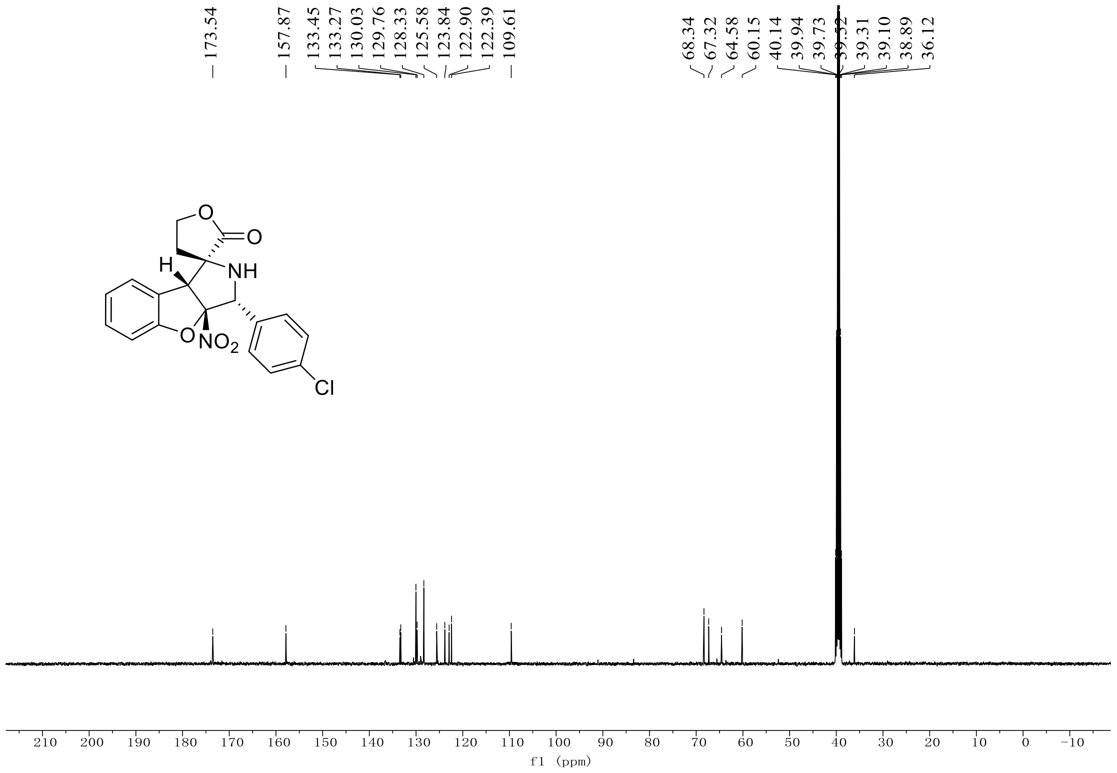

HPLC spectra of 5h

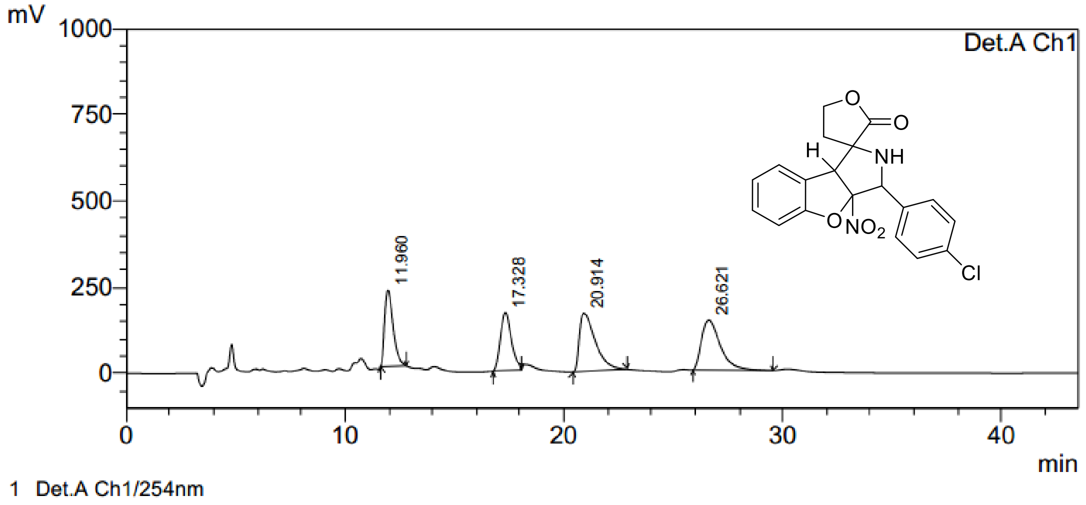

PeakTable

Detector A Ch1 254nm

Peak#	Ret. Time	Area	Height	Area %
1	11.960	5990811	221406	20.575
2	17.328	5885122	168221	20.212
3	20.914	8564342	168395	29.414
4	26.621	8676051	145642	29.798
Total		29116327		100.000

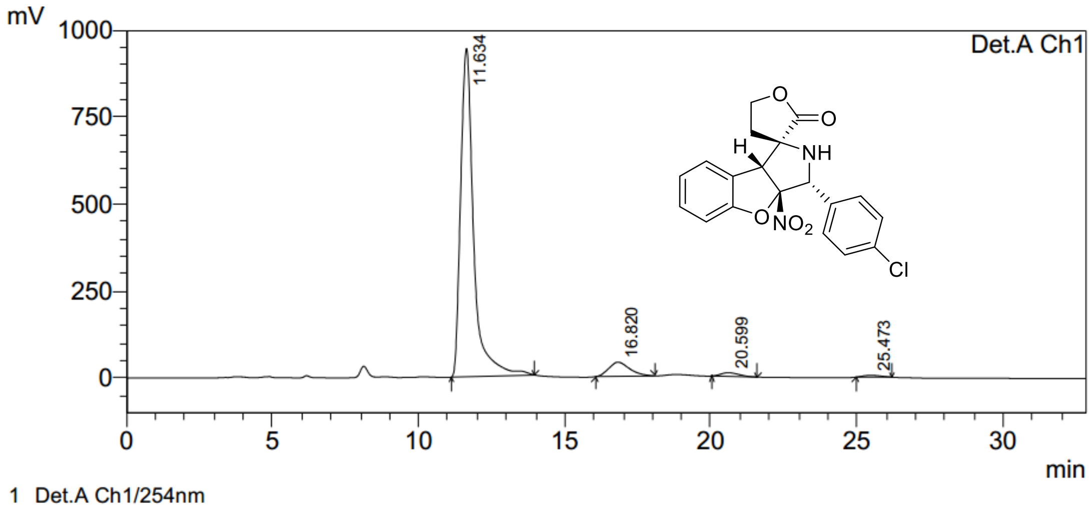

PeakTable

Detector A Ch1 254nm

Peak#	Ret. Time	Area	Height	Area %
1	11.634	29274414	942273	91.489
2	16.820	2022844	40709	6.322
3	20.599	470313	10734	1.470
4	25.473	230147	5326	0.719
Total		31997718		100.000

^{1}H NMR (400 MHz, DMSO-d_6) and ^{13}C NMR (101 MHz, DMSO-d_6) of 5i

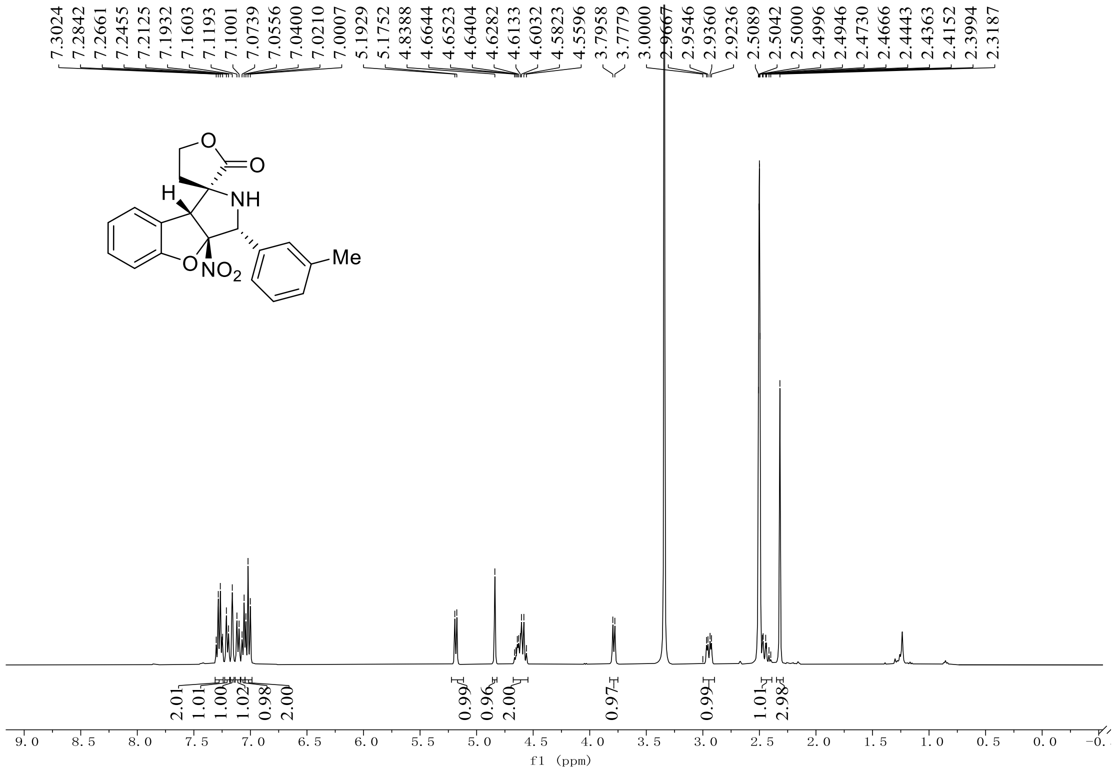

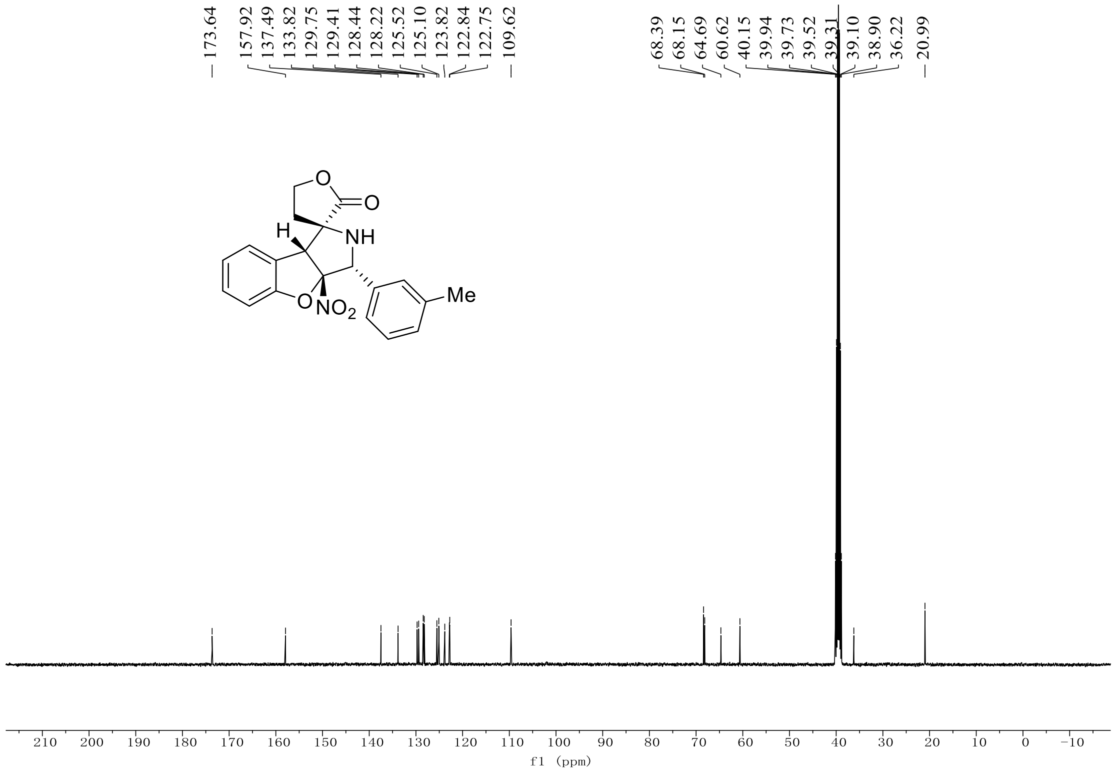

HPLC spectra of 5i

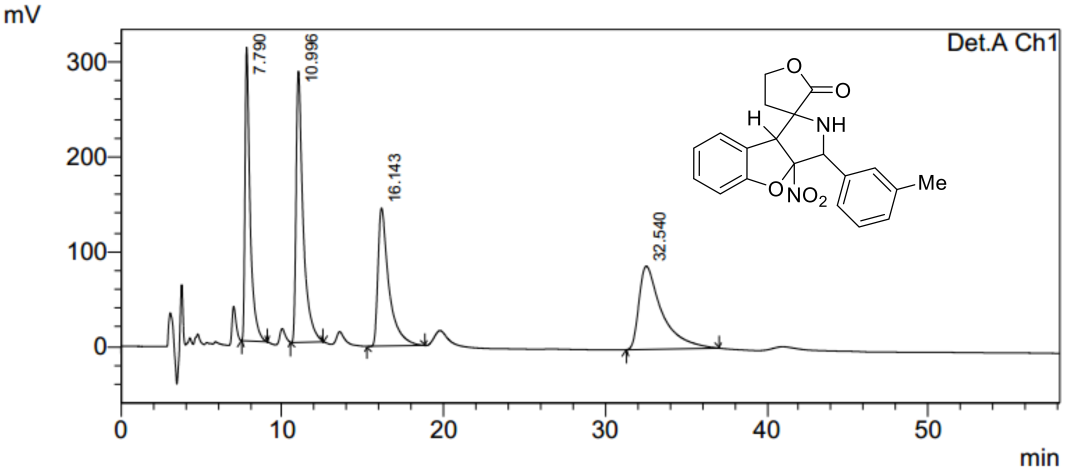

1 Det.A Ch1/220nm

PeakTable

Detector A Ch1 220nm

Peak#	Ret. Time	Area	Height	Area %
1	7.790	6816273	310225	21.888
2	10.996	8744705	286499	28.080
3	16.143	6863027	145949	22.038
4	32.540	8718174	87998	27.995
Total		31142180		100.000

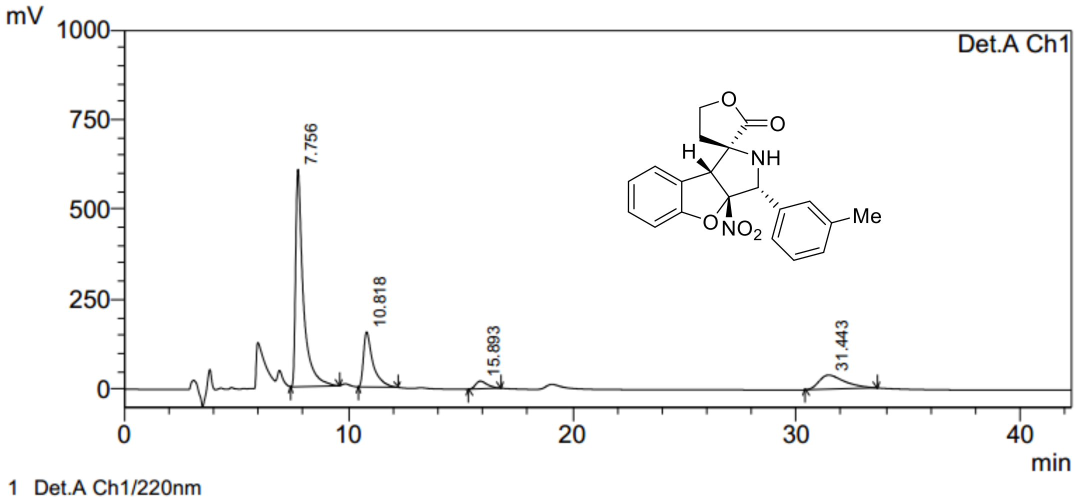

1 Det.A Ch1/220nm

PeakTable

Detector A Ch1 220nm

Peak#	Ret. Time	Area	Height	Area %
1	7.756	14458743	604517	63.301
2	10.818	4492765	152979	19.669
3	15.893	769588	21286	3.369
4	31.443	3120191	39135	13.660
Total		22841288		100.000

^{1}H NMR (400 MHz, DMSO-d_6) and ^{13}C NMR (101 MHz, DMSO-d_6) of 5j

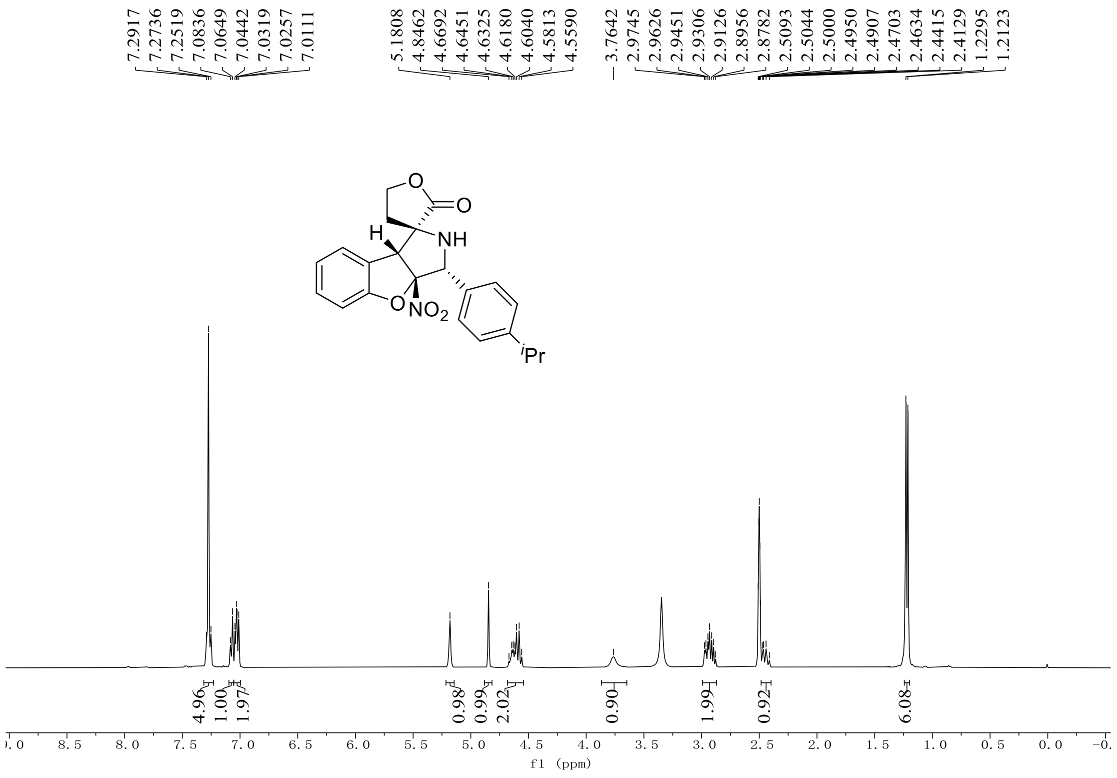

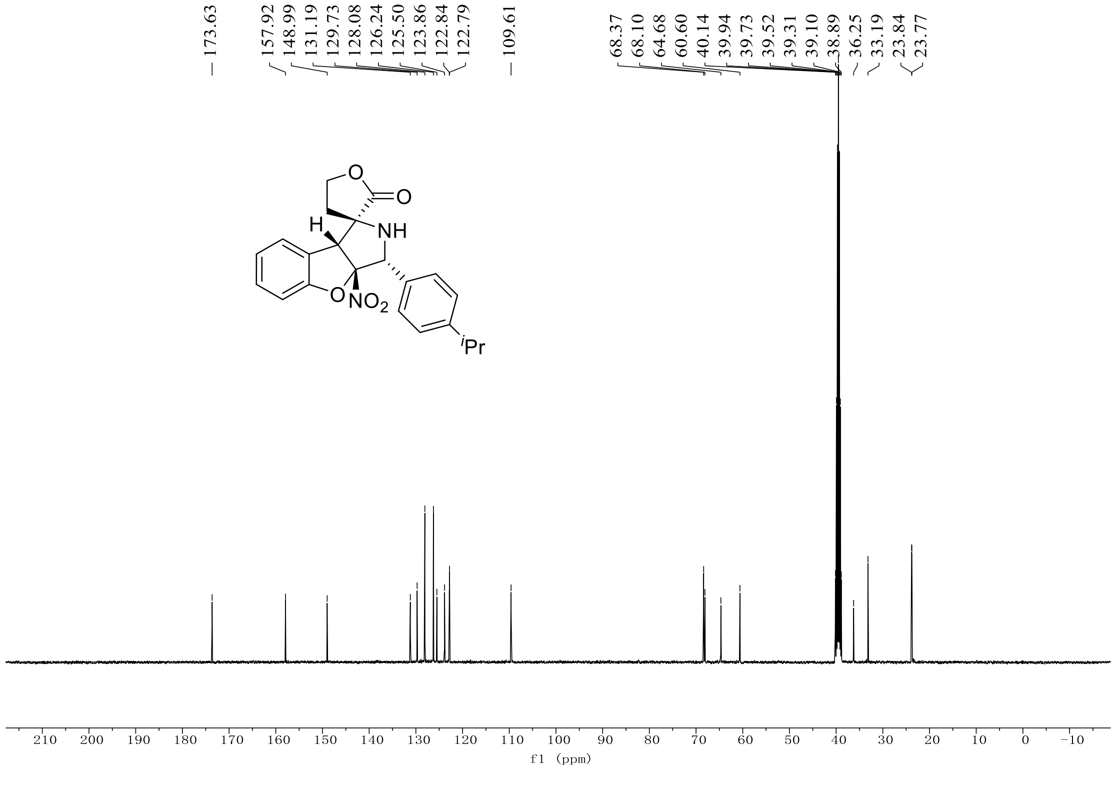

HPLC spectra of 5j

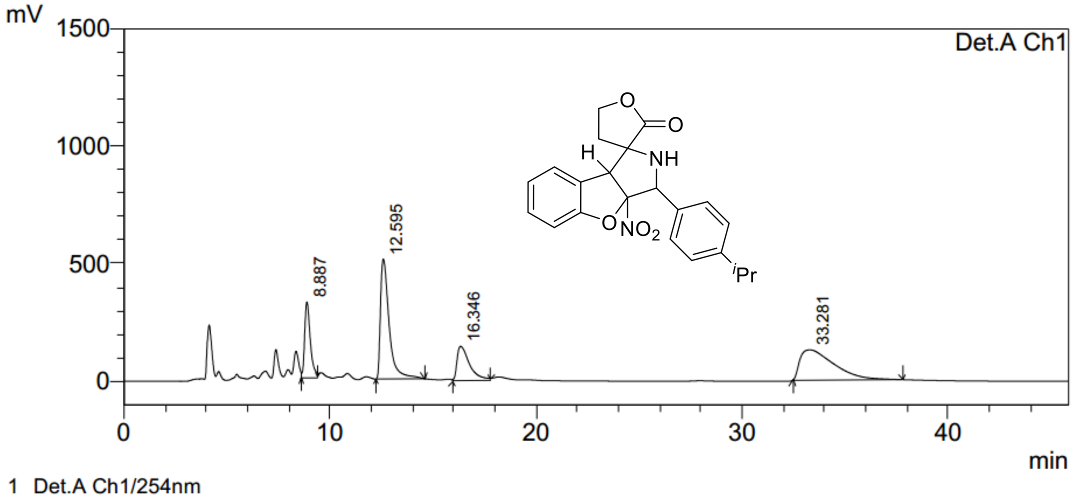

PeakTable

Detector A Ch1 254nm

Peak#	Ret. Time	Area	Height	Area %
1	8.887	6199662	322244	14.657
2	12.595	14964963	509226	35.380
3	16.346	6178162	145436	14.606
4	33.281	14955240	129398	35.357
Total		42298027		100.000

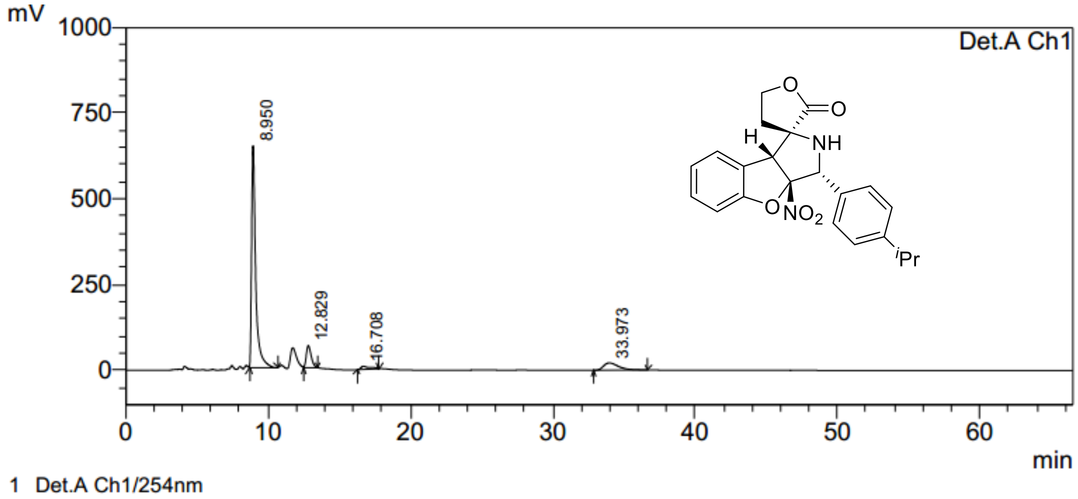

PeakTable

Detector A Ch1 254nm

Peak#	Ret. Time	Area	Height	Area %
1	8.950	12934559	646595	78.503
2	12.829	1548732	64334	9.400
3	16.708	436236	9232	2.648
4	33.973	1557025	21089	9.450
Total		16476552		100.000

^{1}H NMR (400 MHz, DMSO-d$_6$) and ^{13}C NMR (101 MHz, DMSO-d$_6$) of 5k

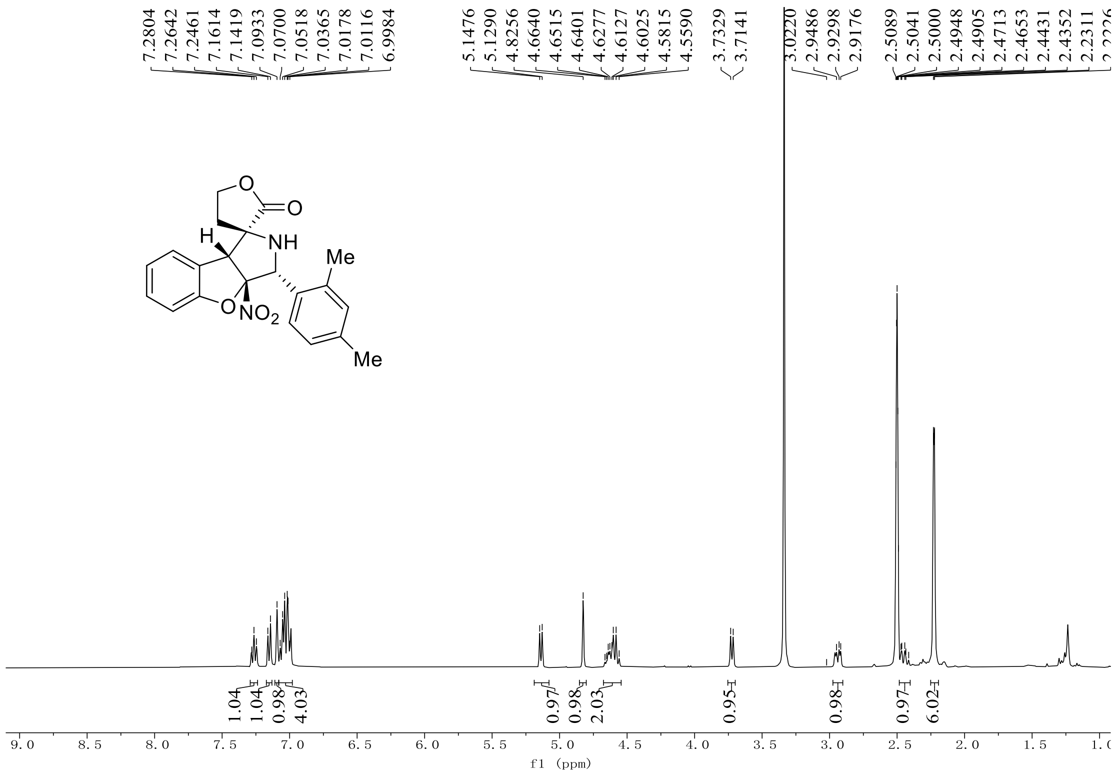

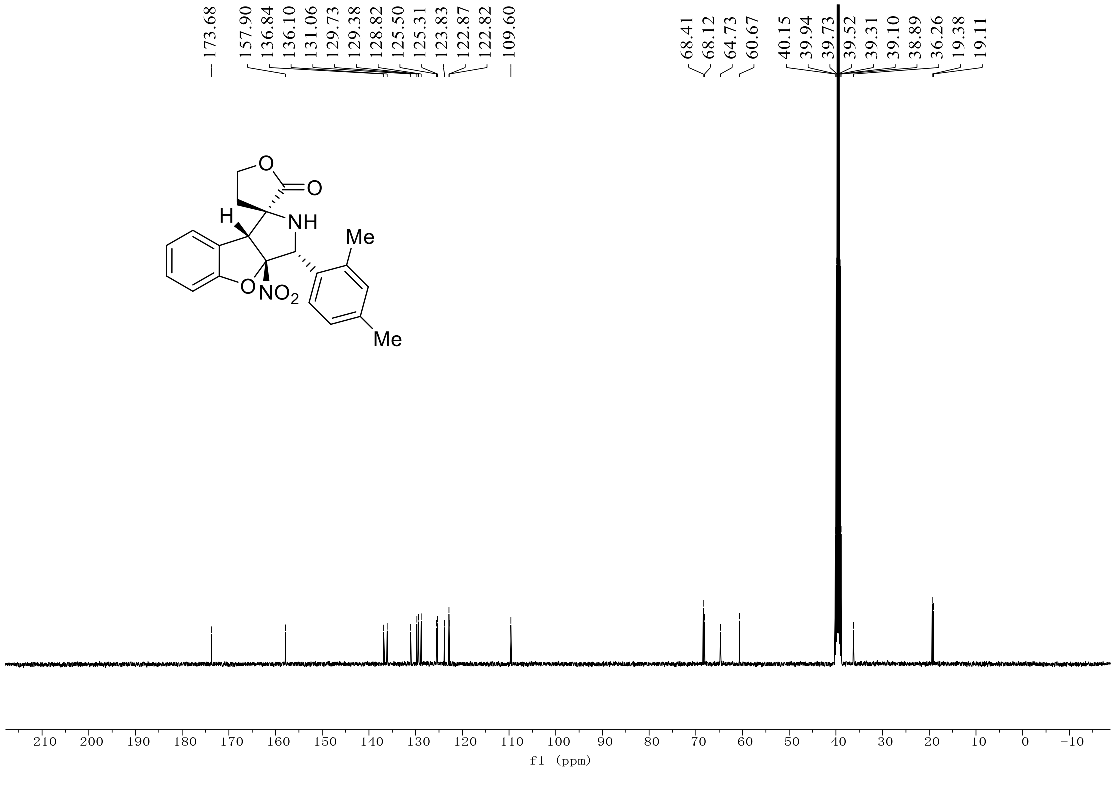

HPLC spectra of 5k

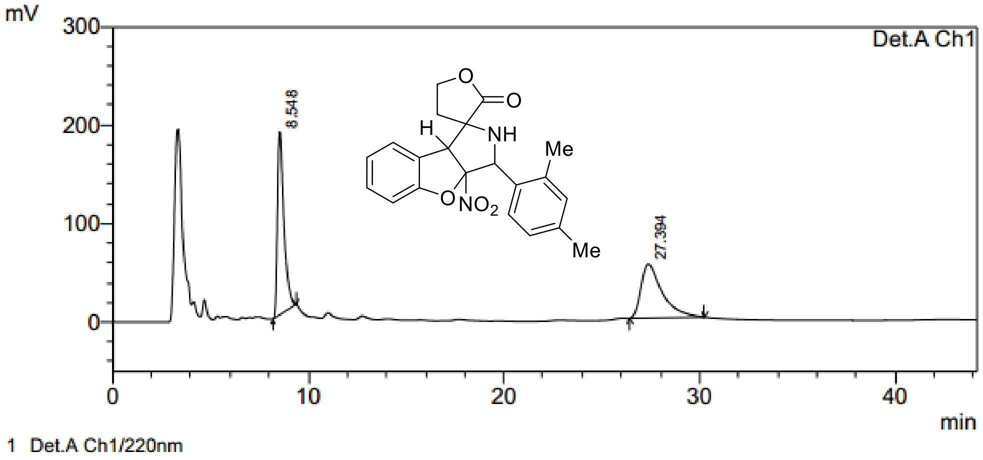

PeakTable

Detector A Ch1 220nm

Peak#	Ret. Time	Area	Height	Area %
1	8.548	4251384	185739	50.773
2	27.394	4121976	54607	49.227
Total		8373360		100.000

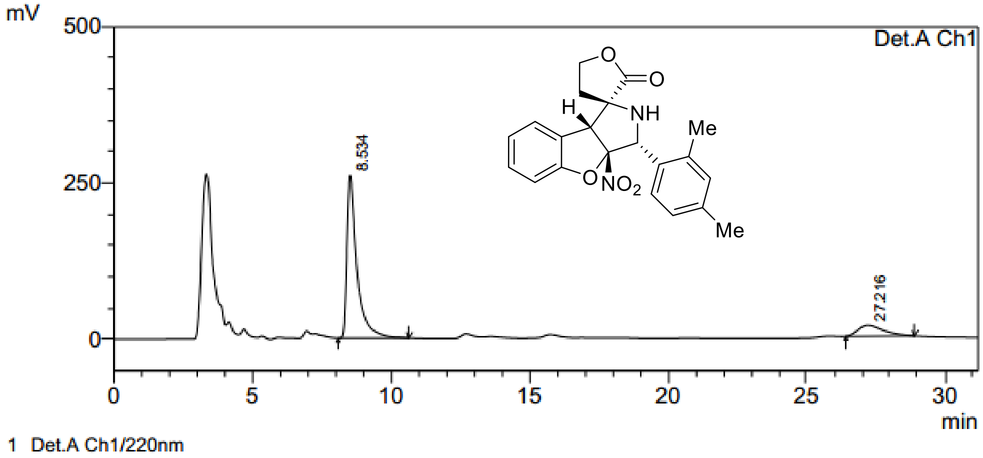

PeakTable

Detector A Ch1 220nm

Peak#	Ret. Time	Area	Height	Area %
1	8.534	6506236	259619	85.117
2	27.216	1137678	17733	14.883
Total		7643915		100.000

^{1}H NMR (400 MHz, DMSO-d_6) and ^{13}C NMR (101 MHz, DMSO-d_6) of 5l

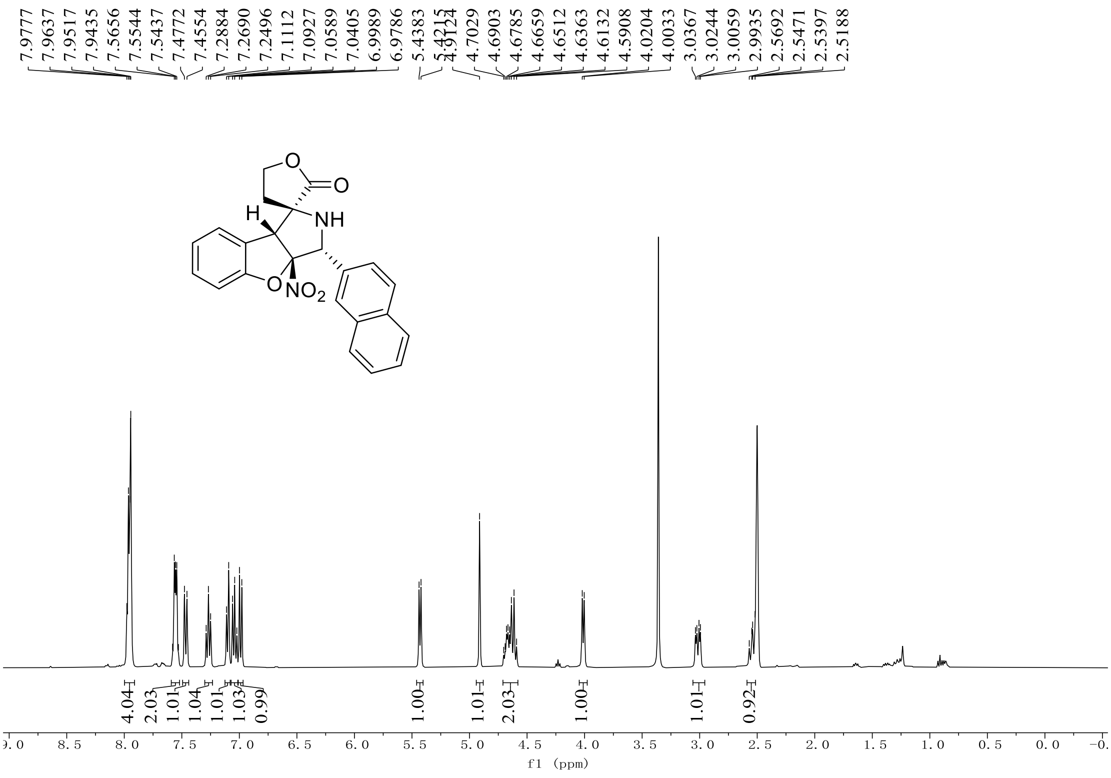

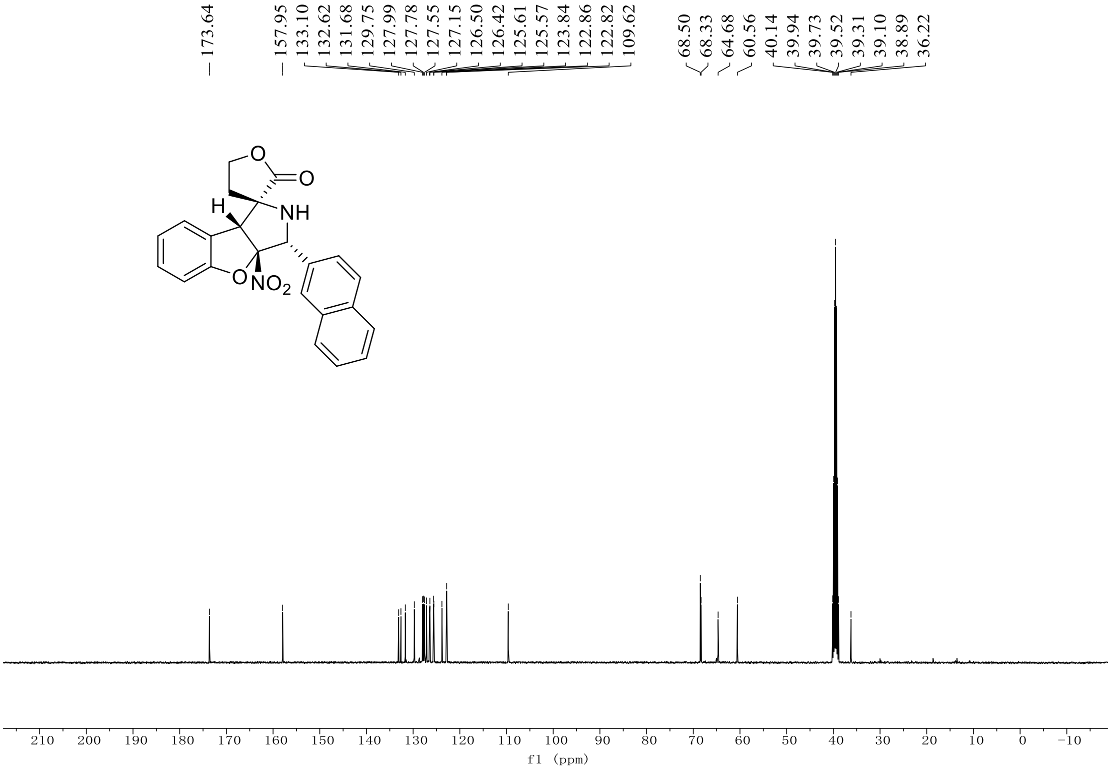

HPLC spectra of 5l

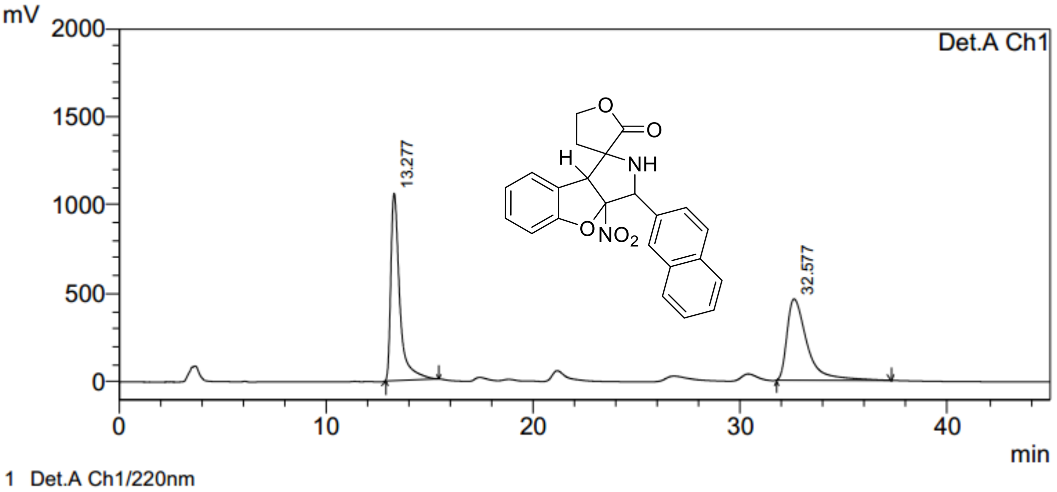

PeakTable

Detector A Ch1 220nm

Peak#	Ret. Time	Area	Height	Area %
1	13.277	31836970	1059176	50.826
2	32.577	30802253	458913	49.174
Total		62639222		100.000

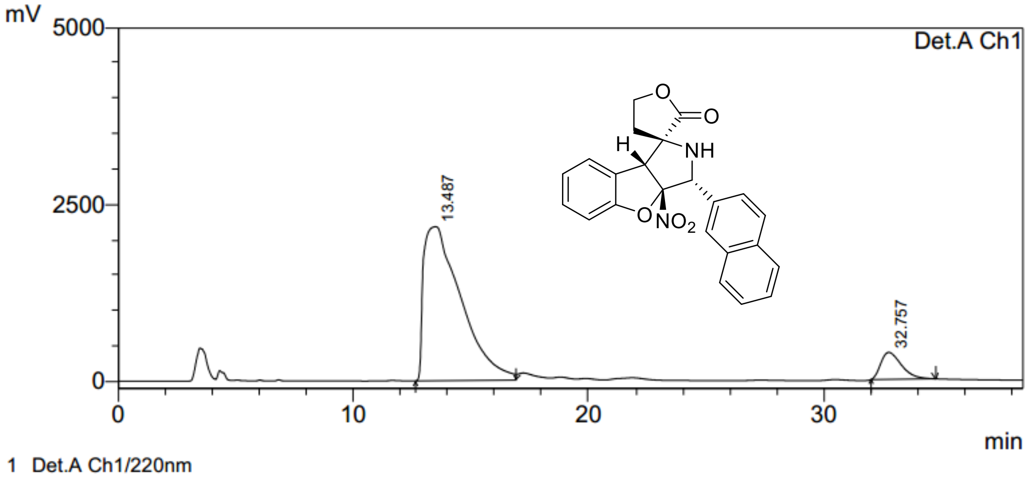

PeakTable

Detector A Ch1 220nm

Peak#	Ret. Time	Area	Height	Area %
1	13.487	241466470	2178456	91.216
2	32.757	23252657	381101	8.784
Total		264719127		100.000

^{1}H NMR (400 MHz, DMSO-d_6) and ^{13}C NMR (101 MHz, DMSO-d_6) of 5m

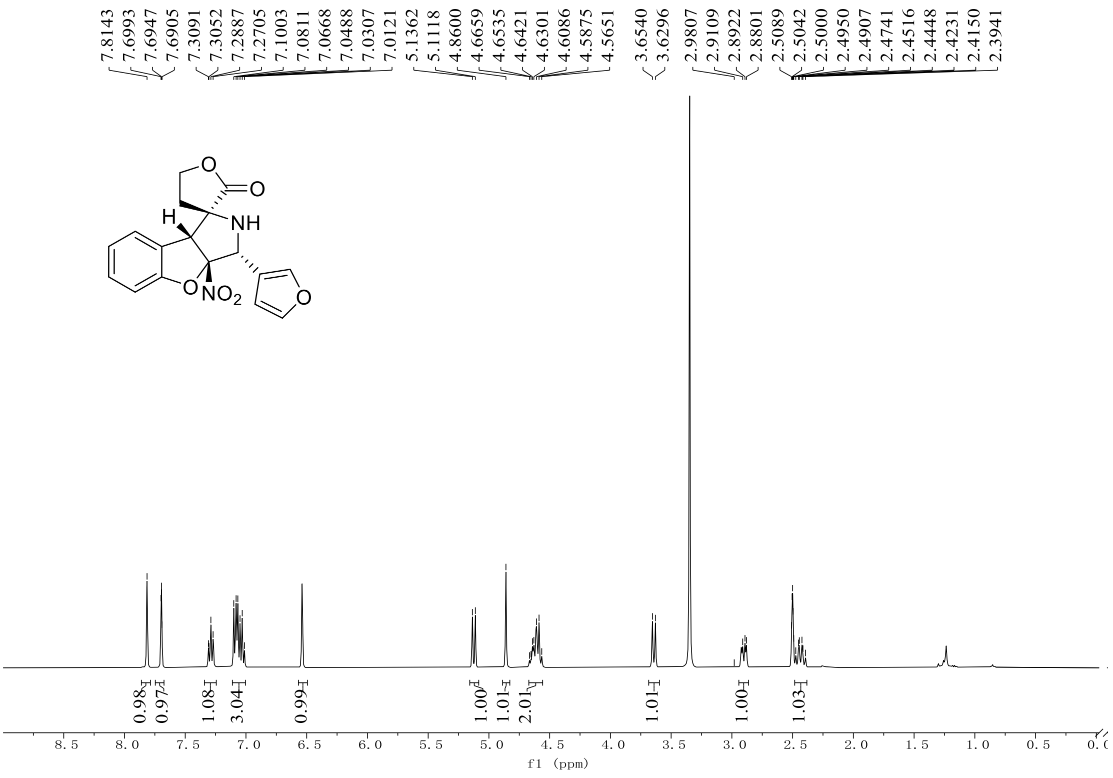

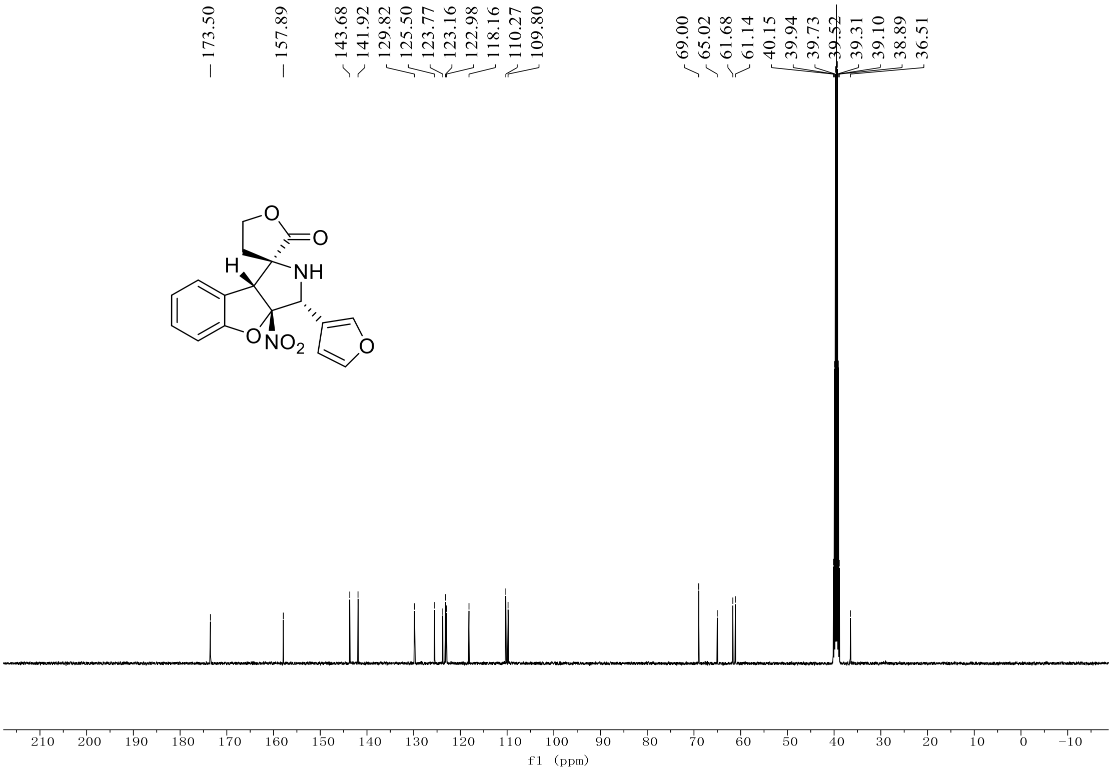

HPLC spectra of 5m

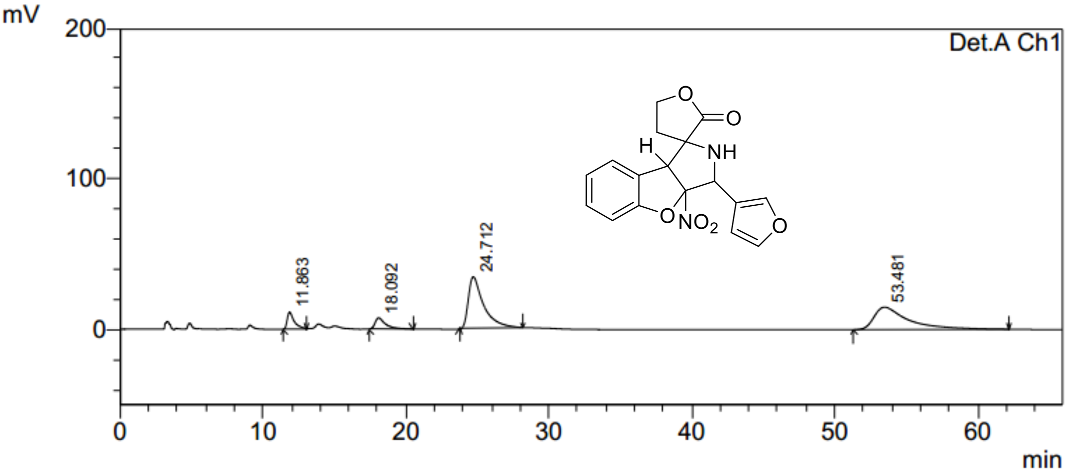

1 Det.A Ch1/254nm

PeakTable

Detector A Ch1 254nm

Peak#	Ret. Time	Area	Height	Area %
1	11.863	385790	11203	6.696
2	18.092	365807	7320	6.349
3	24.712	2516724	34129	43.683
4	53.481	2493059	14877	43.272
Total		5761380		100.000

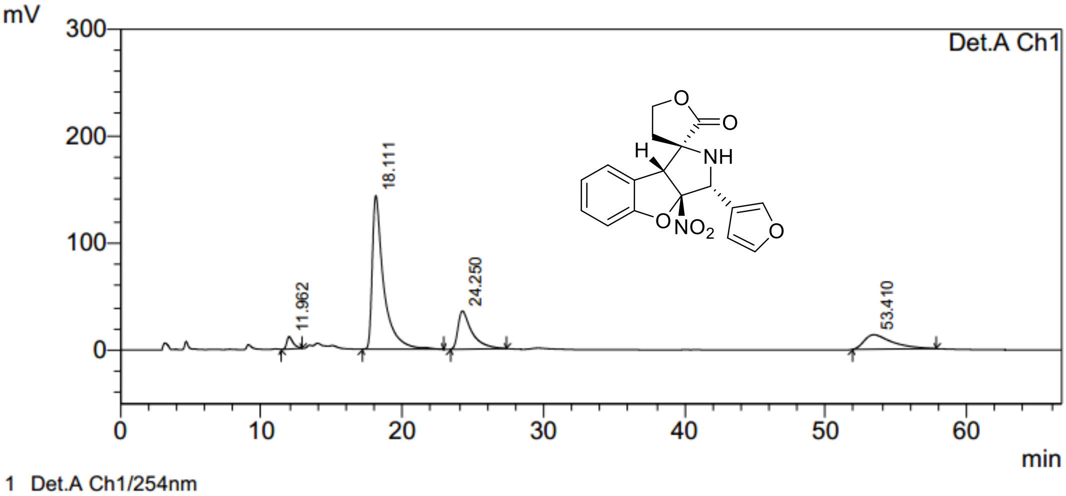

1 Det.A Ch1/254nm

PeakTable

Detector A Ch1 254nm

Peak#	Ret. Time	Area	Height	Area %
1	11.962	360315	11527	2.827
2	18.111	8050330	143178	63.165
3	24.250	2444755	35613	19.182
4	53.410	1889505	13576	14.826
Total		12744905		100.000

^{1}H NMR (400 MHz, DMSO-d$_6$) and ^{13}C NMR (101 MHz, DMSO-d$_6$) of 6

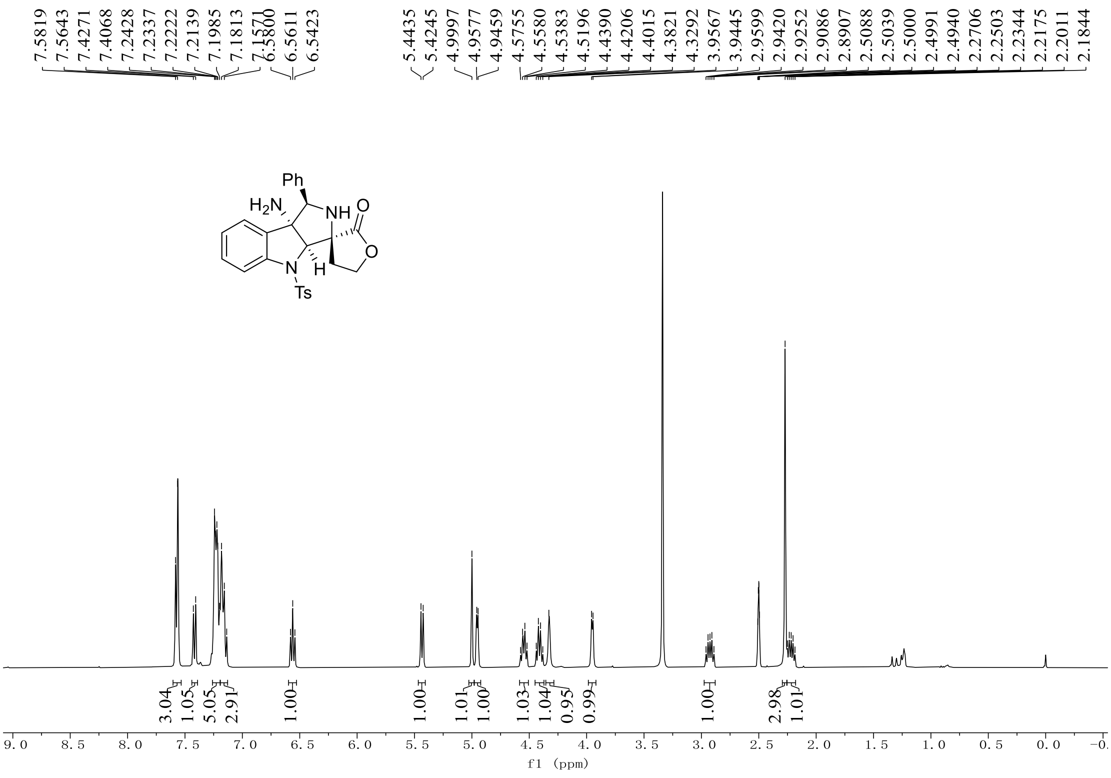

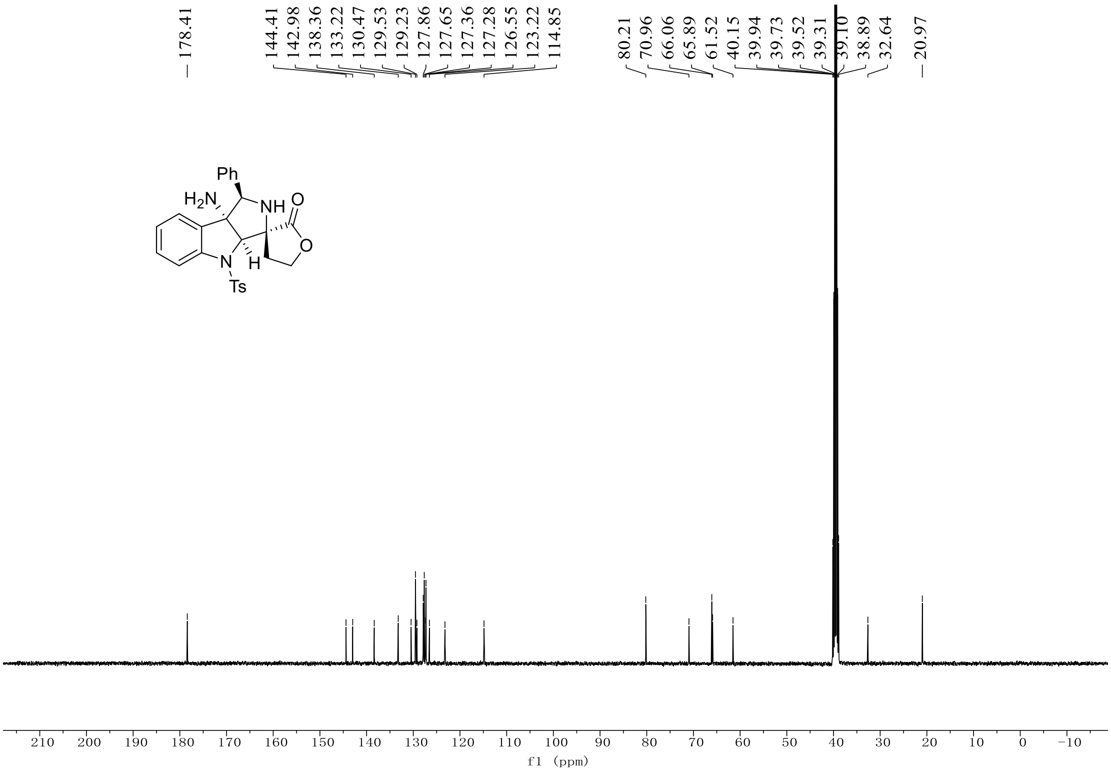

HPLC spectra of 6

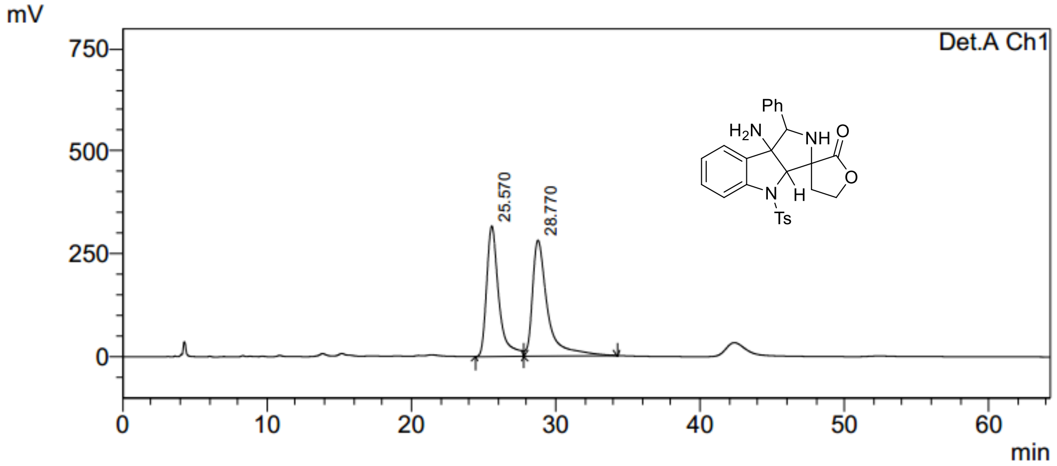

1 Det.A Ch1/254nm

PeakTable

Detector A Ch1 254nm

Peak#	Ret. Time	Area	Height	Area %
1	25.570	18684948	317684	47.597
2	28.770	20571504	282228	52.403
Total		39256452		100.000

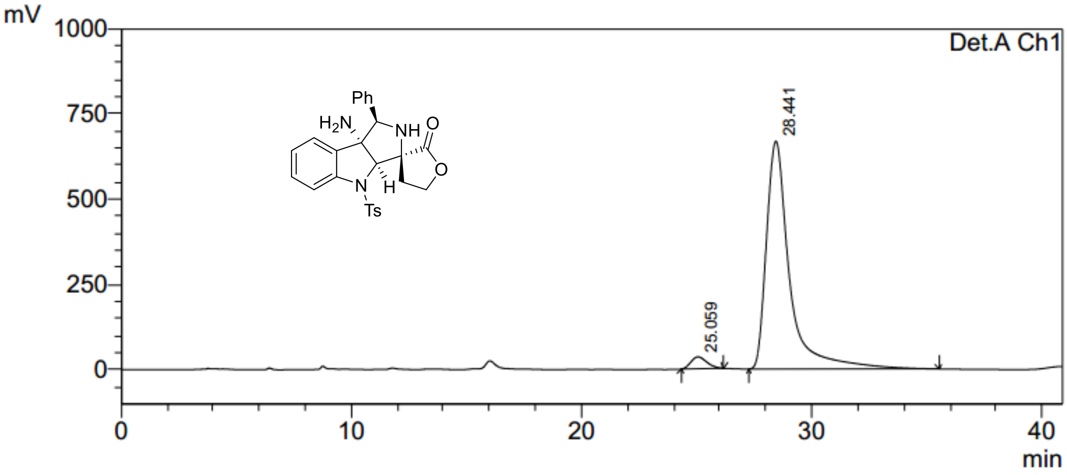

1 Det.A Ch1/254nm

PeakTable

Detector A Ch1 254nm

Peak#	Ret. Time	Area	Height	Area %
1	25.059	1698956	35151	3.499
2	28.441	46858420	667243	96.501
Total		48557376		100.000

^{1}H NMR (400 MHz, DMSO-d$_6$) and ^{13}C NMR (101 MHz, DMSO-d$_6$) of 7

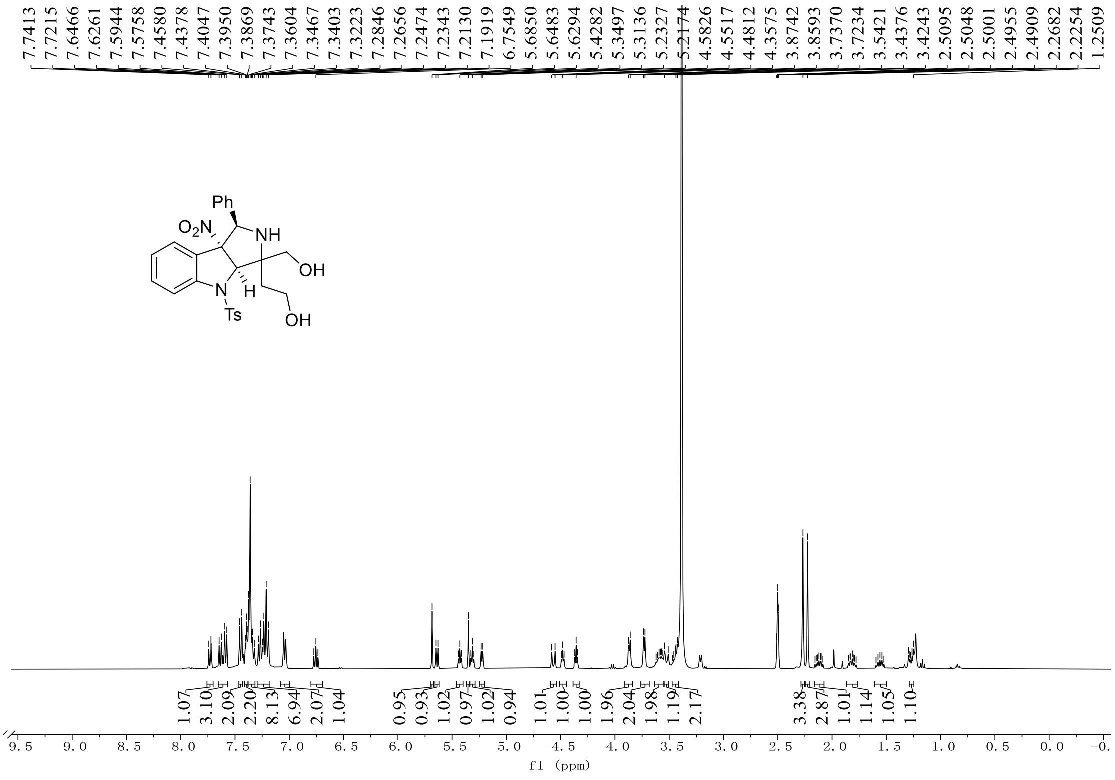

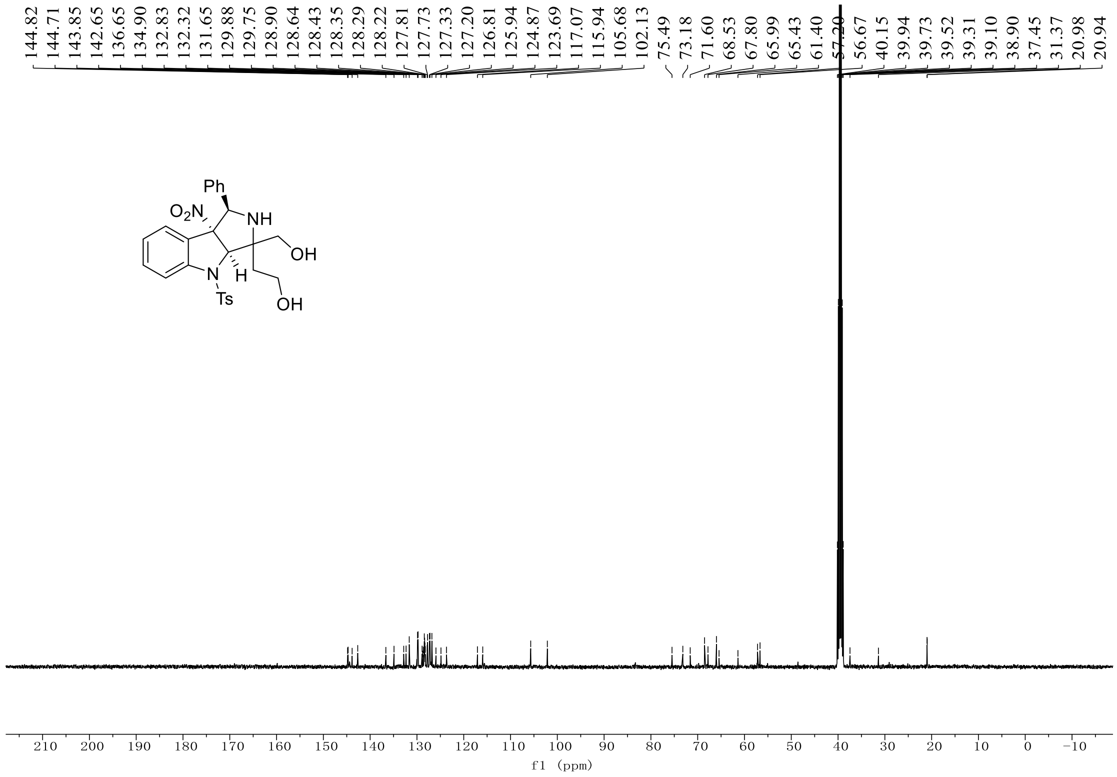

HPLC spectra of 7

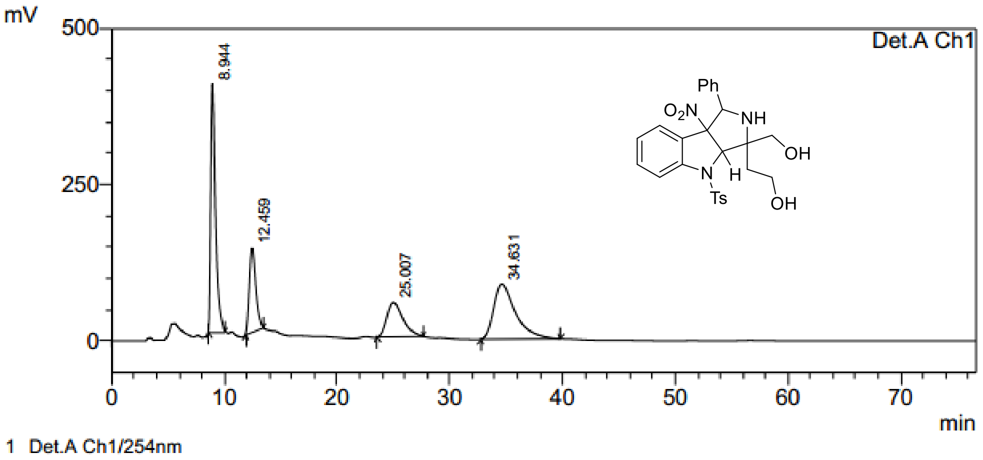

PeakTable

Detector A Ch1 254nm

Peak#	Ret. Time	Area	Height	Area %
1	8.944	11863673	397535	34.221
2	12.459	5395629	134414	15.564
3	25.007	5509068	54933	15.891
4	34.631	11899582	88181	34.324
Total		34667953		100.000

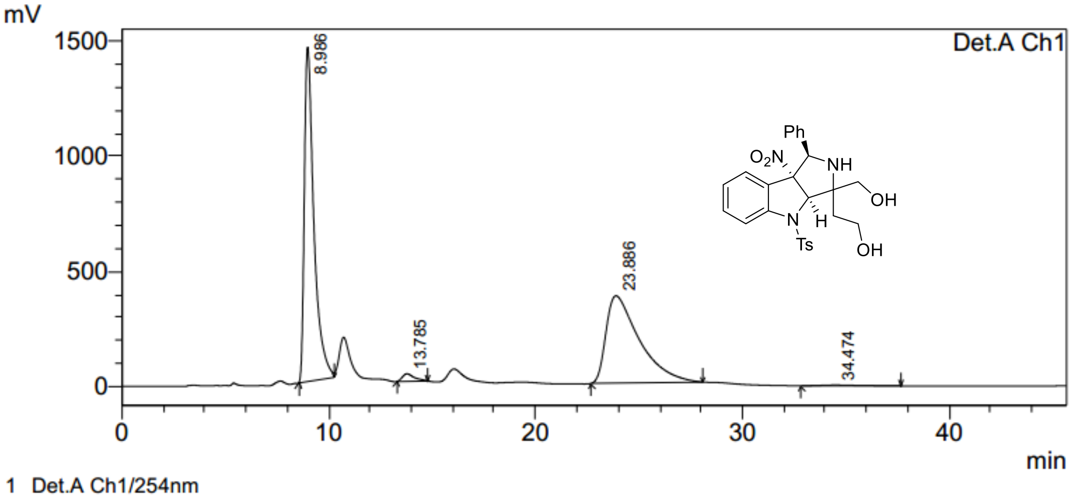

PeakTable

Detector A Ch1 254nm

Peak#	Ret. Time	Area	Height	Area %
1	8.986	46798281	1454539	51.457
2	13.785	1360330	33236	1.496
3	23.886	42368173	378878	46.586
4	34.474	420003	3258	0.462
Total		90946787		100.000

^{1}H NMR (400 MHz, DMSO-d_6) and ^{13}C NMR (101 MHz, DMSO-d_6) of 8

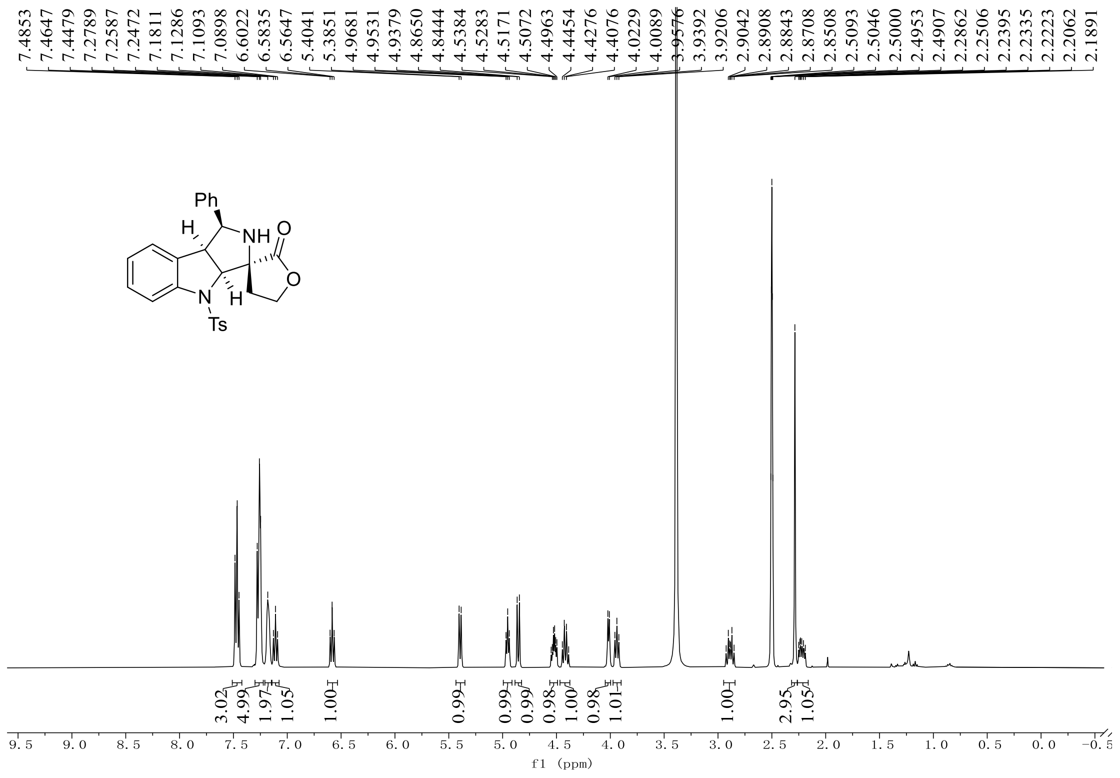

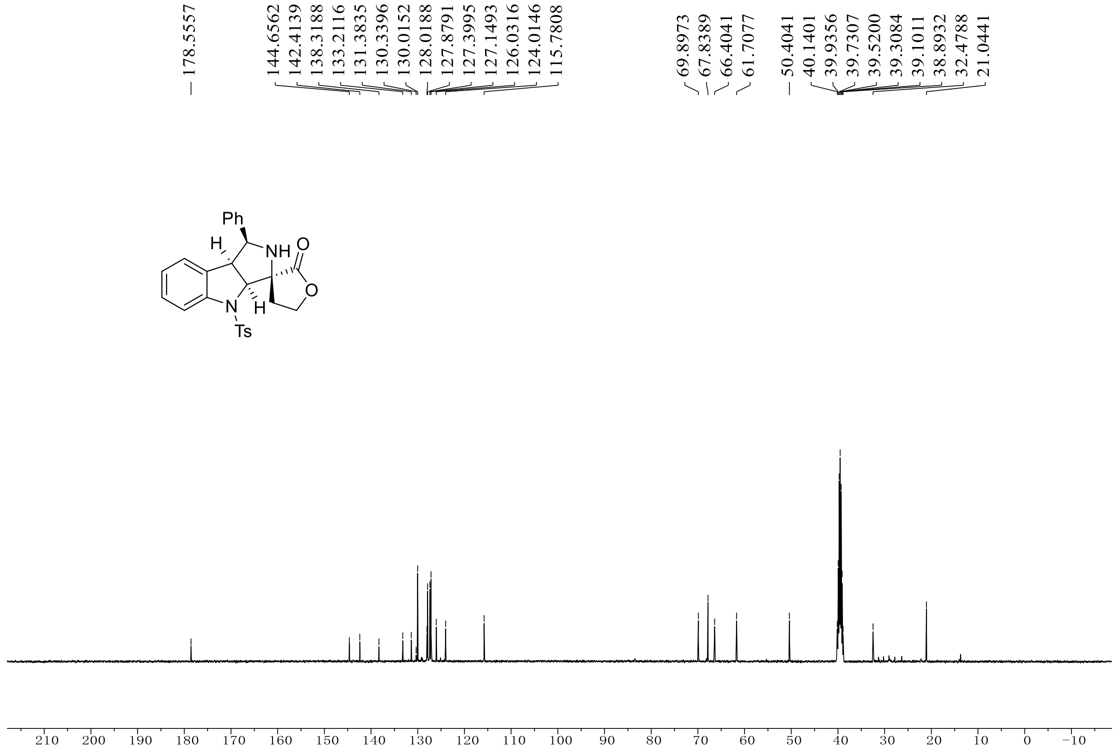

HPLC spectra of 8

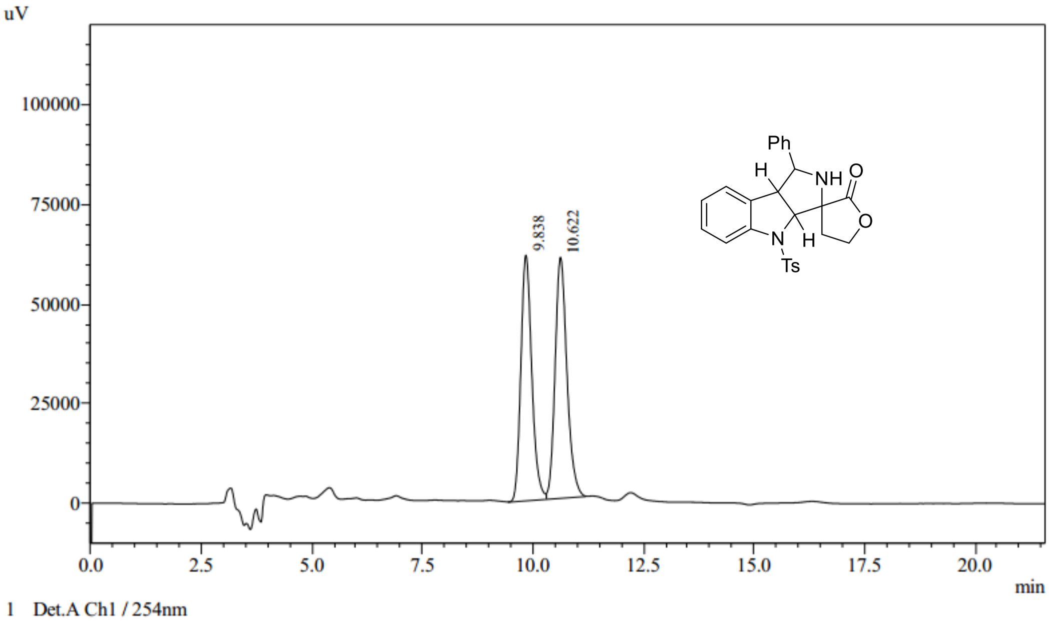

Detector A Ch1 254nm

Peak#	Ret. Time	Area	Height	Area %	Height %
1	9.838	1058308	61659	48.371	50.460
2	10.622	1129602	60535	51.629	49.540
Total		2187911	122194	100.000	100.000

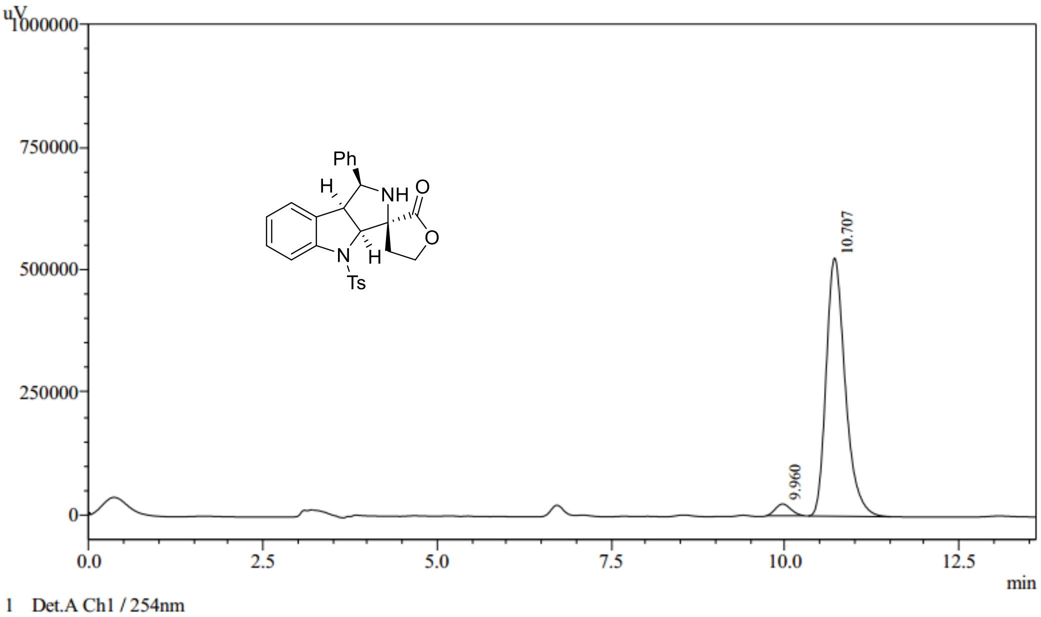

Detector A Ch1 254nm

Peak#	Ret. Time	Area	Height	Area %	Height %
1	9.960	365455	23760	3.507	4.317
2	10.707	10054332	526620	96.493	95.683
Total		10419787	550381	100.000	100.000

^{1}H NMR (400 MHz, DMSO-d_6) and ^{13}C NMR (101 MHz, DMSO-d_6) of 9

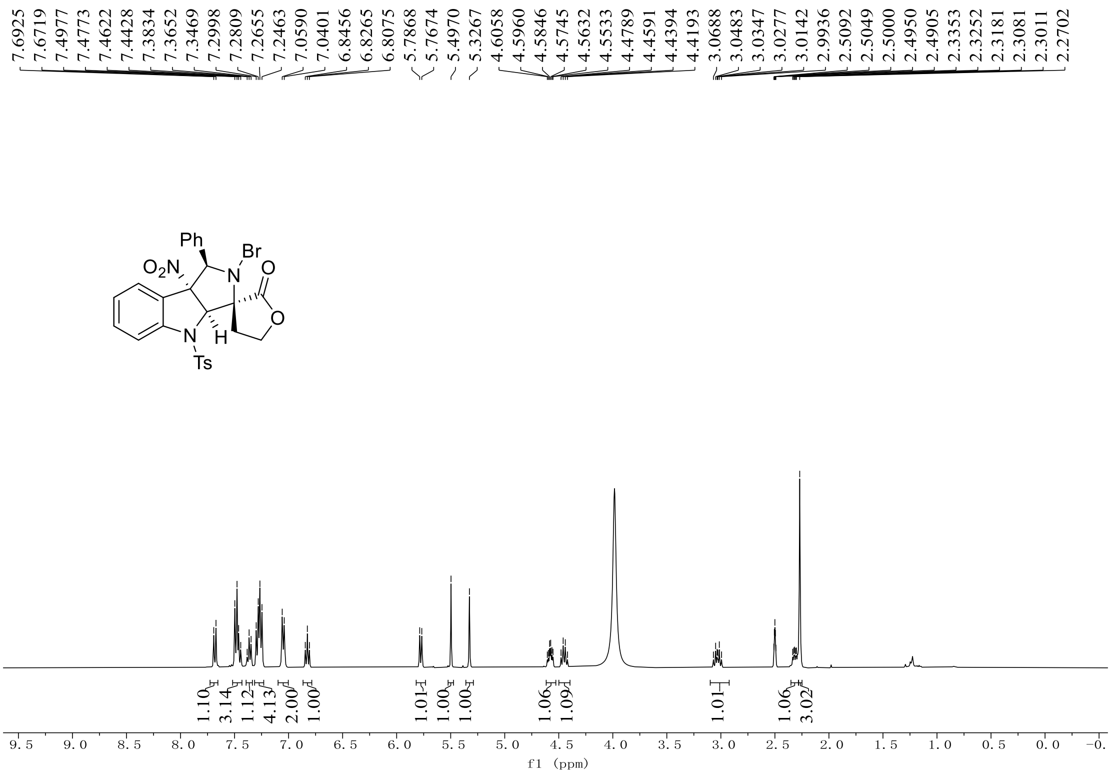

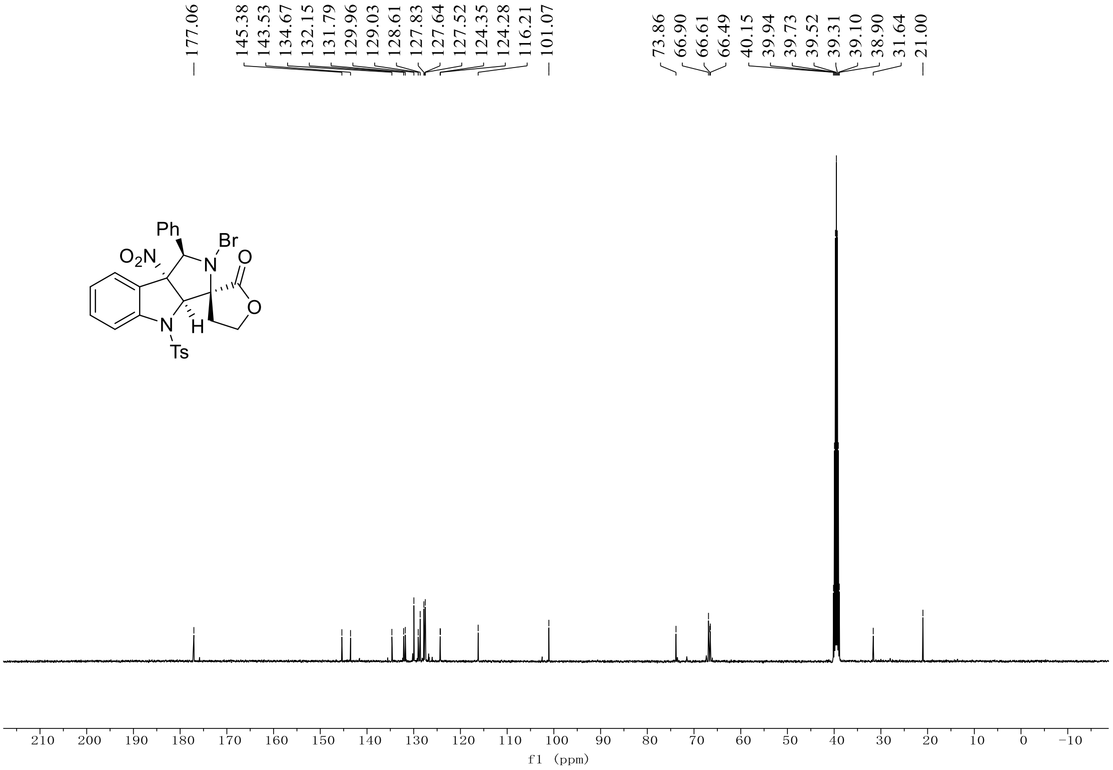

HPLC spectra of 9

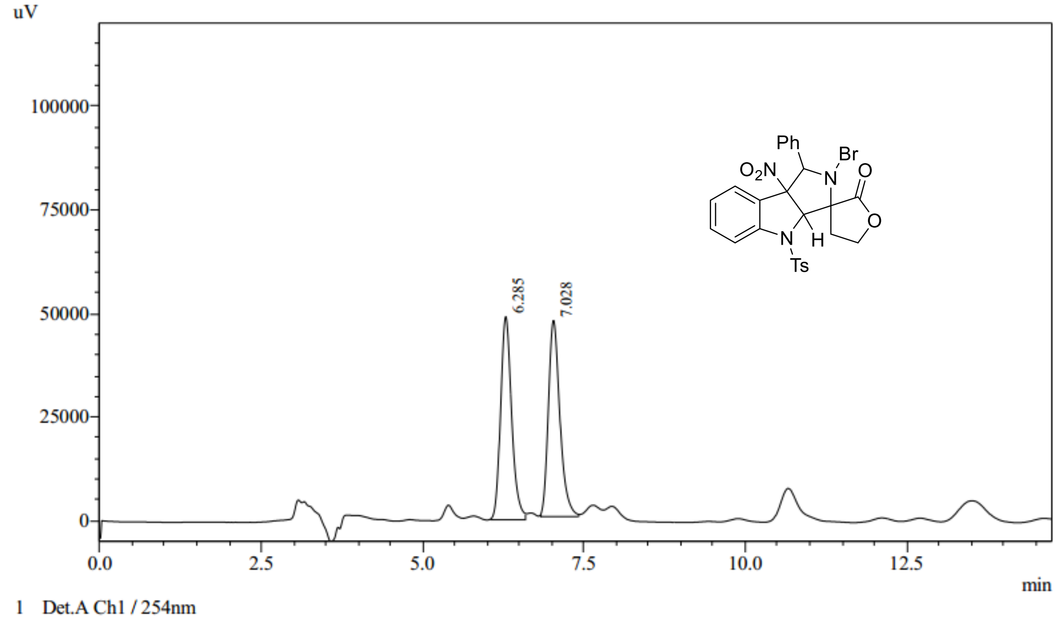

Detector A Ch1 254nm

Peak#	Ret. Time	Area	Height	Area %	Height %
1	6.285	586234	49004	49.899	50.855
2	7.028	588599	47357	50.101	49.145
Total		1174833	96361	100.000	100.000

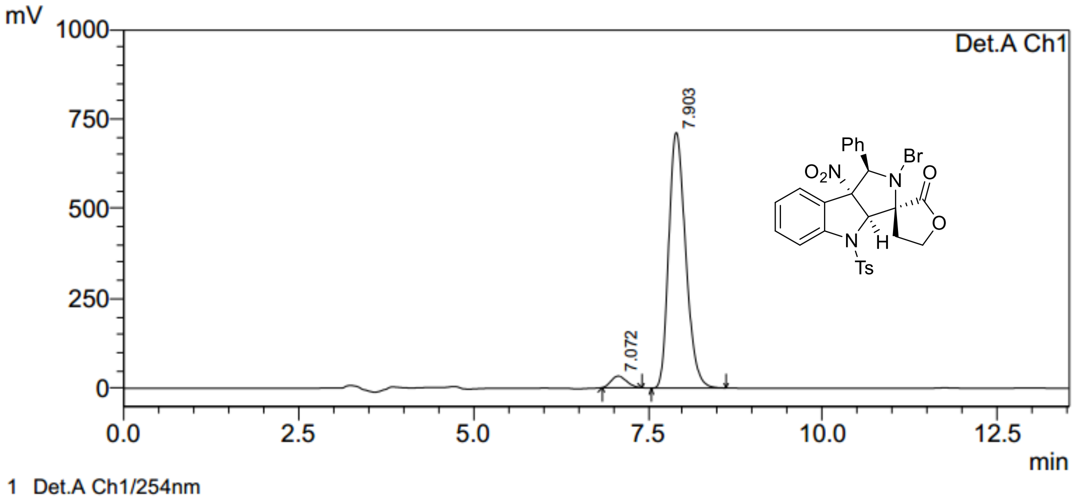

PeakTable

Detector A Ch1 254nm

Peak#	Ret. Time	Area	Height	Area %
1	7.072	489816	32126	3.928
2	7.903	11980998	711253	96.072
Total		12470814		100.000
